# Continuous-time noise mitigation in analogue quantum simulation

**DOI:** 10.1038/s41467-026-78310-5

**Published:** 2026-10-12

**Authors:** Gabriele Bressanini, Yue Ma, Michael Hanks, Hyukjoon Kwon, M. S. Kim

**Affiliations:** 1 Blackett Laboratory, Imperial College London, London, UK; 2 Korea Institute for Advanced Study, Seoul, South Korea

**Keywords:** Quantum simulation, Quantum information

## Abstract

Analogue quantum simulators offer a promising route to explore quantum many-body dynamics beyond classical reach in the near term. However, their vulnerability to noise limits the accuracy of simulations. Here, we establish a continuous-time framework for mitigating noise in analogue quantum simulation. Our protocol fully preserves the continuous-time nature by avoiding any active control or measurement of the system during the dynamics, while achieving exact noise cancellation. This requires a small number of ancillary qubits, whose interaction with the system, combined with classical post-processing of joint measurement data, is tailored to cancel the effect of noise. Furthermore, the protocol is Hamiltonian-independent and robust to realistic ancilla noise. This work points to a promising direction for achieving high-fidelity analogue quantum simulation in the presence of noise.

## Abstract (web summary)

Mitigating errors in analogue quantum simulators - that is, reducing noise-induced bias in expectation values - would greatly improve their operation and scalability. Here, the authors propose a time-continuous error cancellation protocol, based on introducing an auxiliary system and engineering the joint dissipation of the new, extended system.

## Introduction

One of the most promising applications of quantum technology—originally envisioned by Feynman^1^—is the efficient simulation of the dynamics of highly correlated many-body quantum systems, which are central to problems in physics, chemistry and materials science^2–5^. A typical quantum simulation task involves preparing an initial state, evolving it under a target dynamics, and measuring the expectation value of a relevant observable. The target dynamics is, in most cases, the unitary evolution generated by a Hamiltonian, though certain applications require the simulation of non-unitary open system dynamics governed by a Lindblad equation^6^. Digital, gate-based quantum computers, combined with quantum error correction (QEC), will eventually achieve fault-tolerance and enable reliable simulation of arbitrary Hamiltonian dynamics. Nevertheless, despite promising recent results^7–11^, large-scale QEC remains challenging for current devices. On the other hand, analogue quantum simulators offer a realistic path to beyond-classical results in the near term, even without achieving fault-tolerant universal quantum computing. They can already leverage current hardware capabilities, provided that the target Hamiltonian can be mapped onto the simulator’s Hamiltonian, either exactly or with controllable error. Moreover, while digital quantum simulators require discretisation of time evolution, typically via Trotterization, which can introduce systematic errors and increase compilation overhead^12,13^, analogue simulators naturally realise continuous-time dynamics. This may provide substantial near-term advantages in simulating larger systems over longer times, with experimental proposals spanning a variety of physical platforms such as cold atoms in optical lattices, trapped ions and superconducting qubits^14–23^. A major challenge in analogue simulators is their inherent lack of error correction, which limits the size and timescale of simulated systems, as well as the achievable accuracy of measurement outcomes.

As an alternative, a feasible near-term approach in analogue quantum simulators would be quantum error mitigation (QEM)^24^, which focuses on reducing noise-induced bias in expectation values–often the key quantities of interest in present-day experiments–through repeated noisy circuit sampling and classical post-processing, making it suited to noisy intermediate-scale quantum (NISQ) devices. However, most QEM protocols found in the literature are explicitly tailored to discrete, gate–based digital simulators, where noise is assumed to act either immediately before or immediately after each gate; a prominent example is given by probabilistic error cancellation (PEC)^25^. While significant effort has been devoted to understanding how errors accumulate over time in observable expectation values measured on analogue quantum simulators^26–29^, no QEM protocol that is both natively analogue and truly time-continuous has yet been proposed. A stochastic QEM protocol inspired by PEC^30^ requires interleaving the simulator’s time evolution with impulsive recovery operations at predetermined time sequences, which is more naturally suited to hybrid analogue-digital architectures. A recently proposed trajectory-based error mitigation scheme^31^ utilises repeated measurement and reinitialization of an ancilla qubit for continuously monitoring quantum jumps, but this induces stochastic state updates that are not fully compatible with the uninterrupted continuous-time dynamics of analogue quantum simulators.

**Fig. 1:**
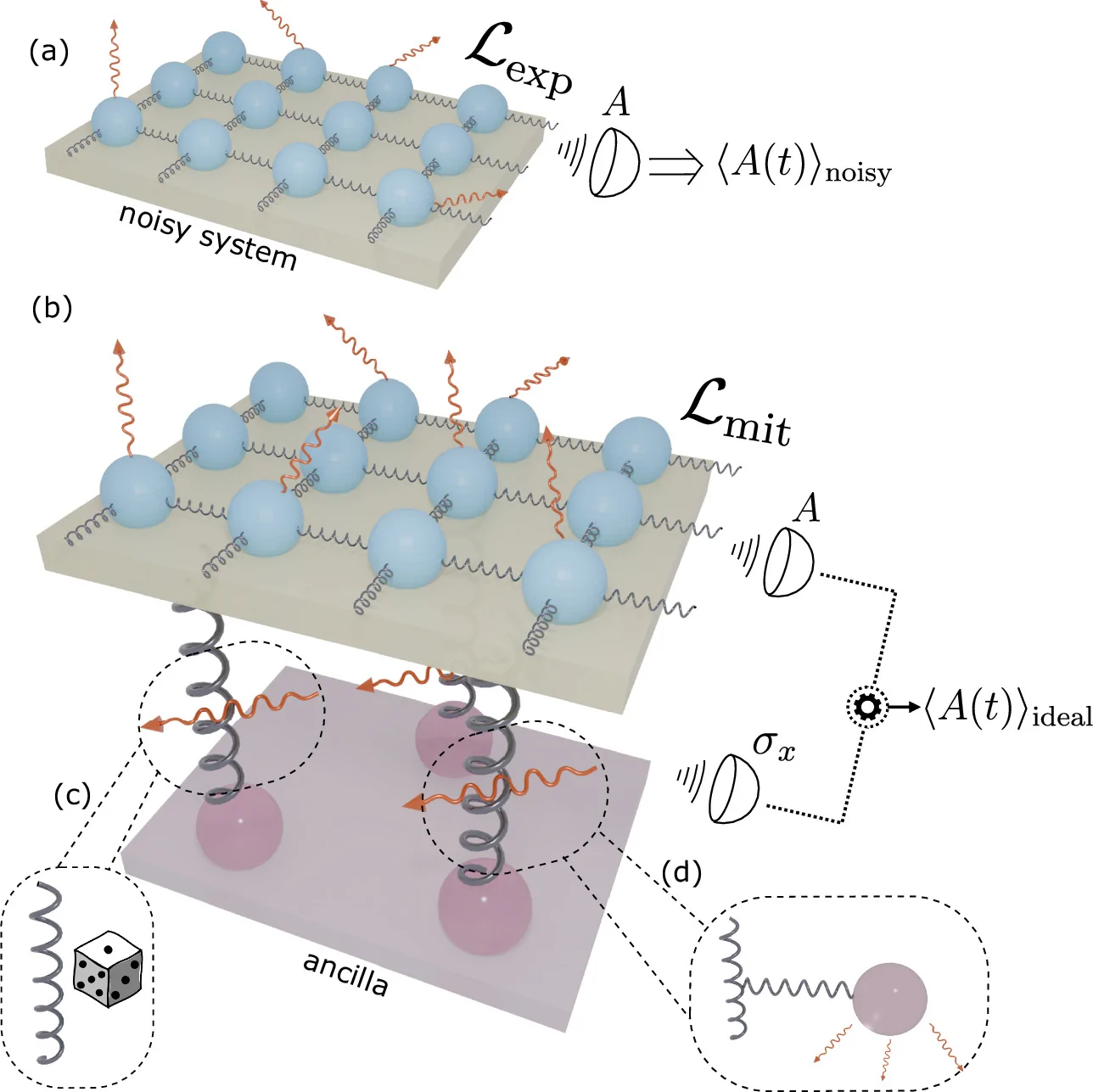
Schematics of the noise mitigation protocol and its implementation. **a** A quantum system evolves under noisy experimental dynamics described by the Lindbladian ${{{\mathcal{L}}}}_{\exp }$ in Eq. 1 for time *t*, and then an observable *A* is measured. **b** In the mitigation scheme, the system is coupled to one or more ancillary qubits, each prepared in the state $\left\vert+\right\rangle$ and evolves under the joint dissipation, described by ${{{\mathcal{L}}}}_{{{\rm{mit}}}}$ in Eq. 3. After evolving the joint system to time *t*, joint measurements of *A* on the system and *σ*_*x*_ on the ancillae are combined through classical post-processing to yield an unbiased estimation of the noise-free expectation value 〈*A*(*t*)〉_ideal_. The joint dissipation between system and ancilla can be implemented either using **c** a stochastic Hamiltonian or **d** coupling to an additional fast-decaying ancilla.

In this work, we introduce a fully autonomous time-continuous error-cancellation protocol designed specifically for analogue quantum simulators. Our method is native to the analogue setting, as it preserves the system’s continuous-time evolution without any interleaving pulse operations or intermediate measurements by operating entirely within the framework of Markovian open-system dynamics, described by the Lindblad master equation. The protocol consists of coupling the system to a small number of ancillary qubits, with a single qubit being sufficient in principle, and allowing the system-ancilla state to evolve under the Lindblad master equation involving additional joint jump operators. The joint state is then measured, and a classical post-processing step is applied to recover the ideal noiseless expectation value of an arbitrary system observable (see Fig. 1). Remarkably, the proposed protocol is also robust against typical and experimentally relevant forms of noise affecting the ancilla qubit.

The proposed protocol offers two key advantages over previously introduced schemes^30,31^, as it neither relies on gate-based control through interleaved pulse operations, nor requires intermediate measurements on the system or the ancilla during the evolution. In addition, the protocol is entirely independent of the specific Hamiltonian being simulated, ensuring universality across diverse quantum simulation tasks and physical platforms. We discuss the physical implementation of our protocol and show that, for a broad class of commonly employed noise models, it can be readily realised via ensemble averaging over a stochastic Hamiltonian. We demonstrate the protocol’s effectiveness through three representative examples: the Heisenberg model, the Loschmidt echo following a quantum quench, and the Floquet dynamics of a periodically driven spin chain. Our approach opens a new pathway toward high-fidelity analogue quantum simulations resilient to decoherence.

## Results

### Framework

Let us consider a finite-dimensional system initialised in state *ρ*(0) at *t* = 0, whose evolution is governed by the following Lindblad master equation

1 $$\dfrac{d}{dt}\,\rho=-i\left[H,\rho \right]+{\sum}_{k}{{\mathcal{D}}}\left[{L}_{k}\right](\rho )\equiv {{\mathcal{H}}}(\rho )+{{\mathcal{D}}}(\rho )\equiv {{{\mathcal{L}}}}_{\exp }(\rho )\,,$$

where ${{\mathcal{D}}}[L](\cdot )=L\cdot {L}^{{\dagger} }-\frac{1}{2}\{{L}^{{\dagger} }L,\cdot \}$ denotes the dissipator superoperator. The Lindbladian ${{{\mathcal{L}}}}_{\exp }$ models a typical experimental dynamics with Markovian noise, where *H* is a target Hamiltonian of interest, and *L*_*k*_ are jump operators corresponding to noise that results from the interaction between the system and the environment. The expectation value of a quantum observable *A* at time *t* reads ${\langle A(t)\rangle }_{{{\rm{noisy}}}}={{\rm{Tr}}}[A{e}^{{{{\mathcal{L}}}}_{\exp }t}(\rho (0))]$, which is inherently affected by the noise. The central question we address—and answer affirmatively—is whether it is possible to eliminate the effects of noise and recover the ideal, noiseless expectation value ${\langle A(t)\rangle }_{{{\rm{ideal}}}}={{\rm{Tr}}}[A{e}^{-iHt}\rho (0){e}^{iHt}]$.

The effects of noise and the Hamiltonian do not, in general, commute with each other, i.e. ${e}^{{{\mathcal{H}}}t+{{\mathcal{D}}}t}\ne {e}^{{{\mathcal{D}}}t}{e}^{{{\mathcal{H}}}t}$. As a result, noise can propagate non-trivially throughout the dynamics, rendering the error-mitigation task particularly challenging.

### Noise mitigation protocol

We present our continuous-time noise mitigation protocol, which is independent of both the system Hamiltonian and the observable of interest *A*. As the mitigation Lindbladian is independent of *H*(*t*), our protocol can be generalised by replacing *e*^−*iHt*^ with the corresponding time-ordered exponential ${{\mathcal{T}}}[e^{-i\int_{0}^{t}H(\tau )d\tau }]$ for a time-dependent system Hamiltonian *H*(*t*) without modification. The protocol requires only a single-qubit ancilla, regardless of the system’s dimension. We aim to perfectly cancel the noisy contributions to the experimental dynamics in Eq. 1 by introducing an additional *negative* dissipator, i.e. $-{{\mathcal{D}}}(\rho )$, leaving us with the ideal unitary dynamics generated by the Schrödinger equation $\dot{\rho }=-i[H,\rho ]$. However, the negative sign in front of this superoperator renders the latter non-physical and therefore not directly implementable.

We overcome this problem by introducing a qubit ancilla that interacts with the system through joint dissipation, inspired by techniques that encode the system’s dynamics in the off-diagonal blocks of the system-ancilla state^32–34^. Upon projecting the entire system-ancilla state after their joint dissipation onto the subspace spanned by the off-diagonal elements in the ancilla’s computational basis, we obtain an operator that evolves according to the noiseless Schrödinger equation of the system.

For an explicit description of the protocol, let *W* be the system-ancilla joint density matrix, and consider the following Lindblad master equation

2 $$\dfrac{d}{dt}W={{{\mathcal{L}}}}_{\exp }(W)+{{{\mathcal{L}}}}_{{{\rm{mit}}}}(W)\,.$$

Here, ${{{\mathcal{L}}}}_{\exp }$ acts trivially on the ancilla qubit and ${{{\mathcal{L}}}}_{{{\rm{mit}}}}$ denotes the mitigation Lindbladian

3 $${{{\mathcal{L}}}}_{{{\rm{mit}}}}={\sum}_{k}{{\mathcal{D}}}\left[{L}_{k}\otimes {\sigma }_{z}\right]+{{\mathcal{D}}}\left[\sqrt{S}\otimes {\sigma }_{z}\right]+{{\mathcal{D}}}\left[\sqrt{S}\otimes {\mathbb{I}}\right]\,,$$

where ${\mathbb{I}}$ and ${\sigma }_{z}=\left\vert 0\right\rangle \left\langle 0\right\vert -\left\vert 1\right\rangle \left\langle 1\right\vert$ are the identity and the Pauli *Z* operators, respectively, and $S=a{\mathbb{I}}-{\sum}_{k}{L}_{k}^{{\dagger} }{L}_{k}\ge 0$, with $a={\lambda }_{\max }({\sum}_{k}{L}_{k}^{{\dagger} }{L}_{k})$ being the largest eigenvalue of ${\sum}_{k}{L}_{k}^{{\dagger} }{L}_{k}$. For dissipators acting locally on distinct subsystems, *a* can be directly evaluated as the sum of the various *a*_*ℓ*_’s on the *ℓ*-th local subsystems, $a={\sum}_{\ell }{a}_{\ell }$. Especially for dissipators satisfying ${L}_{k}^{{\dagger} }{L}_{k}={\gamma }_{k}{\mathbb{I}}$ (e.g. Pauli operators) with rate *γ*_*k*_, we have an even simpler formula, $a={\sum}_{k}{\gamma }_{k}$. For non-local dissipators, finding the largest eigenvalue of the component dissipators is equivalent to a ground-state calculation on ${\sum}_{k}{L}_{k}^{{\dagger} }{L}_{k}$, aided by symmetry assumptions where they apply. For the most physically relevant cases, the dissipators are local or well-structured, so that the calculation of *a* does not induce computational challenge.

We now project Eq. 2 onto the computational basis of the ancilla qubit, defining $W_{ij} \equiv \left\langle i \right| W\left\vert j\right\rangle$, where *i*, *j* ∈ {0, 1}. These four operators act on the system Hilbert space, and satisfy the following equations (see 'Methods' for a detailed derivation)

4 $$\frac{d}{dt} W_{ij}=- i \left[H,{W_{ij}}\right] - 2aW_{ij},$$

5 $$\dfrac{d}{dt} W_{ii}=-i \left[H,W_{ii}\right]+2{\sum}_{k}{{\mathcal{D}}}\left[{L}_{k}\right]({W_{ii}})+2{{\mathcal{D}}}\left[\sqrt{S}\right]({W_{ii}}),$$

for *i* ≠ *j*. We observe that the joint dissipators introduced by ${{{\mathcal{L}}}}_{{{\rm{mit}}}}$ have the effect of removing the original system noise terms from the off-diagonal blocks, Eq. 4, while doubling the noise contributions in the diagonal ones, Eq. 5. In particular, Eq. 4 coincides with the noiseless Schrödinger equation, up to an additional exponential decay term given by −2*aW*_*ij*_. Consequently, if we initialise the system and ancilla in the state $W(0)=\rho (0)\otimes \left\vert+\right\rangle \left\langle+\right\vert$, with $\left\vert+\right\rangle=(\left\vert 0\right \rangle+\left\vert 1\right\rangle)/\sqrt{2}$, the solution to Eq. 4 becomes

6 $$W_{01}(t)=\dfrac{e^{-2at}}{2} e^{-iHt} \rho(0) {e^{iHt}}=W_{10}(t).$$

Hence, *W*_*ij*_(*t*) corresponds to the ideal system state evolved under the noise-free unitary dynamics, up to an exponential damping prefactor. Finally, as shown in Methods, we can express the ideal expectation value of *A* as

7 $${\left\langle {A}({t})\right\rangle }_{{{\rm{ideal}}}}={e}^{2at}{{\rm{Tr}}}\left[({A}\otimes {{\sigma }_{x}})W(t)\right]\,,$$

where ${\sigma }_{x}=\left\vert 0\right\rangle \left\langle 1\right\vert+\left\vert 1\right\rangle \left\langle 0\right\vert$ denotes the Pauli *X* operator. We can estimate the decay rate *a* by setting $A={\mathbb{I}}$, since $1={\langle {\mathbb{I}}(t)\rangle }_{{{\rm{ideal}}}}={e}^{2at}{{\rm{Tr}}}[({\mathbb{I}}\otimes {\sigma }_{x})W(t)].$ In other words, evaluating ${{\rm{Tr}}}[({\mathbb{I}}\otimes {\sigma }_{x})W(t)]$ directly gives information about the decay exponent, which we then use to express a mitigated expectation value as

8 $${\left\langle A(t)\right\rangle }_{{{\rm{ideal}}}}=\dfrac{{{\rm{Tr}}}\left[(A\otimes {\sigma }_{x})W(t)\right]}{{{\rm{Tr}}}\left[({\mathbb{I}}\otimes {\sigma }_{x})W(t)\right]}\,.$$

If the noise operators have explicit time dependence, the previous derivation still holds, with the modification that *a* is promoted to a function of time, defined as $a(t)={\lambda }_{\max }({\sum}_{k}{L}_{k}^{{\dagger} }(t){L}_{k}(t))$. In this case, the ideal expectation value can be written as ${\langle A(t)\rangle }_{{{\rm{ideal}}}}=e^{2\int_{0}^{t}a(s)ds}{{\rm{Tr}}}[(A\otimes {\sigma }_{x})W(t)]$.

As a special case, if the noise operators satisfy ${\sum}_{k}{L}_{k}^{{\dagger} }{L}_{k}\propto {\mathbb{I}}$, setting $a={\lambda }_{\max }({\sum}_{k}{L}_{k}^{{\dagger} }{L}_{k})$ causes *S* to vanish, reducing Eq. 3 to the simpler form

9 $${{{\mathcal{L}}}}_{{{\rm{mit}}}}={\sum}_{k}{{\mathcal{D}}}\left[{L}_{k}\otimes {\sigma }_{z}\right]\,.$$

In this case, the mitigation Lindbladian can be implemented even without explicitly computing *a*. A typical example of such a situation is when each noise operator is a single-qubit Pauli operator. This also greatly enhances experimental implementability, as we only need to realise each pair of joint dissipations between a single system qubit and the ancilla qubit.

We can summarise the protocol for obtaining the noiseless expectation value of an arbitrary observable *A* at time *t*—given that the system evolves according to the noisy dynamics in Eq. 1—as follows:

1. Prepare the ancilla in the $\left\vert+\right \rangle$ state,
2. Implement the joint system Lindblad evolution described by Eq. 2 for the same time *t*,
3. Evaluate the expectation value of the observable *A* ⊗ *σ*_*x*_ for the joint system,
4. Multiply the result by the prefactor *e*^2*at*^.

While, in principle, our protocol only requires a single ancilla qubit, such an implementation may be experimentally challenging, as the ancilla must interact with all system qubits. This requirement can be difficult to satisfy in platforms with limited connectivity, and may demand the engineering of non-trivial many-body interactions. To address these constraints, in Supplementary Note 2, we explore several equivalent alternative realisations of our scheme, including protocols employing multiple ancilla qubits, up to the limiting case where each system qubit is paired and interacts with its own ancilla, as well as extensions involving qutrit ancillae and/or modified dissipative couplings. We further discuss at the end of the following section how introducing multiple ancilla qubits could also reduce the sampling overhead with respect to the system size.

### Sampling overhead

As is typical with error mitigation techniques, removing bias comes at the cost of increased variance^24^ in the estimator of the expectation value. From Eq. 7, it is clear that the protocol’s sampling overhead, defined as the scaling factor between the variance of the mitigated estimator and that of the unmitigated (noisy) estimator at a fixed number of measurement shots *n*, originates from the exponential pre-factor *e*^2*at*^.

The noisy estimator of the expectation value of *A* at time *t* simply reads ${\hat{A}}_{{{\rm{noisy}}}}(t)={n}^{-1}{\sum }_{i=1}^{n}{A}_{i}(t)$, where *A*_*i*_(*t*) denotes the measurement outcome from the *i*th shot and the system evolving according to Eq. 1 up to time *t*. On the other hand, the mitigated unbiased estimator is ${\hat{A}}_{{{\rm{mit}}}}(t)={n}^{-1}{e}^{2at}{\sum }_{i=1}^{n}{A}_{i}(t){\sigma }_{x,i}(t)$, where *A*_*i*_(*t*)*σ*_*x*,*i*_(*t*) denotes the *i*th measurement outcome of the observable *A* ⊗ *σ*_*x*_ on *W*(*t*). Therefore, assuming that the per-shot variances are comparable in both the mitigated and unmitigated scenarios, the sampling overhead is approximately *e*^4*at*^. This is consistent with the general QEM tradeoff that suppressing bias by post-processing typically amplifies estimator variance, where exponential sampling costs with respect to the evolution time *t* are known explicitly for probabilistic error cancellation^24,25^ and are also implied, in worst-case settings, by general sampling lower bounds for broad classes of QEM protocols^35–37^. Applying Hoeffding’s inequality, one finds the number of samples required to ensure the mitigated estimator is *ϵ*-close to its expectation value 〈*A*(*t*)〉_ideal_ with probability no less than 1 − δ scales as $n\propto {e}^{4at}{{\mathrm{log}}}(2/{\delta })/{\epsilon }^{2}$.

For an *N*-qubit system subject to local noise on each qubit, a naive application of the proposed mitigation protocol incurs a sampling overhead of ${e}^{4N{a}_{1}t}$, where *a*_1_ is the rescaling factor for the single-qubit case. Nevertheless, this overhead can be significantly reduced for a local observable supported on a block with small Pauli weight by introducing multi-flag ancillary qubits, in which case the weight-resolved scaling of the sampling overhead applies. One can further decompose the noisy Lindbladian into Schur-irreducible blocks, and projecting onto these blocks yields an even lower sample complexity (see 'Methods' and Supplementary Note 3 for details).

### Noise model requirements

The protocol requires a learned or physically motivated representation of the dominant jump operators *L*_*k*_. This requirement is analogous to the noise-characterisation assumptions in other QEM methods^24^. For example, PEC^25^ requires a characterised noise map in order to construct an inverse quasiprobability representation, and recent large-scale PEC implementations learn sparse Pauli-Lindblad noise models for this purpose. This can be done by master-equation reconstruction protocols, which have been demonstrated experimentally in NMR^38^, trapped-ion^39^ and superconducting-qubit platforms^40^. When the relevant dissipator is sparse, local, or Pauli-twirled, the required information can be obtained by established characterisation methods such as randomised compiling^41^, cycle benchmarking^42^ and sparse Pauli-Lindblad learning^43,44^. This can be further combined with recently proposed compressed-sensing^45^ or shadow-based^46^ Lindblad tomography.

### Protocol robustness against imperfect noise characterisation

An important and practical aspect to investigate is the protocol’s robustness to imperfect noise characterisation. Specifically, we assume that the Lindblad noise operator is known, but the associated rate *γ* is only approximately estimated, with $\tilde{\gamma }=\gamma+\epsilon$ denoting the estimated rate and *ϵ* a small error. We also assume perfect implementation of any desired Lindbladian. In the following, we separately examine how imperfect knowledge of the noise, whether on the system or the ancilla, affects the accuracy of our protocol. We begin by analysing the impact of imperfect knowledge of the system’s noise. Let us assume that the noise is modelled by a single Lindblad noise operator $\sqrt{\gamma }L$, for which we have learned an approximation $\sqrt{\tilde{\gamma }}L$. Straightforward calculations yield the evolution equation for *W*_*ij*_

10 $$\dfrac{d}{dt}{W_{ij}}=-i\left[H,{W_{ij}}\right]-\epsilon D\big[L\big]({W_{ij}})-2a{W_{ij}},$$

showing that, although perfect noise cancellation is not achieved, an accurate estimate of *γ* ensures that most of the noise is effectively mitigated. Eq. 10 implies that, in this scenario, our protocol yields estimates of expectation values corresponding to the system’s dynamics being governed by a master equation of the same form as Eq. 1. Interestingly, when *ϵ* < 0, the master equation reduces to the initial Lindblad equation, but with an effective noise rate of $\gamma -\tilde{\gamma }$. On the other hand, if *ϵ > 0*, the resulting equation corresponds to a non-physical, pseudo-Lindblad equation, which describes non-Markovian dynamics.

We now turn our attention to the case where the ancilla noise rate is only approximately known. As before, the true noise operator is $\sqrt{\gamma }M$, while our learned approximation is $\sqrt{\gamma+\epsilon }M$. Assuming that *M* is a correctable noise operator, ${{{\mathcal{L}}}}_{{{\rm{mit}}}}$ remains unchanged, and the only adjustment is a modification of the exponential pre-factor, resulting in an overall multiplicative pre-factor that scales like *e*^*νϵt*^, for some noise-dependent constant *ν*.

### Protocol robustness against ancilla noise

In any realistic implementation of the protocol, the ancilla qubit would also be subject to noise. Therefore, it is crucial to evaluate the effect of this additional noise source on our protocol. Remarkably, we show that a simple rescaling of the measurement outcomes can correct for most ancilla noise models. To illustrate this, let us consider a single noise operator *M* acting on the qubit ancilla. The Lindblad equation governing the evolution of the system-ancilla joint state is then given by

11 $$\dfrac{d}{dt}{W}={{{\mathcal{L}}}}_{\exp }({W})+{{{\mathcal{L}}}}_{{{\rm{mit}}}}({W})+{{{\mathcal{L}}}}_{{{\rm{anc}}}}({W})\,,$$

with ${{{\mathcal{L}}}}_{{{\rm{anc}}}}(W)=\Gamma {{\mathcal{D}}}[{\mathbb{I}}\otimes M](W)$, where *Γ* is the ancilla noise rate. Consequently, the differential equation that governs the evolution of *W*_*ij*_ described in Eq. 4 now contains the additional term

12 $${\Gamma} \left\langle i\left| {{\mathcal{D}}}\left[{{\mathbb{I}}}\otimes {M}\right]{(W})\right| j\right\rangle.$$

Although the operator *M* generally depends on the specific physical implementation of the qubit, we can nonetheless compute this additional contribution for widely used and realistic noise models, such as dephasing (*σ*_*z*_ or $\left\vert 0\right\rangle \left\langle 0\right\vert$ and $\left\vert 1\right\rangle \left\langle 1\right\vert$), amplitude damping (*σ*_−_), thermal excitation (*σ*_+_) or Pauli Lindblad noise channels (*σ*_*x*_, *σ*_*y*_, *σ*_*z*_). These noise operators essentially encompass all the commonly used models for describing single-qubit noise in practical settings.

By substituting all these noise operators into Eq. 12, we find that the contribution takes the form −*νΓW*_*ij*_, where *ν* is a non-negative constant determined by the specific jump operator. In particular, the contribution vanishes (*ν* = 0) for *σ*_*x*_, while we obtain *ν* = 2 for *σ*_*z*_ and *σ*_*y*_, and finally we have *ν* = 1/2 for the remaining operators ${\sigma }_{\pm },\left\vert 0\right\rangle \left\langle 0\right\vert,$ and $\left\vert 1\right\rangle \left\langle 1\right\vert$.

As a result, even with ancilla noise, Eq. 4 maintains the form of a Schrödinger equation with exponential damping, the only modification being an increased decay constant. Crucially, the recovery protocol remains unchanged: ${{{\mathcal{L}}}}_{{{\rm{mit}}}}$ is unaffected by the ancilla noise, which is instead accounted for by means of a simple classical post-processing, namely an appropriate redefinition of the exponential pre-factor ${e}^{2at}\mapsto {e}^{2\tilde{a}t}$ in Eq. 7, which only affects the sampling overhead of the scheme. We stress that only the exponential pre-factor is modified, while the parameter *a* appearing in the operator *S* within ${{{\mathcal{L}}}}_{{{\rm{mit}}}}$ remains unchanged.

If the ancilla qubit is affected by multiple noise processes corresponding to different noise operators from the ones described above, the mitigation scheme remains valid, provided that the effective decay rate $\tilde{a}$ is adjusted to account for the combined contributions of all ancilla noise operators. Once again, we can estimate the effective decay rate $\tilde{a}$ via ${e}^{-2\tilde{a}t}={{\rm{Tr}}}[({\mathbb{I}}\otimes {\sigma }_{x})W(t)]$, hence Eq. 8 still holds. In 'Methods', we also analyse the robustness of the protocol against coherent ancilla noise and correlated noise, where the latter naturally arises from system-ancilla crosstalk.

### Physical implementation

We first consider the case where the system’s noise jump operators *L*_*k*_ are Hermitian and unitary, such as Pauli operators, and the mitigation protocol is defined by the jump operators in Eq. 9. This scenario is particularly relevant for current quantum hardware, where Pauli noise constitutes a dominant source of errors. In this setting, the effective dynamics generated by each jump operator *L*_*k*_ ⊗ *σ*_*z*_ can be realised by taking the ensemble average over a stochastic Hamiltonian^47,48^,

13 $${H}_{{{\rm{int}}}}({t})=\mathop{\sum}_{k}{\eta }_{k}({t})\left({L}_{k}\otimes {\sigma }_{z}\right),$$

where the coefficients *η*_*k*_(*t*) are independent white noise processes. This stochastic Hamiltonian approach has already been demonstrated experimentally in trapped-ion systems^49^ and superconducting qubits^50^, highlighting the near-term feasibility of testing the scheme in this simplified regime.

Beyond the Pauli noise case, more general joint evolutions of the system and ancilla can be realised by various different dissipation engineering techniques^48,51,52^. A standard way to implement each jump operator of the mitigation Lindbladian ${{{\mathcal{L}}}}_{{{\rm{mit}}}}$ in Eq. 3 is to couple the entire system with another fast-decaying ancilla system with the interaction Hamiltonian^51^.

14 $${H}_{{{\rm{int}}}}\propto {L}_{{{\rm{mit}}}}^{{\dagger} }\otimes {\sigma }_{-}^{(a)}+{L}_{{{\rm{mit}}}}\otimes {\sigma }_{+}^{(a)},$$

where *L*_mit_ is the target jump operator for mitigation, i.e. *L*_*k*_ ⊗ *σ*_*z*_, $\sqrt{S}\otimes {\sigma }_{z}$, and $\sqrt{S}\otimes {\mathbb{I}}$ in Eq. 3, and ${\sigma }_{\pm }^{(a)}$ are the lowering and raising operators of the fast-decaying ancilla state with dissipator ${{\mathcal{D}}}[{\sigma }_{-}^{(a)}]$. While this requires multi-qubit interactions between system and ancillary states, the main ingredients for the proposed dissipation engineering have recently been realised in trapped-ion^53^, quantum dot^54^ and cavity QED^55,56^ systems. Alternatively, by interpreting our ancilla-assisted recovery dynamics as a continuous-time signed erasure channel (see Supplementary Note 4 for details), our protocol belongs to a family whose hardware viability has been independently established by erasure-converted superconducting^57–59^ and neutral-atom^60^ qubits.

We also highlight that only a weakly coupled dissipation strength is required, which scales with the noise rate ∥*L*_mit_∥ = ∥*L*_*k*_ ⊗ *σ*_*z*_∥ = ∥*L*_*k*_∥. This is significantly smaller compared to other dissipation engineering approaches for suppressing noise, e.g. in refs. ^55,56^, where the engineered dissipation strength should be significantly stronger than the noise rate. In Supplementary Notes 5 and 6, we provide more rigorous numerical simulation results to demonstrate the feasibility of the protocol by reflecting more realistic experimental scenarios, including crosstalk and non-Markovian noise.

### Examples

#### Heisenberg model simulation

As a first example, we consider a 2D anisotropic Heisenberg model on a 2 × 2 square lattice, with Hamiltonian

15 $${H}={\sum}_{\langle ij\rangle }\left[{J}_{x}{\sigma }_{x}^{(i)}{\sigma }_{x}^{(j)}+{J}_{y}{\sigma }_{y}^{(i)}{\sigma }_{y}^{(j)}+{J}_{z}{\sigma }_{z}^{(i)}{\sigma }_{z}^{(j)}\right]-h{\sum }_{i=1}^{4}{\sigma }_{y}^{(i)},$$

where 〈*ij*〉 denotes nearest-neighbour pairs, *h* is the strength of the transverse field, and the superscripts indicate the spins on which the operators act. We further take *J*_*x*_ = *J*(1 + *ξ*), *J*_*y*_ = *J*(1 − *ξ*), and *J*_*z*_ = *J* with the anisotropy parameter *ξ* and the strength of the pairwise interaction *J*. Such a model has been extensively studied to investigate quantum magnetism and criticality^61–64^.

We assume that each qubit in the analogue simulator—including both system and ancilla—is subject to dephasing and relaxation noise, modelled by the jump operators $\sqrt{{\gamma }_{z}}{\sigma }_{z}$ and $\sqrt{{\gamma }_{-}}{\sigma }_{-}$, respectively. We are interested in the expectation value of the total magnetisation, i.e. ${{\mathcal{M}}}={\sum }_{i=1}^{4}{\sigma }_{z}^{(i)}$. To construct the jump operators for recovery, we compute the decay constant *a* and the operator $\sqrt{S}$. Specifically, we find $a={\lambda }_{\max }(\mathop{\sum }_{i=1}^{4}({\gamma }_{z}{\sigma }_{z}^{(i){\dagger} }{\sigma }_{z}^{(i)}+{\gamma }_{-}{\sigma }_{-}^{(i){\dagger} }{\sigma }_{-}^{(i)}))=4({\gamma }_{z}+{\gamma }_{-})$ and, using that *S* is diagonal in the computational basis, we may write $\sqrt{S}$ in the following compact form

16 $$\sqrt{S}=\mathop{\sum}_{x\in {\{0,1\}}^{4}}\sqrt{{4}-{h}({x})}\left\vert x\right\rangle \left\langle x\right\vert \,,$$

where *h*(*x*) denotes the Hamming weight of the bit-string *x*, i.e. the number of its non-zero digits. The four system qubits and the ancilla qubit are initialised in the states $\left\vert 0\right \rangle$ and $\left\vert+\right \rangle$, respectively.

**Fig. 2:**
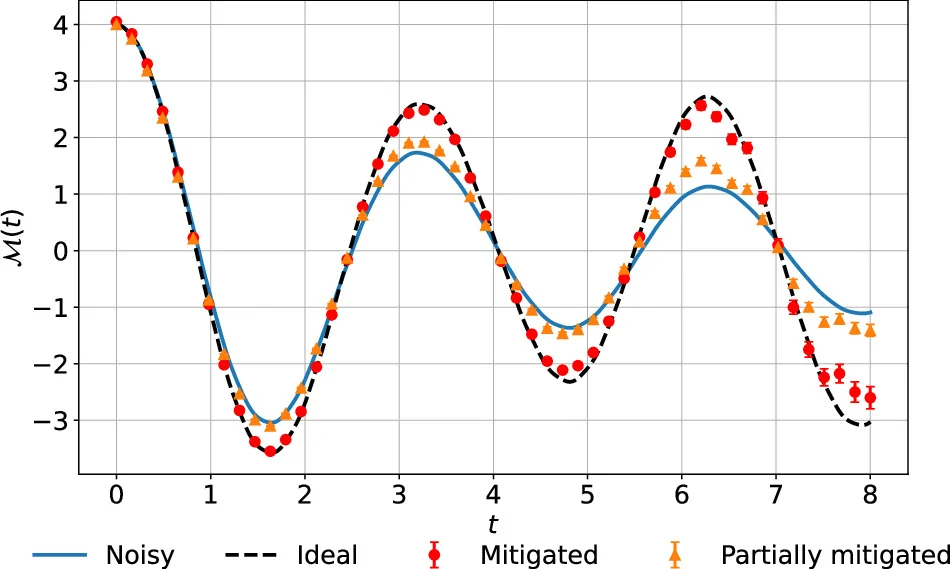
Expectation value of the total magnetisation in the 2D anisotropic Heisenberg model with transverse field and *N* = 4 spins. The mitigated expectation values and their standard deviations (represented by the error bars) are obtained by averaging 10^6^ samples. Partial mitigation corresponds to the correction of system noise only, ignoring ancilla noise.

We let the joint state evolve under Eq. 11, after which we perform a joint measurement of the total magnetisation on the system qubits and *σ*_*x*_ on the ancillary qubit. Using Eq. 7, the noise-free expectation value of ${{\mathcal{M}}}$ can be expressed as

17 $${\left\langle {{\mathcal{M}}}({t})\right\rangle }_{{{\rm{ideal}}}}={e}^{(2(4{\gamma }_{z}+4{\gamma }_{-})+(2{\gamma }_{z}+\frac{1}{2}{\gamma }_{-})){t}}{{\rm{Tr}}}\left[({{\mathcal{M}}}\otimes {\sigma }_{x})W(t)\right]\,,$$

where $2{\gamma }_{z}+\frac{1}{2}{\gamma }_{-}$ in the exponential pre-factor corrects the ancilla noise, as previously discussed. In Fig. 2 we plot the noisy, ideal, and mitigated expectation values of the total magnetisation, for *ξ* = 0.2, *J* = 2, *h* = 0.1 and *γ*_*z*_ = *γ*_−_ = 0.03. The mitigated expectation values at each time step are estimated using 10^6^ samples. Notice that when ancilla noise is not accounted for in the mitigation scheme, the resulting multiplicative error grows exponentially with time, leading to a substantial discrepancy from the ideal expectation value.

#### Loschmidt echo

Next, we apply our protocol to retrieve characteristic features of quench dynamics that would otherwise be obscured by noise. A quantum quench refers to a non-equilibrium scenario where a system is initially prepared in the ground state $\left\vert {\psi }_{0}\right \rangle$ of an initial Hamiltonian *H*_0_, which is then suddenly changed—typically through a rapid variation of a tunable parameter—to a new Hamiltonian *H*, resulting in the following dynamics $\left\vert \psi (t)\right \rangle={e}^{-iHt}\left\vert {\psi }_{0}\right \rangle$. Following a quantum quench, the system may exhibit critical behaviour associated with a dynamical quantum phase transition (DQPT)^65^. Such transitions are accompanied by a non-analytic behaviour of the Loschmidt echo, defined as the overlap between the evolved state and the initial state, ${{\mathcal{L}}}(t)=\vert \left\langle \psi_{0} \vert \psi (t)\right\rangle \vert ^{2}$, as a function of time. Equivalently, this critical behaviour can be captured by its normalised rate function, $r(t)=-\frac{1}{N}\log {{\mathcal{L}}}(t)$, where *N* denotes the number of degrees of freedom of the system (e.g. number of qubits). We consider the paradigmatic example of a 1D transverse-field Ising model, described by the Hamiltonian

18 $$H=J{\sum }_{i=1}^{N}{\sigma }_{z}^{(i)}{\sigma }_{z}^{(i+1)}+h{\sum }_{i=1}^{N}{\sigma }_{x}^{(i)}\,,$$

with periodic boundary conditions and *N* denoting the number of spins in the chain.

We focus on the quantum quench where the system is prepared in $\left\vert {\psi }_{0}\right \rangle={\left\vert 0\right\rangle }^{\otimes N}$ (ground state of the Ising Hamiltonian in the absence of transverse field) and then evolves under the full Hamiltonian in Eq. 18. While this system is known to exhibit a DQPT strictly in the thermodynamic limit^66,67^, for finite spin chains the normalised rate function of the Loschmidt echo displays smooth peaks characteristic of quench dynamics, which serve as precursors to the DQPT as they sharpen into kinks with increasing system size. However, sufficient noise strongly suppresses these features. We model the dephasing noise affecting both the *N* spins and the ancilla qubit using the Lindblad operators $\sqrt{{\gamma }_{z}}{\sigma }_{z}$, leading to *a* = *Nγ*_*z*_. Since these operators are all unitary up to a constant factor, we apply the simplified protocol given by the mitigation Lindbladian in Eq. 9. The normalised rate of the noiseless Loschmidt echo can then be expressed as

19 $$r(t)=\dfrac{2(N+1)}{N}{\gamma }_{z}+\log {{\rm{Tr}}}\left[\left(\left\vert {\psi }_{0}\right\rangle \left\langle {\psi }_{0}\right\vert \otimes {\sigma }_{x}\right)W(t)\right],$$

where *W*(*t*) is the joint state of *N* + 1 spins. We note that the logarithm of the expectation value from the mitigation protocol differs from the noiseless case only by a constant shift, $\frac{2(N+1)}{N}{\gamma }_{z}$, thus retaining the typical signatures of a DQPT.

**Fig. 3:**
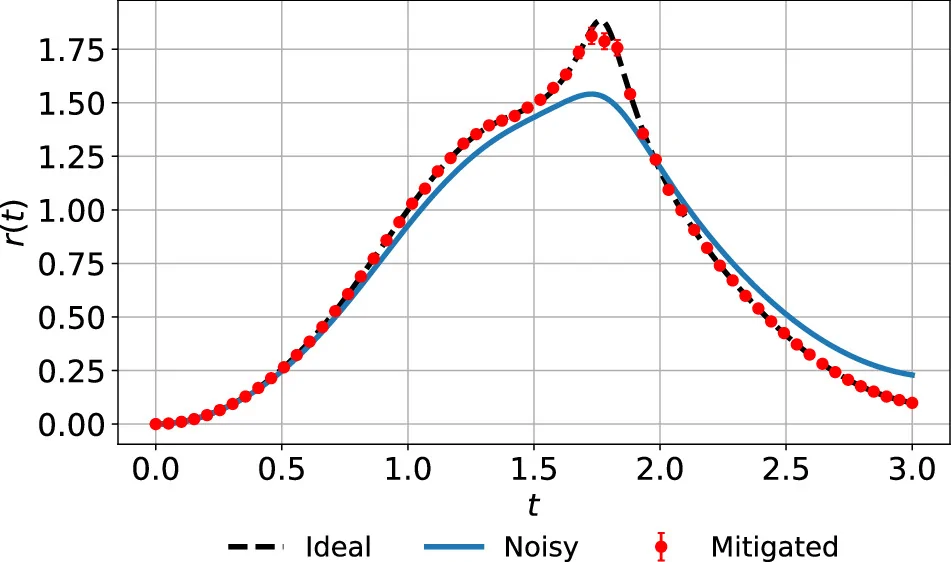
Normalised rate of the Loschmidt echo after quantum quench in a 1D spin Ising chain subject to the transverse field. The mitigated expectation values and their standard deviations (represented by the error bars) are obtained by averaging over 5 × 10^6^ samples.

In Fig. 3, we plot the ideal, noisy, and mitigated normalised rates of Loschmidt echoes for the Ising model described above, for *N* = 4, *J* = 0.2, *h* = 1 and *γ*_*z*_ = 0.1. The mitigated expectation values at each time step are estimated using 5 × 10^6^ samples. We observe that, while noise suppresses the peak, our mitigation protocol effectively recovers it.

#### Floquet dynamics

**Fig. 4:**
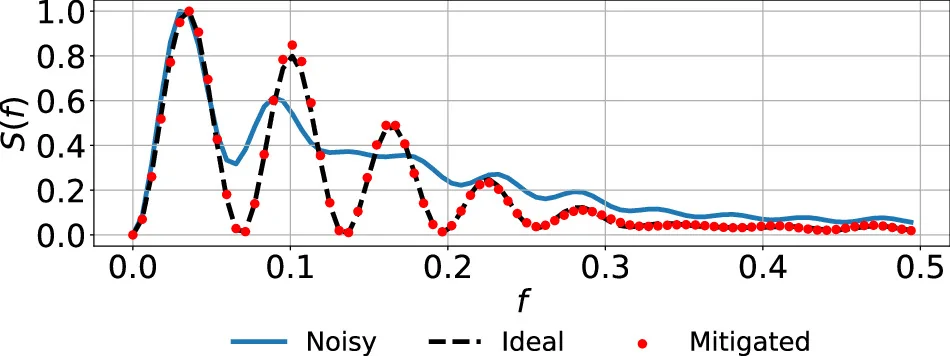
Normalised power spectrum of the total magnetisation for a periodically driven spin chain. The mitigated power spectrum is computed from expectation values obtained by averaging over 10^7^ samples.

Lastly, we apply our mitigation scheme to a periodically driven (Floquet) quantum system. Specifically, we consider a spin-1/2 chain subject to a binary drive that alternates between two non-commuting Hamiltonians,

20 $${H}_{1}=J{\sum }_{i=1}^{N-1}{\sigma }_{z}^{(i)}{\sigma }_{z}^{(i+1)}\,,{H}_{2}=h{\sum }_{i=1}^{N}{\sigma }_{x}^{(i)},$$

where *N* is the number of spins. Each Floquet period *T* consists of evolution under *H*_1_ for a time δ*t*, followed by evolution under *H*_2_ for the remaining duration *T* − δ*t*. The system is initialised in the state ${\left\vert 0\right\rangle }^{\otimes N}$, and we stroboscopically measure the expectation value of the total magnetisation ${{\mathcal{M}}}={\sum}_{i}{\sigma }_{z}^{(i)}$ at integer multiples of the driving period, *t* = *nT* with *n* = 0, 1,…,*N*_cycles_. Its discrete Fourier transform $\widetilde{{{\mathcal{M}}}}(f)$, is then used to construct the (normalised) power spectrum ${S}_{{{\mathcal{M}}}}(f)\propto | \widetilde{{{\mathcal{M}}}}(f){| }^{2}$. In the absence of noise, the normalised power spectrum exhibits peaks at frequencies corresponding to differences of Floquet quasi-energies, reflecting coherent interference between Floquet modes, providing a direct probe of the system’s underlying quasi-energy structure. When dephasing noise is introduced, coherences between Floquet eigenstates are progressively destroyed, leading to broadened or suppressed peaks. To model this effect, we consider each spin to be subject to independent dephasing noise, described by jump operators $\sqrt{\gamma }{\sigma }_{z}^{(i)}$. We then apply our mitigation scheme using the simplified Lindbladian in Eq. 9. As shown in Fig. 4, we compare the ideal, noisy, and mitigated power spectra of the total magnetisation for a spin chain with *N* = 6 spins, δ*t* = 0.5, *T* = 1, *J* = *h* = 1, *γ* = 0.025 and *N*_cycles_ = 20. The mitigated expectation values of the magnetisation used to compute the power spectrum are obtained by averaging over 10^7^ samples. While dephasing washes out most of the Floquet peaks, our mitigation protocol effectively restores them, thereby recovering the correct quasi-energy structure of the driven spin system.

## Discussion

In this work, we have introduced a fully autonomous time-continuous error mitigation protocol for analogue quantum simulators that enables the recovery of noiseless expectation values of arbitrary observables. The scheme couples the system to a small number of ancillary qubits through dissipative interactions, followed by classical post-processing of the joint measurement outcomes. In contrast to existing error-mitigation techniques that are inherently digital and therefore subject to Trotter errors and Hamiltonian-dependent artifacts^30,31,68^, our protocol operates directly within the framework of open-system Lindbladian dynamics^6^. As such, it is continuous in time and therefore exact for arbitrary system Hamiltonians and noise operators, avoiding both the superoperator Trotterisation errors^68^ and the hidden dependence of parameterised noise layers on the underlying noiseless gates^30^. Our result can also be compared with a Lindbladian-based error-suppression protocol applied to encoded qubits^69^, whereas our approach operates without encoding, requiring only a small number of qubits with fewer interactions, at the cost of additional sampling overhead. Moreover, we have demonstrated that the protocol remains robust under realistic ancilla noise models, which can be simply done by adjusting the post-processing factor.

Taken together, our results establish a framework for high-precision analogue quantum simulation, complement recent advances in error-mitigation strategies^70–72^, and offer new opportunities for exploring complex quantum dynamics resilient to decoherence. Our protocol could be directly applied to improve adiabatic quantum computing^73^ in the presence of moderate noise, and, more broadly, its ancilla-assisted noise-protection formalism could be utilised for a continuous-time analogue of fault-tolerant gate synthesis^74,75^.

## Methods

### Damped Schrödinger equation

Here, we explicitly derive Eq. 4 by projecting the Lindblad master equation for the joint system, Eq. 2, onto the computational basis of the ancilla qubit. Let *i*, *j* ∈ {0, 1}, with *i* ≠ *j*, and define ${W_{ij}}\equiv \left\langle i \right| W\left\vert j\right \rangle$. The contribution from the system’s noisy dynamics is then given by

21 $$\left\langle i \right \vert {{{\mathcal{L}}}}_{\exp }(W) \left\vert j \right\rangle=-i \left[H,{W_{ij}}\right]+{\sum}_{k}{{\mathcal{D}}}\left[{L}_{k}\right]({W_{ij}}) \\=-i \left[H,W_{ij}\right]+{\sum}_{k}\left({L}_{k}{W_{ij}}{L}_{k}^{{\dagger} }-\dfrac{1}{2}\{{L}_{k}^{{\dagger} }{L}_{k},{W_{ij}}\}\right)\,.$$

The mitigation Lindbladian, Eq. 3, consists of three distinct terms. We compute each contribution separately, starting with

22 $$\langle i| {\sum}_{k}{{\mathcal{D}}}[{L}_{k}\otimes {\sigma }_{z}](W)\vert j \rangle \\={\sum}_{k}\left(-{L}_{k}{W_{ij}}{L}_{k}^{{\dagger} }-\dfrac{1}{2}\{{L}_{k}^{{\dagger} }{L}_{k},{W_{ij}}\}\right)\,,$$

where we have used ${\sigma }_{z}^{{\dagger} }{\sigma }_{z}={\mathbb{I}}$. The remaining two contributions are given by

23 $$\left\langle i | {{\mathcal{D}}}[\sqrt{S}\otimes {\mathbb{I}}](W)\vert j\right\rangle=\sqrt{S}{W_{ij}}\sqrt{S}-\dfrac{1}{2} \left\{S,{W_{ij}}\right\}\,,$$

24 $$\left\langle i| {{\mathcal{D}}}\left[\sqrt{S}\otimes {\sigma }_{z}\right](W)| j\right\rangle=-\sqrt{S}{W_{ij}}\sqrt{S}-\dfrac{1}{2}\left\{S,{W_{ij}}\right\}\,.$$

Combining all contributions from ${{{\mathcal{L}}}}_{\exp }$ and ${{{\mathcal{L}}}}_{{{\rm{mit}}}}$, we finally obtain

25 $$\frac{d}{dt}{W_{ij}} 	=\left\langle i \left| {{{\mathcal{L}}}}_{\exp} (W)+{{{\mathcal{L}}}}_{{\rm{mit}}} (W) \right|\, j\right\rangle \\ 	=- i \left[H,{W_{ij}}\right] - \left\{{\sum}_{k} {L}_{k}^{{\dagger}} {L}_{k}+S,{W_{ij}}\right\} \\ 	=- i \left[H,{W_{ij}}\right] - \left\{a{\mathbb{I}},{W_{ij}}\right\} \\ 	=- i \left[H,{W_{ij}}\right] - 2a{W_{ij}}\,,$$

where we used the definition $S=a{\mathbb{I}}-{\sum}_{k}{L}_{k}^{{\dagger} }{L}_{k}$. That is, *W*_*ij*_ evolves according to a Schrödinger equation generated by *H*, with an additional damping term.

### Noiseless expectation value

Here, we show that the noiseless expectation value of a system observable *A* can be recovered from the measurement on the combined system-ancilla state, evolved under the Lindblad master equation in Eq. 2. We previously showed that if the joint initial state is $W(0)=\rho (0)\otimes \left\vert+\right\rangle \left\langle+\right\vert$, and we let it evolve under Eq. 2, then the off-diagonal block of the joint density matrix reads

26 $${W_{ij}}(t)=\dfrac{{e}^{-2at}}{2}{e}^{-iHt}\rho (0){e}^{iHt}\,.$$

This shows that *W*_*ij*_(*t*) is proportional to the system’s density matrix evolved under ideal unitary dynamics generated by the Hamiltonian. To recover the noiseless expectation value of *A*, we use the fact that *W*_01_(*t*) = *W*_10_(*t*), which follows from the symmetric initialisation *W*_01_(0) = *W*_10_(0) and that fact that both operators satisfy the same differential equation, Eq. 5. The ideal expectation value of *A* at time *t* can then be written as

27 $${\langle A(t) \rangle }_{{\rm{ideal}}} 	={{\rm{Tr}}}_{{\rm{sys}}} \left[A{e}^{ - iHt} \rho (0) {e}^{iHt}\right] \\ 	=2{e}^{2 at} {{\rm{Tr}}}_{{\rm{sys}}} \left[A W_{01} (t)\right] \\ 	={e}^{2 at} {{\rm{Tr}}}_{{\rm{sys}}} \left[A (W_{01}(t)+{W_{10}}(t))\right] \\ 	={e}^{2 at} {{\rm{Tr}}}_{{\rm{sys}}} \left[A {{\rm{Tr}}}_{{\rm{anc}}} \left[(| 0\rangle \langle 1 |+| 1 \rangle \langle 0 | ) W (t)\right]\right] \\ 	={e}^{2 at} {{\rm{Tr}}} \left[(A \otimes {\sigma}_{x}) W(t)\right],$$

where we have used ${\sigma }_{x}=\left\vert 0\right\rangle \left\langle 1 \right\vert+\left\vert 1\right\rangle \left\langle 0\right\vert$, concluding our proof.

### Reduced sampling overhead using multi-flag registers

A naive application of the protocol incurs sampling overhead that scales exponentially with system size *N*, i.e. ${a}_{N}={\sum }_{i=1}^{N}{a}_{i}=N{a}_{1}$, assuming that each subsystem has the same rescaling factor *a*_1_ = *a*_2_ = ⋯ = *a*_*N*_. To overcome this problem, we develop a more efficient protocol using a multi-flag ancilla register, which significantly reduces the sample complexity from exponential scaling to an observable-specific, weight-resolved overhead.

**Fig. 5:**
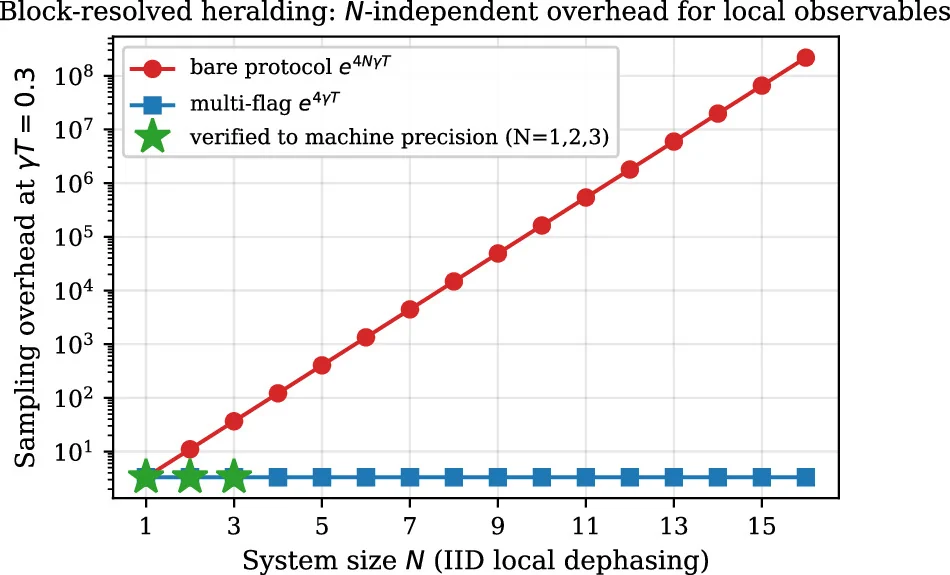
Sampling-overhead reduction of the multi-flag construction for IID local dephasing ${{\mathcal{D}}}[\sqrt{\gamma }{\sigma }_{z}^{(\ell)}]$ on *N*-qubit chains with target observable ${\sigma }_{x}^{(1)}$ for the first qubit. The bare-protocol overhead *e*^4*NγT*^ grows exponentially in *N*; the multi-flag overhead *e*^4*γT*^ is flat. Star markers are direct numerical verifications at machine precision.

The main idea is to apply recovery dynamics using selected ancillary registers to cancel only the components of the noisy dynamics that effectively act on the target observables in the Heisenberg picture. As an intuitive example, consider a noninteracting Hamiltonian and a macroscopic linear observable, such as the total magnetisation ${{\mathcal{M}}}=\mathop{\sum }_{\ell=1}^{N}{\sigma }_{z}^{(\ell )}$. In this case, the observable on the *ℓ*th qubit is affected only by its local noise and not by noise acting on the other qubits. By using independent ancilla qubits for the individual noise channels to flag the noise acting on each qubit, the protocol to estimate each local observable ${\sigma }_{z}^{(\ell )}$ achieves a sampling overhead of ${e}^{4{a}_{1}t}$ rather than ${e}^{4N{a}_{1}t}$ (see Fig. 5). Thus, the exponential factor in the sampling overhead is independent of the system size, with only a linear prefactor in *N*. This construction explicitly saturates the universal weight-resolved bound^37,76^.

For a general observable *A* under a locally interacting Hamiltonian, the overhead scales as ${e}^{4{a}_{1}| C(A,t)| t}$, where ∣*C*(*A*, *t*)∣ is the size of the Heisenberg light cone defining the effective region of the dynamics associated with *A*, which is determined by the Lieb–Robinson bound. More generally, we can decompose the noisy Lindbladian into Schur-irreducible blocks as ${L}_{k}{=\bigoplus }_{\mu }{L}_{k}^{(\mu )}$ with ${\lambda }_{\mu }={\left\Vert \mathop{\sum}_{k}{L}_{k}^{(\mu ){\dagger} }{L}_{k}^{(\mu )}\right\Vert }_{\infty }$. The recovered variance of an observable supported on block *μ* then scales as ${e}^{4{\lambda }_{\mu }t}$, instead of ${e}^{4{a}_{N}t}$ by utilising the ancilla flagging the block corresponding to *μ* effectively acting on the target observable. We refer the reader to Supplementary Note 3 for the explicit derivation and more detailed discussion.

### Robustness against system-ancilla correlated errors

The mitigation protocol can also handle certain forms of correlated noise between the system and the ancilla. This is particularly relevant in the presence of crosstalk, where unintended residual couplings or control leakage can give rise to correlated errors across the system-ancilla degrees of freedom.

For instance, let us consider a Lindbladian noise term ${{\mathcal{D}}}[P\otimes {\sigma }_{\pm }]$, where *P* is a generic Pauli string acting on the system qubits. A straightforward calculation shows that

28 $$\left\langle i\left| {{\mathcal{D}}}[P\otimes {\sigma }_{\pm }](W)\right| j\right\rangle=-\dfrac{W_{ij}}{2},$$

implying that the effect of this noise can be cancelled out via classical post-processing, as shown in the main text. If we instead consider ${{\mathcal{D}}}[P\otimes {\sigma }_{z}]$ as model of correlated noise, we observe that

29 $$\left\langle i \right| {{\mathcal{D}}}[P\otimes {\sigma }_{z}](W) \left\vert j\right\rangle=-P{W_{ij}}P-{W_{ij}}.$$

Compared to the previous case, this is not proportional to *W*_*ij*_, implying that mitigation via our scheme is not possible by simple post-processing in this scenario. Nevertheless, by introducing additional system noise via ${{\mathcal{D}}}[P\otimes {\mathbb{I}}]$, which contributes with $\left\langle i \right| {{\mathcal{D}}}[P\otimes {\mathbb{I}}](W)\left\vert j\right\rangle=P{W_{ij}}P-{W_{ij}}$ to the master equation governing *W*_*ij*_, the combined effect is now proportional to *W*_*ij*_ and reads −2*W*_*ij*_. In particular, when *P* is the noise jump operator, the effect of the correlated noise is exactly the same as that of the mitigation dynamics itself, so it can be absorbed as a miscalibration of the noise rate.

This shows that even this form of correlated noise can be mitigated with an appropriate modification of the scheme. A comprehensive analysis of the protocol’s robustness to ancilla noise, beyond the specific examples considered here, is left for future work. More detailed analysis of the effect of crosstalk and correlated errors can be found in Supplementary Note 6.

### Robustness against coherent ancilla errors

We discuss the protocols’ robustness against coherent ancilla errors, which may rotate the encoded off-diagonal block that carries the ideal state’s information. This situation can be generally modelled by the misalignment between the ancilla state and the final measurement axis *σ*_*x*_ in the Heisenberg picture, as

30 $$\widetilde{\sigma }({\rm\delta })={R}^{{\dagger} }({\rm\delta }){\sigma }_{x}R({\rm\delta })=({\mathrm{cos\delta }}){\sigma }_{x}+({\mathrm{sin\delta }}){\sigma }_{\hat{n}},$$

where *R*^†^(δ) is the rotation unitary of the ancilla state with the misalignment angle δ, and $\hat{n}$ is a vector orthogonal to the *x*-axis, i.e. ${{\rm{Tr}}}[{\sigma }_{x}{\sigma }_{\hat{n}}]=0$.

In this case, the misaligned observable at the final measurement becomes $A\otimes \tilde{\sigma }(\delta )$ instead of *A* ⊗ *σ*_*x*_. Remarkably, we show that such coherent errors are efficiently suppressed by the symmetry without any further actions with error with leading order ${ \mathcal O }({{\delta }}^{2})$. To this end, we note that the system ancilla joint state after the mitigation dynamics can be expressed as

31 $$W(t) 	=\left(\begin{array}{c}{W}_{00}(t)\quad{W}_{01}(t) \\ {W}_{10}(t)\quad{W}_{11}(t)\end{array}\right) \\ 	=\dfrac{1}{2}\left(\begin{array}{c}\tilde{\rho }(t)\quad{e}^{-2at}{\rho }_{\mathrm{ideal}}(t)\\ {e}^{-2at}{\rho }_{\mathrm{ideal}}(t)\quad\tilde{\rho }(t)\end{array}\right) \\ 	=\dfrac{1}{2}\left[\tilde{\rho }(t)\otimes {\mathbb{I}}+{e}^{-2at}{\rho }_{\mathrm{ideal}}(t)\otimes {\sigma }_{x}\right],$$

by noting that *W*_00, 01, 10, 11_(0) = *ρ*(0)/2 and using Eqs. 4, 5 and 6. Here, *ρ*_ideal_(*t*) = *e*^−*iHt*^*ρ*(0)*e*^*iHt*^ is the noiseless system state after time *t* and $\tilde{\rho} (t)$ is the time-evolve state under Eq. 5 with *W*_*ii*_ = *ρ*(0). We then obtain

32 $${\mathrm{Tr}} \left[(A\otimes \widetilde{\sigma }({\rm\delta }))W(t)\right]=({\mathrm{cos\delta }}){e}^{-2at}{\langle A(t)\rangle }_{{\mathrm{ideal}}},$$

where we only have the additional factor ${\mathrm{cos\delta }}$ compared to the coherent-error-free case, and $\tilde{\rho} (t)$ does not contribute. For δ ≪ 1, we have ${\mathrm{cos\delta }}\approx 1-{{\rm\delta }}^{2}/2$, so that the leading order of correction becomes ${{\mathcal{O}}}({\delta }^{2})$ with fully suppressed first-order contribution. Such a coherent ancilla error suppression can be further combined with a more delicate ancilla-register design using error-correction codes, with more details in Supplementary Note 5.

### Non-Markovianity effects

The exact cancellation result assumes that the engineered system-ancilla dynamics is described by a time-local Lindblad generator over the mitigation window. This assumption can be relaxed to time-dependent Markovian rates: if the jump operators are fixed but the rates drift slowly or are calibrated as a function of time, the proof is unchanged after replacing *at* by $\bar{a}(t)=\int_{0}^{t}a(s)ds$. Thus, stationarity of the rates is not essential, although the relevant time-dependent rates must be known or calibrated.

In more realistic situations, however, deviations from a time-local Markovian description can arise from system–reservoir correlations^77–79^ or from additional noise tailoring or noise-model processing, for example, through Pauli twirling^80^. Such effects should be regarded as residual model mismatch relative to the ideal Lindblad description assumed in the exact cancellation proof.

If the bath correlation time is *τ*_*B*_, the memoryless Poisson description of the engineered jumps is only approximate. In the regime *aτ*_*B*_ ≪ 1, the induced correction to the effective time-local description is perturbative, and the resulting bias is controlled by the small parameter *aτ*_*B*_, accumulated over the evolution time. When *aτ*_*B*_ ≳ 1, the bath memory can interfere with the engineered heralding mechanism, and the exact Lindblad cancellation is no longer guaranteed. This defines a practical applicability criterion for the protocol.

### Comparison between existing continuous-time QEM approaches

In stochastic impulsive recovery protocols such as ref. ^30^, recovery is implemented by finite-time operations

33 $${{{\mathcal{B}}}}_{j}=\exp (-i{H}_{{{\rm{kick}}}}{\tau }_{{{\rm{kick}}}})$$

at pre-sampled jump times. The ideal continuum limit requires a fixed recovery rotation to be applied in the sharp-pulse limit *τ*_kick_ → 0, and hence requires ∥*H*_kick_∥ → *∞*. With a bounded physical drive $\parallel {H}_{{{\rm{drive}}}}\parallel \le {H}_{\max }$, a rotation by angle *θ*_*j*_ requires ${\tau }_{{{\rm{kick}}}}\gtrsim {\theta }_{j}/{H}_{\max }$. During this finite pulse window, the background simulator Hamiltonian continues to act, producing a residual finite-bandwidth error. This is consistent with ref. ^30^, which identifies a per-pulse residual of order ${ \mathcal O }(({\delta }{t})^{3})$ and requires the kick intensity to be sufficiently large compared with the simulator Hamiltonian.

Our protocol avoids this particular tradeoff because the mitigation is generated by a bounded-norm, time-independent engineered Lindbladian rather than by instantaneous trajectory-dependent recovery pulses. For a noise jump operator *L*_*k*_, the corresponding mitigation jump is *L*_*k*_ ⊗ *σ*_*z*_ with ∥*L*_*k*_ ⊗ *σ*_*z*_∥ = ∥*L*_*k*_∥. Thus, the engineered dissipation is required only at the scale of the noise channel being mitigated, not as a parametrically stronger instantaneous drive. In this sense, our construction is complementary to the pre-engineered Hamiltonian variant of ref. ^30^, while realising the mitigation dynamics in an autonomous continuous-time form.

In influence-martingale protocols such as ref. ^31^, the mitigation factor is a trajectory-dependent classical weight *μ*[*ω*] ﻿, and therefore requires the quantum-jump record *ω* to be monitored and post-processed on a shot-by-shot basis. The ancilla-based construction in ref. ^31^ changes how the trajectories are generated, but the click record is still measured and used to construct *μ*[*ω*].

Our approach can be distinguished from this as we engineer a signed-erasure structure in which each system jump is accompanied by *L*_*k*_ ⊗ *σ*_*z*_. As a result, the trajectory-dependent sign reduces to an ancilla parity, which can be read out by a single terminal measurement of *σ*_*x*_. In this case, the usual QEM sampling cost is not removed, but appears as the global variance amplification *e*^4*at*^. We refer to Supplementary Note 7 for a more detailed comparison between our approach refs. ^30,31^.

## Supplementary information

Supplementary Information

Transparent Peer Review File

## Data Availability

All the data and codes used in this study are available from Zenodo https://doi.org/10.5281/zenodo.22202304.
