# Supplementary material for: Continuous-time noise mitigation in analogue quantum simulation
Source: Nat Commun. 2026 Oct 12;17:11557. doi: 10.1038/s41467-026-78310-5 (PMC13748635; doi:10.1038/s41467-026-78310-5)
Supplement: Supplementary file 1 [file 41467_2026_78310_MOESM1_ESM.pdf]

Supplementary Information: Continuous-time noise mitigation in analogue quantum simulation

Gabriele Bressanini,[1] Yue Ma,[1] Michael Hanks,[1] Hyukjoon Kwon,[2,*] and M. S. Kim[1,2,†]

[1] *Blackett Laboratory, Imperial College London, London SW7 2AZ, United Kingdom*
[2] *Korea Institute for Advanced Study, Seoul 02455, South Korea*

CONTENTS

I. Supplementary Note 1: Common notation 2

II. Supplementary Note 2: Alternative Protocols 3
A. Qubit ancilla 3
B. Qutrit ancilla 4

III. Supplementary Note 3: Block-resolved heralding (multi-flag construction) 5
A. Setting 5
B. Construction 5
C. Block-resolved overhead 5
D. Sampling overhead reduction for local observables 6
E. Physical realisability of the multi-flag construction 6

IV. Supplementary Note 4: Explicit correspondence with an erasure channel 8
A. Setting: dephasing the classical flag 8
B. Erasure: the $\sigma_x^{(a)}$ projector kills collapsed branches 8
C. Equivalence with explicit $\sigma_x^{(a)}$-measurement 9
D. Generalisation to block-resolved heralding 9

V. Supplementary Note 5: Coherent ancilla miscalibration and encoded-ancilla checks 10
A. Bare-ancilla first-order robustness 10
B. Distance-2 ancilla error detection protocol 10
C. Numerical demonstration 10
D. Leakage out of the ancilla subspace 11
E. Connection to erasure-converted qubits 11

VI. Supplementary Note 6: Further considerations on other realistic noise models 12
A. Imperfect noise characterization and engineered-rate errors 12
B. Ancilla dephasing and local ancilla noise 12
C. System–ancilla crosstalk and correlated noise 12
D. Combined realistic-noise simulation 12

VII. Supplementary Note 7: Comparison with existing continuous-time QEM protocols 14
A. Pulse-bandwidth constraint in impulsive recovery protocols 14
B. Influence-martingale protocols and jump-record cost 14
C. Ensemble-averaged stochastic implementation versus trajectory-conditioned stochasticity 15
D. Numerical comparison with Monte-Carlo unravelling 15
E. Structural feasibility comparison under implementation constraints 16

References 17

* hkwon@kias.re.kr
† m.kim@imperial.ac.uk

I. SUPPLEMENTARY NOTE 1: COMMON NOTATION

For clarity, the following table summarizes the notation used throughout this Supplementary Information.

Symbol or term	Meaning
Basic dynamical quantities	
$\mathcal{D}[L](\rho)$	Lindblad dissipator, $\mathcal{D}[L](\rho) = L\rho L^\dagger - \frac{1}{2}\{L^\dagger L, \rho\}$.
$W(t)$	Joint system–ancilla density matrix.
H or H_S	System Hamiltonian. The mitigation Lindbladian is independent of the detailed form of H_S, except through the usual Heisenberg spreading of the measured observable.
L_k	System jump operators. When rates are written separately, L_k denotes the normalized jump operator and γ_k its rate; otherwise $\sqrt{\gamma_k}$ is absorbed into L_k.
a and a_S	Spectral shift controlling the global rescaling. In the single-flag convention of the SI, $a = \lambda_{\max}(\sum_k L_k^\dagger L_k)$; a_S denotes the corresponding system noise rate in the comparison and multi-flag sections.
$\bar{a}(t)$	Integrated time-dependent rate, $\bar{a}(t) = \int_0^t a(s)\,ds$, used when the dynamics remains time-local and Markovian but the rates vary in time.
S	Regularizer used to make the no-jump attenuation scalar, e.g. $S = a\mathbb{I} - \sum_k L_k^\dagger L_k$ or its local analogue $S^{(\ell)}$.
Multi-flag and block-resolved notation	
G	Finite symmetry or noise-commutant group used to organize the multi-flag construction.
μ	Irrep label of G in the multi-flag construction. The same letter is used later for irrep charges in the selection-rule theorem; the two uses are distinguished in the surrounding text.
Π_μ	Isotypic projector onto the μ sector of the system Hilbert space.
$L_k^{(\mu)}$	Irrep-block component $L_k^{(\mu)} = \Pi_\mu L_k \Pi_\mu$ of a system jump operator.
λ_μ	Per-irrep noise rate, $\lambda_\mu = \lVert \sum_k L_k^{(\mu)\dagger} L_k^{(\mu)} \rVert_\infty$.
λ_1	Single-site noise rate in IID local-noise examples.
μ_A	Irrep block, or selected collection of local blocks, relevant to the recovered observable A_{μ_A}. When several local blocks are measured, λ_{μ_A} denotes the sum of their rates.
$C(A_S, T)$	Heisenberg lightcone of the system observable A_S over time T, used when comparing local-observable overheads.
$\sigma_z^{(\mu)}$, $\sigma_x^{(\mu)}$	Pauli operators on the flag qubit associated with irrep label μ.
A_{rec}	Recovered joint observable. In the single-flag protocol $A_{\text{rec}} = A_S \otimes \sigma_x^{(a)}$; in the multi-flag construction it contains the product of the relevant $\sigma_x^{(\mu)}$ flag operators.

II. SUPPLEMENTARY NOTE 2: ALTERNATIVE PROTOCOLS

The continuous-time error mitigation scheme presented in the main text can be readily adapted to suit different experimental needs. Here, we introduce several alternative protocols that demonstrate this flexibility. These include variants that: (i) retain a single qubit ancilla, but use different correlated noise dissipators; (ii) employ multiple ancilla qubits; or (iii) employ a multi-level ancilla. All protocols share the same underlying noisy system dynamics, generated by the Lindbladian

$$\mathcal{L}_{\text{exp}}(W) = -i[H \otimes \mathbb{I}, W] + \sum_k \mathcal{D}[L_k \otimes \mathbb{I}](W)\,, \tag{1}$$

which acts trivially on the ancilla. Hence, in the following we only specify the corresponding mitigation Lindbladian $\mathcal{L}_{\text{mit}}(W)$, where $W(t)$ is the joint system-ancilla density matrix.

A. Qubit ancilla

An alternative mitigation scheme that uses a single qubit ancilla is defined by

$$\mathcal{L}_{\text{mit}} = \mathcal{D}[\sqrt{2(a\mathbb{I} - \sum_k L_k^\dagger L_k)} \otimes |0\rangle\langle 0|] + \mathcal{D}[\sqrt{2(a\mathbb{I} - \sum_k L_k^\dagger L_k)} \otimes |1\rangle\langle 1|] + \sum_k \mathcal{D}[L_k \otimes \sigma_z]\,, \tag{2}$$

where a is chosen as the largest eigenvalue of $\sum_k L_k^\dagger L_k$, $a = \lambda_{\text{max}}(\sum_k L_k^\dagger L_k)$. We initialize the joint system-ancilla state as $W(0) = \rho(0) \otimes |+\rangle\langle +|$, and the noiseless expectation value of a system observable A is recovered via

$$\langle A(t)\rangle = e^{2at}\,\mathrm{Tr}[(A \otimes \sigma_x)W(t)]\,, \tag{3}$$

resulting in the same sampling overhead found in the main text.

The protocol is also flexible when it comes to the number of ancilla qubits it employs. In fact, although we have shown that our protocol has the desirable property of only requiring one qubit regardless of the system's dimension, certain experimental scenarios may benefit from employing multiple ancilla qubits instead. For instance, in quantum simulators with limited connectivity, it may be difficult to implement the required correlated-noise dissipators between a single ancilla qubit and all system qubits. In such cases, our protocol can be naturally extended to accommodate multiple ancilla qubits. As an illustrative case, let us consider a setting where the $\ell-$th system qubit is subject to local noise characterized by the Lindblad operators $\{L_k^{(\ell)}\}$. Furthermore, we assume that each system qubit is paired with its own dedicated ancilla qubit it can interact with (intermediate configurations are easily derived). In this setup, the mitigation Lindbladian becomes:

$$\mathcal{L}_{\text{mit}} = \sum_\ell \left(\mathcal{D}[\sqrt{S^{(\ell)}} \otimes \sigma_z^{(\ell)}] + \mathcal{D}[\sqrt{S^{(\ell)}} \otimes \mathbb{I}] + \sum_k \mathcal{D}[L_k^{(\ell)} \otimes \sigma_z^{(\ell)}] \right)\,, \tag{4}$$

where $S^{(\ell)} = a^{(\ell)}\mathbb{I} - \sum_k L_k^{(\ell)\dagger} L_k^{(\ell)}$, and the superscript (ℓ) labels each system-ancilla qubit qubit pair. Similar to the previous protocols, we set $a^{(\ell)} = \lambda_{\text{max}}(\sum_k L_k^{(\ell)\dagger} L_k^{(\ell)})$ and initialize the joint system-ancilla state as $W(0) = \rho(0) \otimes |+\rangle\langle +|^{\otimes n}$, where n denotes the number of system qubits, and therefore that of ancilla qubits. This symmetric initialization ensures that all 2^n off-diagonal blocks of the joint density matrix W, relative to the computational basis of the ancilla qubits, coincide at all times. As a result, the noiseless expectation value of a system observable A can be recovered by jointly measuring A on the system qubits, and σ_x on each ancilla qubit

$$\langle A(t)\rangle = e^{2\sum_\ell a^{(\ell)} t}\,\mathrm{Tr}\big[(A \otimes \sigma_x^{\otimes n})W(t)\big]\,, \tag{5}$$

from which it follows that the sampling overhead scales as $e^{4\sum_\ell a^{(\ell)} t}$. A natural question is how the multi-ancilla protocol's sampling overhead compares to that of the single-ancilla scheme. This reduces to comparing $a = \lambda_{\text{max}}(\sum_{k\ell} L_k^{(\ell)\dagger} L_k^{(\ell)})$ with $\sum_\ell a^{(\ell)} = \sum_\ell \lambda_{\text{max}}(\sum_k L_k^{(\ell)\dagger} L_k^{(\ell)})$. In general, the largest eigenvalue of a sum of positive semi-definite operators is less than or equal to the sum of their individual largest eigenvalues. Here, however, the equality trivially holds because of the locality of the noise operators. Hence, we conclude that the two protocols incur the same sampling overhead. This changes, naturally, if the ancilla qubits are themselves noisy. In this case, the total noise to be cancelled effectively doubles, compared to the single-ancilla protocol, resulting in a quadratic increase of the sampling overhead.

B. Qutrit ancilla

The error cancellation protocol can also be adapted to use a qutrit ancilla. Consider a three-level ancilla, whose Hilbert space is spanned by $\{|1\rangle, |2\rangle, |3\rangle\}$. A possible mitigation Lindbladian in this setting is given by

$$\mathcal{L}_{\text{mit}} = \mathcal{D}[\sqrt{2(a\mathbb{I} - \sum_i L_i^\dagger L_i)} \otimes |3\rangle\langle 1|] + \mathcal{D}[\sqrt{2(a\mathbb{I} - \sum_i L_i^\dagger L_i)} \otimes |3\rangle\langle 2|] + \sum_i \mathcal{D}[L_i \otimes \tilde{\sigma}_z] \,, \tag{6}$$

where $\tilde{\sigma}_z = |1\rangle\langle 1| - |2\rangle\langle 2|$. The joint system-ancilla state is initialized as $W(0) = \rho(0) \otimes |\chi\rangle\langle\chi|$, with $|\chi\rangle = (|1\rangle + |2\rangle)/\sqrt{2}$. Finally, by following the same derivation steps used in the ancilla qubit case—now focusing on the off-diagonal blocks $\langle i| W |j\rangle \equiv W_{ij}$, with $i \neq j$ and $i, j \in \{1, 2\}$, which encode the information about the noiseless system dynamics—we find that the noiseless expectation value of A can again be expressed as

$$\langle A(t)\rangle = e^{2at}\, \mathrm{Tr}[(A \otimes \tilde{\sigma}_x) W(t)] \,, \tag{7}$$

where $\tilde{\sigma}_x = |1\rangle\langle 2| + |2\rangle\langle 1|$. Alternative mitigation Lindbladians to Eq. (6) can easily be constructed by inspection. For instance, the operator $|3\rangle\langle 1|$ may be replaced with any $|\psi\rangle\langle 1|$ such that $\langle\psi|1\rangle = 0$ and/or $\langle\psi|2\rangle = 0$; similarly $|3\rangle\langle 2|$ may be replaced with any operator $|\phi\rangle\langle 2|$ satisfying $\langle\phi|2\rangle = 0$ and/or $\langle\phi|1\rangle = 0$.

Similarly to the qubit ancilla protocol discussed in the main text, this qutrit ancilla protocol also exhibits robustness under certain types of ancilla noise. For simplicity, we model the ancilla noise using a single Lindblad noise operator M. As a result, the master equation governing the evolution of the joint system-ancilla state becomes

$$\frac{d}{dt} W(t) = \mathcal{L}_{\text{exp}}(W) + \mathcal{L}_{\text{mit}}(W) + \mathcal{L}_{\text{anc}}(W) \,, \tag{8}$$

where

$$\mathcal{L}_{\text{anc}}(W) = \mathcal{D}[\mathbb{I} \otimes M](W) \,. \tag{9}$$

Consequently, the differential equation governing the evolution of W_{ij} now includes the additional term $\langle i| \mathcal{D}[\mathbb{I} \otimes M](W) |j\rangle$. The form of the operator M largely depends on the specific physical implementation of the three-level systems and, in particular, on the energy level arrangement and transition dynamics between the three states. In general, we can expect that typical ancilla noise processes include pure dephasing terms of the form $|i\rangle\langle i|$, as well as excitation loss or heating terms such as $|i\rangle\langle j|$, with $i \neq j$. By substituting these noise operators into Eq. (9) and computing their contributions explicitly, we find that only two types of behaviour arise. In some cases—specifically for the operators $\{|1\rangle\langle 3|, |2\rangle\langle 3|, \text{and} |3\rangle\langle 3|\}$— the contribution vanishes. In all other cases, i.e., for the remaining six operators, the noise introduces a non-zero contribution that reads $-W_{ij}/2$. As shown in the qubit-ancilla case, these noise contributions do not affect the correlated-noise dissipators within $\mathcal{L}_{\text{mit}}$. Instead, they simply modify the exponential pre-factor appearing in Eq. (7). In other words, these ancilla noise effects can be accounted for through a classical post-processing, namely an appropriate redefinition of $e^{2at} \mapsto e^{2\tilde{a}t}$, which only affects the sampling overhead of the protocol without altering its underlying structure.

III. SUPPLEMENTARY NOTE 3: BLOCK-RESOLVED HERALDING (MULTI-FLAG CONSTRUCTION)

In this section, we present a general formalism for the multi-flag construction and an explicit protocol, we termed block-resolved heralding, for reducing the sampling overhead.

A. Setting

Let us first decompose the system noise commutant under a finite group $G \subseteq \mathcal{U}(\mathcal{H}_S)$ that commutes with every system jump operator $[L_k, U(g)] = 0 \;\forall k, g$. Let $G = \{1, \mu_1, \ldots, \mu_r\}$ be the irreps of G, with isotypic projectors Π_μ on $\mathcal{H}_S$. Each L_k is irrep-block-diagonal: $L_k = \bigoplus_\mu L_k^{(\mu)}$ with $L_k^{(\mu)} = \Pi_\mu L_k \Pi_\mu$. Define the per-irrep noise rate

$$\lambda_\mu := \big\| \textstyle\sum_k L_k^{(\mu)\dagger} L_k^{(\mu)} \big\|_\infty, \qquad \lambda_{\mathrm{tot}} = \sup_\mu \lambda_\mu \le a_S. \tag{10}$$

The bare protocol uses a *single* ancilla flag and produces sampling overhead $e^{4a_S T}$ regardless of which irrep block of the noise is active. The block-resolved construction replaces this single flag with a *register of* $|G|$ *flag qubits*, one per irrep, herald-coupled so that an irrep-μ jump flips only the μ-flag.

Scope of the gain. We highlight that the advantage developed below is *observable-side*: the multi-flag construction trades the global overhead $e^{4a_S T}$ for a per-block overhead $e^{2\lambda_{\mu_A} T}$ *when the recovered observable* A_{μ_A} *is supported in (or concentrated on) a known irrep block* μ_A. For an observable with generic support across all irreps, the post-selection over the full flag register collapses the gain back to the bare-protocol scaling. The structural benefit is therefore a property of the *observable's* irrep support, not of the noise model alone, and is largest when the observable is local or symmetry-resolved.

B. Construction

Let us introduce ancilla flags $\{\sigma_z^{(\mu)}\}_{\mu \in G}$, each initialised in $|+\rangle$. The engineered Lindbladian then becomes

$$\tilde{L}_k^{(\mu)} = L_k^{(\mu)} \otimes \sigma_z^{(\mu)}, \qquad \tilde{\mathcal{L}}[\rho] = \sum_{k,\mu} \mathcal{D}[\tilde{L}_k^{(\mu)}](\rho). \tag{11}$$

The recovered observable for an observable A_{μ_A} supported in irrep block μ_A is

$$A_{\mathrm{rec}} = A_{\mu_A} \otimes \prod_\mu \sigma_x^{(\mu)} \xrightarrow{\text{post-selection}} A_{\mu_A} \otimes \sigma_x^{(\mu_A)} \quad \text{(conditional on all other flags reading } +1). \tag{12}$$

The conditional rescaling factor is no longer $e^{2a_S T}$, but rather $e^{2\lambda_{\mu_A} T}$, as we will show in the following.

C. Block-resolved overhead

Here, we state the main theorem and its proof.

Theorem (block-resolved overhead). *For an observable* $A_{\mu_A} \otimes \sigma_x^{(\mu_A)}$ *supported in irrep block* μ_A*, conditioned on every flag* $\mu \neq \mu_A$ *reading* $+1$*, the post-selected estimator is unbiased for the noiseless expectation value, with sampling overhead*

$$\mathcal{N}_{\mu_A}(T) = e^{4\lambda_{\mu_A} T} \quad \text{(per-shot squared weight).} \tag{13}$$

Proof. We note that the joint dynamics factorises across irreps. For each irrep μ, the engineered dissipator $L_k^{(\mu)} \otimes \sigma_z^{(\mu)}$ acts as an erasure channel that flags an irrep-μ jump on its own ancilla (see also Sec. IV). On the unfactorised joint state, define the parity operator $P^{(\mu)} = \sigma_x^{(\mu)}$, then the G-block-diagonal piece of the system-density matrix at time T is reconstructed from

$$\langle A_{\mu_A} \rangle_{\rho_S^{\mathrm{ideal}}(T)} = e^{2\lambda_{\mu_A} T} \big\langle A_{\mu_A} \otimes \sigma_x^{(\mu_A)} \big\rangle_{\rho_{SA}(T)} \Big|_{\text{other flags}=+1}. \tag{14}$$

The exponent $2\lambda_{\mu_A}$ (rather than $2a_S$) arises because only $L^{(\mu_A)\dagger} L^{(\mu_A)}$ contributes to the parity-conditioned trace–jumps in other irreps flip their own flags, which the post-selection has already projected out. The estimator's per-shot squared weight is therefore $e^{4\lambda_{\mu_A} T}$, giving the stated sample-complexity scaling. □

D. Sampling overhead reduction for local observables

As an example, we consider sampling overhead reduction for the local observables under IID single-qubit noise from $e^{4a_N T} = e^{4N\lambda_1 T}$ to $e^{4\lambda_{\mu_A} T}$, which becomes independent of the system size N. The reduction of the exponents in sampling overhead can be summarized as

$$\underbrace{e^{4N\lambda_1 T}}_{\text{[30] monolithic}} \xrightarrow{\text{(a) Heisenberg lightcone}} e^{4\lambda_1 |C(A_S,T)| T} \xrightarrow{\text{(b) Schur-block selection}} e^{4\lambda_{\mu_A} T}$$

with three operationally distinct steps. Step (a) is a property of the observable: A_S is sensitive only to noise inside its Heisenberg-evolved support $e^{iH_S T} A_S e^{-iH_S T}$, contained in a Lieb–Robinson lightcone of volume $|C(A_S, T)| = O((vT)^D)$ for the spatial dimension D at fixed T. Step (b) applies a Schur block μ_A of the noise commutant in which the recovered observable is supported: in-cone volume $\lambda_1 |C(A_S, T)|$ is replaced by the per-block spectral-norm rate $\lambda_{\mu_A} = \| \sum_k L_k^{(\mu_A)\dagger} L_k^{(\mu_A)} \|_\infty$, which for a Pauli-weight-w observable under IID dephasing is $\lambda_{\mu_A} = w\lambda_1$; the resulting overhead $e^{4w\lambda_1 T}$ is independent of N.

E. Physical realisability of the multi-flag construction

A natural concern is whether "one ancilla flag per noise irrep" is realisable on physical hardware, or whether the irreps are so abstract that selective dispersive coupling $L_k^{(\mu)} \otimes \sigma_z^{(a_\mu)}$ is impractical to engineer. We argue below that, for a class of noise models common in cQED and photonic platforms, the multi-flag construction reduces to hardware primitives already demonstrated in erasure-converted-qubit experiments, with overhead $O(N)$ in ancilla count. We then briefly indicate two further decompositions that warrant separate analysis.

The easy case: on-site Pauli noise on a planar cQED array. Consider an N-qubit transmon array with on-site noise dominated by single-qubit dephasing $L_i^{(Z)} = \sqrt{\gamma_Z}\sigma_z^{(i)}$ and amplitude damping $L_i^{(-)} = \sqrt{\gamma_1}\,\sigma_-^{(i)}$, which is the standard regime characterised by T_1/T_2 on every modern superconducting platform. The noise commutant decomposes site-by-site into two $\mathbb{Z}_2$ irreps per site, giving $2N$ irrep-flags in total. The selective coupling $L_i^{(\mu)} \otimes \sigma_z^{(a_{i,\mu})}$ for each site-irrep pair (i, μ) requires only:

- A per-site ancilla qubit (typically a tunable transmon or fluxonium) detuned from the data qubit by an irrep-specific frequency.
- A fixed dispersive cross-Kerr coupling of the type implemented routinely in cQED [1, 2]
- A terminal projective measurement of each ancilla in the $\sigma_x^{(a_{i,\mu})}$ basis, which is a single dispersive readout pulse.

The same architecture has been demonstrated for erasure-converted dual-rail qubits [3, 4], where one ancilla flag per data qubit is used to herald loss events at no simulator cost. Our multi-flag construction reuses this hardware primitive *unmodified*; the only difference is that we engineer the dispersive coupling to herald *noise* rather than *loss*, with the irrep label playing the role of a generalised loss-channel index. Hardware overhead is $O(N)$ ancilla qubits, comparable to the syndrome-extraction overhead of any error-corrected architecture, and strictly smaller than the surface-code overhead at the same logical distance. Crosstalk between dispersive couplings is the same engineering constraint that erasure-converted qubits already meet.

For noise with a known few-body correlated structure – e.g. correlated two-body dephasing $L = \sqrt{\gamma_{ZZ}}\,\sigma_z^{(i)}\sigma_z^{(j)}$ in the analogue Hamiltonian simulation, the irrep count remains $O(1)$ per pair, and a single dispersive cross-Kerr coupling on a shared ancilla is sufficient. Few-body correlated noise on photonic platforms (e.g. correlated photon loss in nearest-neighbour cavities) is structurally identical and is implementable via the same dispersive-coupling primitive.

Limitation. The construction does require that the irrep decomposition itself be known. For noise with continuous spectrum ($1/f$ noise, charge noise) or unknown commutant, the multi-flag construction reduces to single-flag with a global a_S-rate bound; we recover the bare-protocol scaling and lose the per-irrep variance factorisation. For strongly non-Markovian bath memory, the per-irrep factorisation degrades. Within these limits, multi-flag is a structural refinement, not a replacement.

Further decompositions warranting separate analysis. Two physical reorganisations of the flag register that we have not analysed but that could reduce ancilla count or align better with bath structure are as follows:

(i) *Schur–Weyl-encoded flags* for collective or permutation-symmetric noise – e.g. collective dephasing $L = \sqrt{\gamma}\sum_i \sigma_z^{(i)}$ on an atomic-ensemble or Dicke-coupled cavity-QED platform, where the noise acts non-trivially only on the totally-symmetric multiplicity space – in which the multiplicity space of each irrep is exploited via a Bacon–Chuang–Harrow Schur transform [5] to compress an $O(N)$-irrep register into $O(\log N)$ ancillas at the cost of a non-trivial Schur circuit

(ii) *Spectrally-resolved flags*, in which each ancilla is dispersively coupled to a specific bath-mode bandpass (a filter cavity tuned to a particular frequency window) rather than to a specific Lindblad operator – natural for cQED platforms whose bath has well-separated spectral peaks (e.g. a transmon coupled to a structured bath of distinct purcell-filter modes, or an optomechanical system with isolated phonon branches) – giving a spectrally-decomposed heralding pattern that aligns with the hardware's natural mode structure rather than with the Lindbladian's algebraic one.

Both extensions are compatible with the projector-collapse dynamics and the per-flag selection rule, but the corresponding sample-complexity bounds (compression overhead in (i); spectral-overlap-weighted variance in (ii)) require separate derivation and are deferred to future work.

IV. SUPPLEMENTARY NOTE 4: EXPLICIT CORRESPONDENCE WITH AN ERASURE CHANNEL

A. Setting: dephasing the classical flag

The bare-ancilla protocol couples each system jump operator L_k to an ancilla via *two* Lindblad terms, $\mathcal{D}[L_k \otimes \mathbb{I}]$ and $\mathcal{D}[L_k \otimes \sigma_z^{(a)}]$, together with the $\sqrt{S}$ regulariser ($S = a_S\mathbb{I} - \sum_k L_k^\dagger L_k$),

$$\tilde{\mathcal{L}}[\rho_{\rm tot}] = -i[H_S \otimes \mathbb{I}, \rho_{\rm tot}] + \sum_k \big(\mathcal{D}[L_k \otimes \mathbb{I}] + \mathcal{D}[L_k \otimes \sigma_z^{(a)}]\big)(\rho_{\rm tot}) + \big(\mathcal{D}[\sqrt{S} \otimes \mathbb{I}] + \mathcal{D}[\sqrt{S} \otimes \sigma_z^{(a)}]\big)(\rho_{\rm tot}). \tag{15}$$

Expanding each dissipator and using $\sigma_z^2 = \mathbb{I}$, the $\{L_k^\dagger L_k,\ \rho\}$ anticommutator pieces of the $L_k \otimes \mathbb{I}$ and $L_k \otimes \sigma_z^{(a)}$ dissipators are equal and add (rather than cancelling), and likewise for the $\sqrt{S}$ pair; the total anticommutator weight reduces to $-2a_S\rho_{\rm tot}$ via the regulariser identity $\sum_k L_k^\dagger L_k + S = a_S\mathbb{I}$. The sandwich pieces, in contrast, combine into $(1 + \sigma_z{\cdot}\sigma_z)/2 \cdot 2 = \mathcal{P}_{1/2}[\mathbb{I} \otimes \sigma_z^{(a)}] \cdot 2$. The master equation collapses to

$$\dot{\rho}_{\rm tot} = -i[H_S \otimes \mathbb{I}, \rho_{\rm tot}] - 2a_S\rho_{\rm tot} + 2\,\mathcal{P}_{1/2}\Big[\mathbb{I} \otimes \sigma_z^{(a)}\Big]\Big(\sum_k (L_k \otimes \mathbb{I})\rho_{\rm tot}(L_k^\dagger \otimes \mathbb{I}) + (\sqrt{S} \otimes \mathbb{I})\rho_{\rm tot}(\sqrt{S} \otimes \mathbb{I})\Big), \tag{16}$$

where $\mathcal{P}_p[O](\rho) = (1-p)\rho + pO\rho O^\dagger$ is the stochastic-application channel and $\mathcal{P}_{1/2}[\mathbb{I} \otimes \sigma_z^{(a)}]$ is the projector onto the $\sigma_z^{(a)}$-eigenbasis of the ancilla (it satisfies $\mathcal{P}_{1/2}^2 = \mathcal{P}_{1/2}$ since $\sigma_z^2 = \mathbb{I}$ and $\sigma_z = \sigma_z^\dagger$).

The only operator acting on the ancilla in the engineered Lindbladian is $\mathbb{I} \otimes \sigma_z^{(a)}$, which appears only inside the projector $\mathcal{P}_{1/2}$. Each system noise event *collapses the ancilla in the* $\sigma_z^{(a)}$*-basis*; it does not rotate or flip $|+\rangle$ to $|-\rangle$. The system and ancilla therefore remain in a separable joint state for all time, with only classical correlations: the ancilla is a *classical flag* that has either been collapsed (a noise event occurred at some point) or not.

B. Erasure: the $\sigma_x^{(a)}$ projector kills collapsed branches

Initialise the ancilla in $|+\rangle$, so $\langle\sigma_x^{(a)}\rangle_{|+\rangle\langle+|} = 1$. Two simple facts then determine everything:

(i) The ideal Hamiltonian piece $H_S \otimes \mathbb{I}$ acts as the identity on the ancilla, so it preserves $\langle\sigma_x^{(a)}\rangle$.

(ii) The collapse projector $\mathcal{P}_{1/2}[\mathbb{I} \otimes \sigma_z^{(a)}]$ diagonalises the ancilla in the $\sigma_z^{(a)}$-basis, so once it has been applied (even once) the ancilla expectation of $\sigma_x^{(a)}$ (or $\sigma_y^{(a)}$) is zero.

Combining (i)–(ii) with the Poisson statistics of noise events: the probability that *no* collapse has occurred by time t is e^{-2a_St}, matching the explicit decay factor in (16). On this "no-collapse" branch the ancilla still reads $|+\rangle$ (so contributes 1 to $\langle\sigma_x^{(a)}\rangle$) and the system has evolved purely under H_S, i.e. noise-free. On every other branch the ancilla has been collapsed and contributes 0. Hence

$$\big\langle A_S \otimes \sigma_x^{(a)}\big\rangle_{\rho_{\rm tot}(t)} = e^{-2a_St}\,\big\langle A_S\big\rangle_{\rho_S^{\rm ideal}(t)}, \qquad \rho_S^{\rm ideal}(t) := e^{-iH_St}\rho_S(0)e^{iH_St}, \tag{17}$$

and the protocol's recovered estimator $e^{2a_St}\langle A_S \otimes \sigma_x^{(a)}\rangle$ inverts the prefactor to return $\langle A_S\rangle_{\rho_S^{\rm ideal}(t)}$.

This is the *erasure-channel form,* but with a different operational flavour from a standard heralding code:

- No post-selection is performed. Every shot contributes to the estimator. The $\sigma_x^{(a)}$ projector *automatically discards* the contributions of collapsed branches via the rep-theoretic orthogonality $\langle\sigma_z\sigma_x\rangle = 0$, not via active classical feedforward.
- The classical flag is the ancilla's coherence in the $\sigma_x^{(a)}$ direction: pristine ($\langle\sigma_x^{(a)}\rangle = 1$) iff no collapse has occurred, decohered ($\langle\sigma_x^{(a)}\rangle = 0$) otherwise.
- The rescaling factor e^{2a_St} is the inverse no-collapse probability, exactly the heralding overhead of an ideal erasure code.

C. Equivalence with explicit $\sigma_x^{(a)}$-measurement

The same recovered expectation can be obtained with an explicit projective measurement of the ancilla in the $\sigma_x^{(a)}$-basis at the final time, post-selecting on the $|+\rangle$-outcome. By (17) the $|+\rangle$-outcome occurs with probability

$$\Pr\Big[\sigma_x^{(a)}=+1\Big]=\tfrac{1}{2}(1+e^{-2a_S t}),\qquad \Pr\Big[\sigma_x^{(a)}=-1\Big]=\tfrac{1}{2}(1-e^{-2a_S t}),\tag{18}$$

and the conditional system state on the $|+\rangle$-branch contains a weighted mixture of the noise-free state (with weight $e^{-2a_S t}$) and the fully-collapsed state (with weight $1-e^{-2a_S t}$); the $|-\rangle$-branch contains only the fully-collapsed state with the opposite sign. The standard erasure-style heralded estimator $[\Pr(+)\langle A_S\rangle_+ - \Pr(-)\langle A_S\rangle_-]/[\Pr(+)-\Pr(-)] = e^{2a_S t}\langle A_S\otimes\sigma_x^{(a)}\rangle$ recovers the noise-free expectation.

D. Generalisation to block-resolved heralding

The argument generalises directly to the multi-flag construction of Sec. III. Each irrep block of L couples to its own flag with the same $L^{(\mu)}\otimes\sigma_z^{(\mu)}$-vs-$L^{(\mu)}\otimes\mathbb{I}$ pair, producing a per-block projector $\mathcal{P}_{1/2}[\mathbb{I}\otimes\sigma_z^{(\mu)}]$; a jump in irrep μ collapses only the μ-flag. Reading $\sigma_x^{(\mu_A)}$ on the matched flag, while requiring all other flags read $+1$ (no spurious cross-block collapses), then gives the block-resolved erasure

$$\Big\langle A_{\mu_A}\otimes\sigma_x^{(\mu_A)}\prod_{\mu\neq\mu_A}P_+^{(\mu)}\Big\rangle = e^{-2\lambda_{\mu_A}t}\,\big\langle A_{\mu_A}\big\rangle_{\rho_S^{\mathrm{ideal}}(t)},\tag{19}$$

where $P_+^{(\mu)}=(\mathbb{I}+\sigma_x^{(\mu)})/2$ is the post-selection projector and λ_{μ_A} is the per-block noise rate. The exponent $2\lambda_{\mu_A}$ (rather than $2a_S$) is the inverse no-collapse probability *for the μ_A-block alone*, which is consistent with the rescaling factor in Sec. III.

V. SUPPLEMENTARY NOTE 5: COHERENT ANCILLA MISCALIBRATION AND ENCODED-ANCILLA CHECKS

A. Bare-ancilla first-order robustness

In the main manuscript (Methods), we have shown that the proposed protocol is robust against the coherent ancilla miscalibration with vanishing first-order contribution. This can be alternatively formalized based on the symmetry between the generator of the ancilla state and the final measurement. The ancilla's initial state $|+\rangle_a$ can be characterized by the stabiliser $g = \sigma_x^{(a)}$ with $g|+\rangle = X = (+1)|+\rangle$ and the ancilla part of the final measurement is also given as $\sigma_x^{(a)} = g$. Now suppose that the final measurement is also perturbed as $\tilde{\sigma}(\delta) = e^{i\delta Q} g e^{-i\delta Q} = g + i\delta[Q, g] + \mathcal{O}(\delta^2)$ with a Hermitian operator Q and a small misalignement parameter δ. As we discussed in the main text, the final state after the mitigation dynamics can be expressed as

$$W(T) = \frac{1}{2}\left[\tilde{\rho}(T) \otimes \mathbb{I} + e^{-2aT}\rho_{\text{ideal}}(T) \otimes \sigma_x\right] = \frac{1}{2}\left[\tilde{\rho}(T) \otimes \mathbb{I} + e^{-2aT}\rho_{\text{ideal}}(T) \otimes g\right]. \tag{20}$$

By taking the expectation value of $A \otimes \tilde{\sigma}(\delta)$, we obtain

$$\begin{aligned}\langle A \otimes \tilde{\sigma}(\delta)\rangle &= \frac{1}{2}e^{-2aT}\rho_{\text{ideal}}(T)(\text{Tr}[\mathbb{I}] + i\delta\text{Tr}(g[Q, g]) + \mathcal{O}(\delta^2)) \\ &= e^{-2aT}\rho_{\text{ideal}}(T)(1 + \mathcal{O}(\delta^2)),\end{aligned} \tag{21}$$

from the fact that $\text{Tr}[\tilde{\sigma}(\delta)] = 1$ and $\text{Tr}(g[Q, g]) = 0$ for any Q. This provides the first-order robustness of the coherent ancilla miscalibration given by any rotation. We note that Ref. [6]'s signed-recovery PEC protocol also falls in this class.

B. Distance-2 ancilla error detection protocol

To achieve a further reduction of the error in higher orders, one requires a condition on the nested commutator; e.g., vanishing δ^2 implies $\text{Tr}(g[Q, [Q, g]]) = 0$. Assuming both Q and g are Pauli operators, this condition holds only in the commuting case, i.e., $[Q, g] = 0$. Therefore, one cannot entirely remove δ^2 terms when considering a single-qubit ancilla with a single generator g. This resembles the situation in quantum error correction, where the noise is not correctable if it induces a logical error.

Consequently, this issue can be circumvented by considering an error correction or detection protocol of ancilla states, supported by additional stabilisers. As a minimal example, we consider a parity-checked encoding of the ancilla into a stabiliser code that detects weight-1 ancilla errors (e.g. $|0_L\rangle = |00\rangle$, $|1_L\rangle = |11\rangle$ with stabiliser Z_1Z_2, or the [[4,2,2]] code). In this example, the initial state prepared in $|+\rangle_L = \frac{|00\rangle+|11\rangle}{\sqrt{2}}$ is now stabilised by a set of Pauli operators $\{X_1X_2, Z_1Z_2, -Y_1Y_2\}$. Then, by post-selecting the ancilla states only for states $|+\rangle_L$ or $|-\rangle_L$ (i.e., with stabilizers $\{\pm X_1X_2, Z_1Z_2, \mp Y_1Y_2\}$, but discarding the other states spanned by $\{|01\rangle, |10\rangle\}$ some additional δ^2 term can be further suppressed for local coherent noise, while still guaranteeing a full suppression of the linear order in δ.

We have verified numerically that the bias continues to scale as δ^2 rather than δ^4 under both codes (see the numerical sweep below), yet provides a $\sim 11\times$ prefactor reduction. The reason that stabiliser-distance arguments do *not* lift to δ^4 in this continuous-time setting is structural: continuously-on coherent ancilla detuning perturbs the heralding mechanism itself throughout the evolution, producing an $O(\delta^2)$ bias that error *detection* cannot remove without disrupting the herald. Reaching δ^4 would require either error-correcting (rather than error-detecting) ancilla codes, or discrete-time herald refresh – both of which trade off against the protocol's continuous-time character; we leave these as open directions.

We also verified that the terminal acceptance check associated with the distance-2 code does not reduce the per-shot variance: the distance-2 estimator's per-accepted variance is essentially identical to the bare estimator's variance at small δ, with the only effective-sample-cost penalty being the $1/p_{\text{accept}} = 1 + O(\delta^2)$ acceptance-rate factor. The distance-2 gain is a bias-prefactor reduction at neutral variance cost, not a variance-axis exponent reduction. The variance-axis exponent reduction *is* achievable, separately, by the multi-flag block-resolved heralding of Sec. III – but that mechanism is orthogonal: it resolves the noise commutant rather than encoding the ancilla.

C. Numerical demonstration

We perform numerical simulation for a single-qubit dephasing noise $\mathcal{D}[\sqrt{\gamma}\sigma_z]$ with $\gamma_z = 0.3$ under the Hamiltonian $H = \frac{\sigma_y}{2}$. A coherent ancilla detuning generated by σ_y. For $\gamma_z T = 0.3$, we obtain the following results:

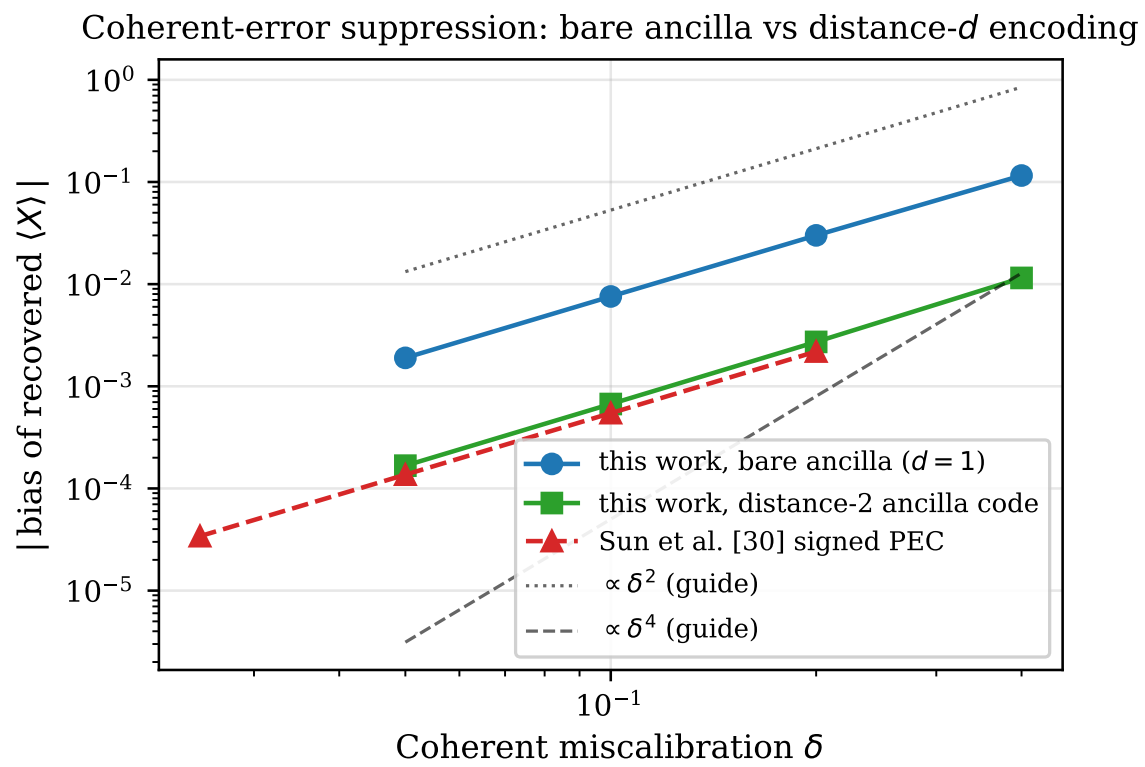

δ	bare-ancilla bias	distance-2 bias	suppression	δ^2
0.05	-1.90×10^{-3}	$+1.67 \times 10^{-4}$	$\sim 11\times$	2.5×10^{-3}
0.10	-7.57×10^{-3}	$+6.71 \times 10^{-4}$	$\sim 11\times$	1.0×10^{-2}
0.20	-3.00×10^{-2}	$+2.72 \times 10^{-3}$	$\sim 11\times$	4.0×10^{-2}
0.40	-1.16×10^{-1}	$+1.15 \times 10^{-2}$	$\sim 10\times$	1.6×10^{-1}

Supplementary Figure 1. Coherent-error suppression: bias of the recovered observable vs. miscalibration angle δ, at $\gamma T = 0.3$. Both bare-ancilla protocol and [[4,2,2]] error-detecting code scales as δ^2, while the latter has a $\sim 11\times$ smaller prefactor. Sun et al. [6]'s signed-recovery PEC also scales as δ^2 at this benchmark.

D. Leakage out of the ancilla subspace

Leakage out of the ancilla computational subspace is not captured by an independent qubit Lindblad operator. We model leakage as population transfer from the protected subspace $\mathcal{H}_{0,1}$ to an orthogonal leakage subspace. If leakage is detectable, an encoded ancilla register can convert it into a flagged event. Such an encoding may be used either to reject flagged shots, with an acceptance overhead, or to correct the leakage if the hardware supports reset or recovery of the ancilla register. This encoded-ancilla variant is an optional robustness extension. The bare protocol itself remains a final-measurement protocol and does not rely on postselection.

As we discussed above, in the simplest parity encoding, $|0_L\rangle = |00\rangle$ and $|1_L\rangle = |11\rangle$, a single-qubit leakage or bit-flip event changes the stabiliser $Z_1 Z_2$ and can be detected at the terminal check. More general stabiliser or bosonic encodings can be used when the physical ancilla is an oscillator or cavity mode. These encodings protect the ancilla register without modifying the simulator Hamiltonian, which is why they are naturally compatible with the present system–ancilla architecture.

E. Connection to erasure-converted qubits

Within the erasure-channel framing of Sec. IV, a coherent ancilla error plays the role of an *unflagged residual error* on the heralding register – a well-studied failure mode in erasure-converted superconducting and neutral-atom qubits, whose suppression via stabiliser-encoded ancillas is established in Refs. [3, 4, 7]. Our distance-2 error-detecting ancilla extension is the continuous-time analogue of these constructions, and the $\sim 11\times$ prefactor reduction is the corresponding biased-erasure threshold improvement at distance-2.

VI. SUPPLEMENTARY NOTE 6: FURTHER CONSIDERATIONS ON OTHER REALISTIC NOISE MODELS

A. Imperfect noise characterization and engineered-rate errors

The protocol is noise-model assisted: the jump operators and rates used in the mitigation Lindbladian must match the dominant dissipative channels to be mitigated. If the implemented recovery rate differs from the physical rate, for example $\gamma \to (1+\varepsilon_{\mathrm{rate}})\gamma$, the cancellation is no longer exact. To leading order, the mismatch leaves a residual effective generator of size $O(\varepsilon_{\mathrm{rate}}\gamma)$, and the corresponding observable bias accumulates over the evolution time.

B. Ancilla dephasing and local ancilla noise

Local Markovian ancilla noise changes the recovered off-diagonal block. For many single-ancilla noise models of the form

$$\mathcal{L}_{\mathrm{anc}}(W) = \mathcal{D}[\mathbb{I} \otimes M](W), \tag{22}$$

the dominant effect is an additional exponential damping of the recovered coherence, which can be absorbed into the final rescaling factor when the rate is known. This includes standard dephasing, relaxation, excitation, and Pauli noise channels on the ancilla. If the ancilla-noise rate is imperfectly known, the mismatch contributes to the residual bias.

C. System–ancilla crosstalk and correlated noise

Imperfect implementation of the engineered jump operators can induce correlated system–ancilla noise. This is the main mechanism by which hardware crosstalk enters the idealized model. A representative class is $\mathcal{D}[P \otimes \sigma_\pm]$, where P is a system Pauli string. Projecting onto the off-diagonal ancilla block gives

$$\langle 0| \mathcal{D}[P \otimes \sigma_\pm](W) |1\rangle = -\frac{1}{2} W_{01}, \tag{23}$$

so this class of correlated error produces a scalar damping of the recovered coherence and can be absorbed into the final rescaling factor when its rate is known.

A different representative class is $\mathcal{D}[P \otimes Z_a]$. For Pauli P one obtains

$$\langle 0| \mathcal{D}[P \otimes Z_a](W) |1\rangle = -PW_{01}P - W_{01}, \tag{24}$$

which is not generally proportional to W_{01} and therefore produces a structural residual bias. There is one important exception to this interpretation. If P is the same system operator as one of the intended engineered recovery jumps, then an additional channel $\mathcal{D}[P \otimes Z_a]$ has the same operator structure as the mitigation term itself and should be treated as a miscalibration of that engineered rate, rather than as a new independent noise mechanism. After recalibration, it is covered by the rate-error analysis of Sec. VI A. For a generic correlated channel not included in the intended recovery model, one may sometimes restore scalar damping by pairing it with a system-only term $\mathcal{D}[P \otimes \mathbb{I}]$, whose contribution is $PW_{01}P - W_{01}$; the combined effect is then proportional to W_{01}.

D. Combined realistic-noise simulation

To test a setting closer to a real experiment, we provide additional simulation results for the 2D anisotropic Heisenberg model

$$H = \sum_{\langle ij \rangle} \left[J_x \sigma_x^{(i)} \sigma_x^{(j)} + J_y \sigma_y^{(i)} \sigma_y^{(j)} + J_z \sigma_z^{(i)} \sigma_z^{(j)} \right] - h \sum_{i=1}^{4} \sigma_y^{(i)}, \tag{25}$$

studied in the main text. We also take the same Hamiltonian parameters ($J_x = J(1+\gamma)$, $J_y = J(1-\gamma)$, and $J_z = J$ with the anisotropy parameter $\xi = 0.2$ and the strength of the pairwise interaction $J = 2$ and transverse field $h = 0.1$) and the noise model (dephasing $\sqrt{\gamma_z}\sigma_z$ and relaxation $\sqrt{\gamma_-}\sigma_-$ with $\gamma_z = \gamma_- = 0.03$ for **both system and ancilla qubits**) as in the main text. The following realistic noise induced by miscalibrations and the recovery dynamics are additionally considered:

(i) Noise rate miscalibration + engineered-Lindbladian rate error: $\varepsilon_{\mathrm{rate}} = 2\%$

(ii) Coherent ancilla miscalibration: The final measurement becomes $A \otimes \tilde{\sigma}(\delta)$, where $\tilde{\sigma}(\delta) = e^{-i\delta/2\sigma_y}\sigma_x e^{i\delta/2\sigma_y} = (\cos\delta)\sigma_x - (\sin\delta)\sigma_z$ with $\delta = 0.05$ with the ancilla σ_y rotation.

(iii) Residual system-side crosstalk: $\sqrt{\gamma_{\mathrm{corr}}}(\sigma_z^{(i)} \otimes \tilde{\sigma}(\delta))$ for each qubit with correlated error rate $\gamma_{\mathrm{corr}} = 0.05\gamma_z$.

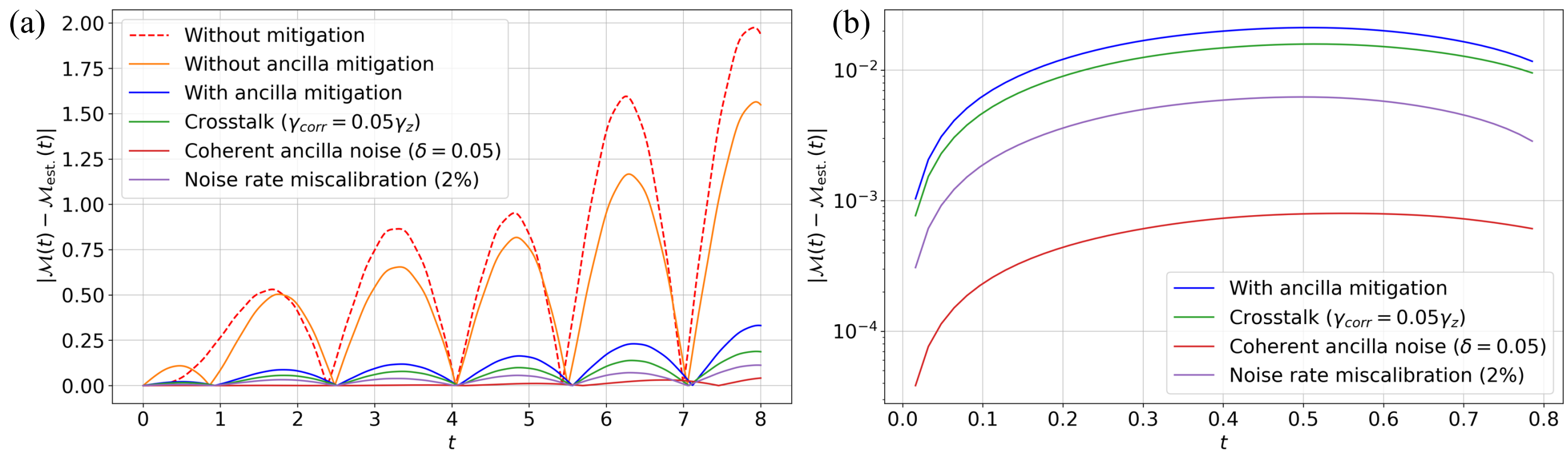

Supplementary Figure 2. Numerical simulation of the two-dimensional anisotropic Heisenberg model. (a) Bias relative to the ideal expectation value and (b) a breakdown of the bias for $t \leq 0.8$.

Figure 2 (a) shows the bias of the total magnetization $\mathcal{M} = \sum_{i=1}^{4} \sigma_z^{(i)}$ between the ideal and the mitigation dynamics. We note that the ancilla noise mitigation is the most crucial part for reducing the bias as it has the same rate as the system qubit's noise. Figure 2 (b) shows the further breakdown of the bias after applying the ancilla mitigation by independently simulating the additional noise/miscalibration. Among the remaining error sources, the correlated error induced by the crosstalk could contributes the most, while coherent ancilla error marginally affects the bias as it only scales with δ^2 as discussed in Sec. V

VII. SUPPLEMENTARY NOTE 7: COMPARISON WITH EXISTING CONTINUOUS-TIME QEM PROTOCOLS

A. Pulse-bandwidth constraint in impulsive recovery protocols

In the stochastic recovery protocol of Ref. [6] (Ref. [30] in the main manuscript), a recovery operation

$$\mathcal{B}_j = \exp(-iH_{\mathrm{kick}}\tau_{\mathrm{kick}}) \tag{26}$$

is applied at stochastic jump times drawn from a Poisson process. The ideal continuum limit corresponds to applying a fixed recovery rotation in the sharp-pulse limit $\tau_{\mathrm{kick}} \to 0$, which requires $\|H_{\mathrm{kick}}\| \to \infty$. With a bounded physical drive $\|H_{\mathrm{drive}}\| \leq H_{\max}$, a rotation by an angle θ_j requires

$$\tau_{\mathrm{kick}} \gtrsim \theta_j / H_{\max}. \tag{27}$$

During this finite pulse window, the background simulator Hamiltonian continues to act and produces a finite-bandwidth residual. This is consistent with Ref. [6], which identifies a per-pulse residual and requires the kick intensity to be sufficiently large compared with the simulator Hamiltonian. For a Poisson record with mean number of pulses $N_{\mathrm{pulse}} = \Gamma T$, these residuals accumulate. Thus maintaining an unbiased sharp-kick limit at fixed target accuracy requires increasing drive amplitude or bandwidth.

The present protocol avoids this particular pulse-bandwidth tradeoff because it does not use instantaneous trajectory-dependent recovery operations. The mitigation is generated by a bounded-norm, time-independent engineered Lindbladian. Moreover, for a physical jump L_k, the corresponding mitigation jump is $L_k \otimes Z_a$ and

$$\|L_k \otimes Z_a\|_\infty = \|L_k\|_\infty. \tag{28}$$

The engineered dissipative strength is therefore set by the native noise channel to be mitigated rather than by a parametrically stronger instantaneous drive. The pre-engineered-Hamiltonian variant discussed in Ref. [6] is complementary to our construction, but it remains associated with recovery operations at pre-sampled jump times and the corresponding signed-record estimator.

B. Influence-martingale protocols and jump-record cost

In the trajectory-based construction of Ref. [8] (Ref. [31] in the main manuscript), the mitigated estimator contains a classical influence weight

$$\mu[\omega] = \prod_{\ell \in \omega} \frac{r_{j_\ell}}{\Gamma_{j_\ell}} \exp\left[-\int_0^t (\Gamma' - \Gamma)\, ds\right], \tag{29}$$

where ω denotes the quantum-jump record. Evaluating Eq. (29) requires knowing which jumps occurred and when they occurred, followed by shot-by-shot classical post-processing. The ancilla construction cited in Ref. [8] provides a way to generate the required jump trajectories, but the ancilla is still monitored and the resulting click record remains an input to the estimator.

Our construction is different: it does not attempt to autonomously implement a generic influence martingale. Instead, we engineer a signed-erasure structure on an enlarged system–ancilla Hilbert space. The recovery jump

$$L_k \longrightarrow L_k \otimes \sigma_z^{(a)} \tag{30}$$

maps each relevant system jump to a parity flip of the ancilla. As a result, the trajectory-dependent sign reduces to a terminal ancilla parity,

$$\mu[\omega] \longrightarrow e^{2aT}(-1)^{n(\omega)}, \tag{31}$$

where $n(\omega)$ is the parity of the relevant jumps. The parity is stored coherently and is read out by a single final measurement of $\sigma_x^{(a)}$, while the magnitude e^{2aT} is a global rescaling factor. The usual QEM sampling cost is not removed; it appears as the global variance amplification e^{4aT} rather than as a trajectory-dependent martingale tail.

This distinction is also why the click record in Ref. [8] is not merely a bookkeeping convention that can generally be absorbed into autonomous quantum dynamics. The generic influence weight is a signed, trajectory-dependent c-number functional of the record. By contrast, the terminal observable $e^{2aT}\sigma_x^{(a)}$ exists in our protocol because we have engineered a special parity-plus-constant structure on the joint Hilbert space. Generic Lindblad dynamics need not possess such a parity decomposition.

C. Ensemble-averaged stochastic implementation versus trajectory-conditioned stochasticity

The stochastic-Hamiltonian implementation discussed in the main text introduces randomness only as an implementation tool for generating the target Lindbladian on average. No information about the stochastic Hamiltonian trajectory is used in the estimator; only the final expectation value of the joint observable is measured. This is distinct from trajectory-conditioned stochasticity in quasiprobability or influence-martingale protocols, where the sampled recovery labels or click record determine the weight assigned to each shot.

Thus, if stochastic Hamiltonians are used to realize the engineered dissipator, the stochasticity is ensemble averaged: reconstruction errors arise from finite-statistics estimation of $\langle A \otimes \sigma_x \rangle$, not from finite-statistics estimation of trajectory-dependent weights. A reservoir-based engineered-dissipation implementation would remove even this implementation randomness, at the cost of introducing an additional dissipative reservoir.

D. Numerical comparison with Monte-Carlo unravelling

We provide a comparison between our work and approaches in Refs. [6, 8] by simulating Monte-Carlo unravelling of a single-qubit dephasing noise (or equivalently, IID dephasing) with noise rate $\gamma = 0.3$ and evolution time $T = 2$ and by taking $K = 2 \times 10^5$ shots. The results are summarized as follows:

Scheme	emp. mean	emp. var.	$\max\lvert E\rvert$	top-1% var. share
Sun et al. [6]	-0.424	10.84	3.32	0.010
Donvil–Muratore-Ginanneschi [8]	-0.423	23.78	**180.3**	**0.395**
This work	-0.422	10.85	3.32	0.010

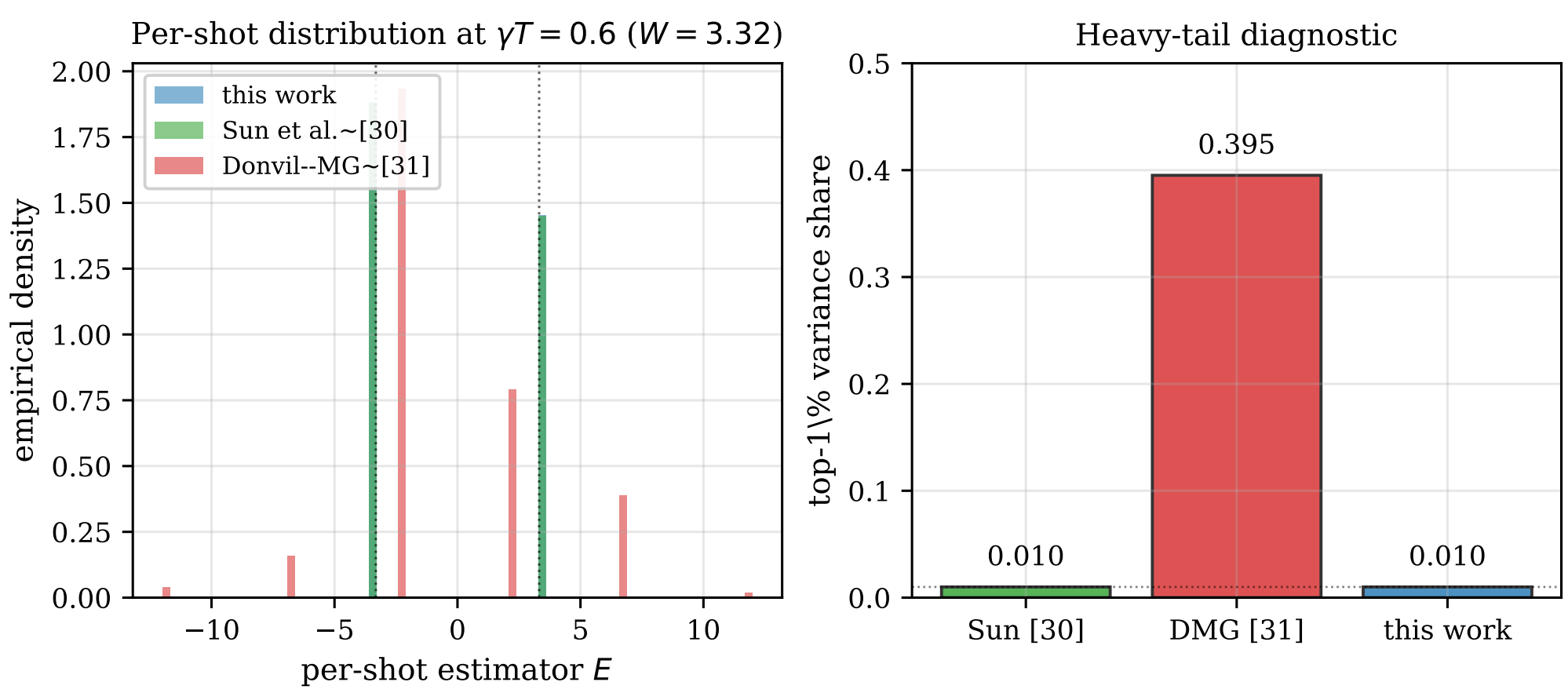

Supplementary Figure 3. Monte-Carlo unravelling of the three continuous-time QEM protocols at $\gamma T = 0.3$. Left: per-shot estimator densities. The bare estimator and Ref. [6]'s signed PEC have identical bounded support $\{-W, +W\}$; Ref. [8]'s influence martingale is heavy-tailed (note the long tails outside $\pm W$). Right: empirical top-1% variance share. Hoeffding applies to Ref. [6] and our scheme; only Bernstein applies to Ref. [8], with worse constants.

The numerical results in Fig. 3 support that Ref. [6] and our scheme are statistically equivalent in terms of bounded per-shot estimator support $[-W, +W]$, where $W = e^{2\gamma T} = 3.32$, with identical empirical variance and top-1% concentration. Hoeffding applies to both with the same constants. The advantages of our scheme over Ref. [6] in this regime are operational (no per-shot classical record of recovery operations, single-qubit-control localised to the ancilla, no signed-weight bookkeeping).

On the other hand, we note that Ref. [8]'s influence martingale is genuinely heavy-tailed. In the noise-cancellation regime ($\Gamma = \gamma/3$, $r = -3$ in the Donvil–Muratore-Ginanneschi parameterisation), $\max|E|$ exceeds W by a factor of ~ 54, and 40% of the empirical variance concentrates in the top 1% of shots. Hoeffding does not apply; Bernstein gives a worse constant. The resulting sample-complexity penalty is $\sim 10\times$ at $\varepsilon = 0.05$ and grows for tighter precision. This heavy-tailed behaviour has a structural origin: the influence-martingale weight $w_t = \exp\Big(\int_0^t r\, dN_s - \Gamma s\, ds\Big)$ has a moment-generating function that ceases to be sub-Gaussian once $|r| > 1$, which is precisely the noise-cancellation regime. Our scheme avoids this by lifting the modification into a CPTP generator on the joint system+ancilla register, where per-shot weights are bounded by construction in $[-W, +W]$.

E. Structural feasibility comparison under implementation constraints

We also discuss implementation concerns of the present protocol and compare with existing ones in Refs. [6, 8]. Several important comparison points are

- The herald is engineered ancilla-side, so bath memory appears as a model-mismatch correction to the engineered Lindbladian rather than as an additional per-shot trajectory record. This contrasts with schemes whose signed weight or martingale is built from a recovery or click record.
- The structural advantage of our scheme is that an ancilla error-detecting code can be deployed without modifying the simulator's Hilbert space. For example, a distance-2 encoding of ancilla states reduces the bias by an additional $\sim 11\times$ constant prefactor without additional operations on the system register as discussed in Sec. V. Ref. [6]'s recovery operations live on the *system register*, so any deeper protection requires changing the simulator's logical Hilbert space.
- In Refs. [6, 8], the recovery operations or designed reservoir must couple to every noisy system qubit individually. In our protocol, the engineered dissipator $L_k \otimes \sigma_z$ couples each system noise channel to a smaller number of ancilla register, and only the ancilla need be individually addressable. For analogue platforms where individual system-qubit addressing is the experimental bottleneck, e.g., cold-atom Hubbard simulators, large-array Rydberg simulators, this offers a substantial structural simplification.
- For local observables, both approaches of Ref. [6] and ours admit a *lightcone-restricted* variant that brings the prefactor down from $a_S = N\lambda_1$ to the in-cone Lindblad rate $\lambda_1|C(A_S, T)|$. Our multi-flag construction (Sec. III) further reduces the exponent to the per-sector spectral-norm rate $\lambda_{\mu_A} = \|\sum_k L_k^{(\mu_A)\dagger} L_k^{(\mu_A)}\|_\infty$ via sector selection on the ancilla. The mechanism is fundamentally ancilla-driven: the $\sigma_x^{(\mu_A)}$ readout resolves the μ_A-block content tagged by the matched flag at time T, paying only the per-sector physical Lindblad rate λ_{μ_A}; a parallel sectorwise Ref. [6] decomposition pays the per-sector quasiprobability normalization for its chosen inverse representation, and any unrestricted Ref. [6] recovery pays for the full lightcone. In the IID local-dephasing benchmark, the sector-heralded exponent replaces the lightcone volume by the measured Pauli weight, giving a factor $|C(A_S, T)|/w \sim (vT)^D/w$.

With the discussions above, the structural comparison sharpens as follows:

	**Sun et al. [6]**	**Donvil–Muratore-Ginanneschi [8]**	**This work**
Generator class	quasi-Lindblad (signed) on system	quasi-Lindblad (martingale) on system	**CPTP on joint S+A**
Estimator tail class	sub-Gaussian, bounded $\{-W, +W\}$	heavy-tailed (Bernstein-only)	sub-Gaussian, bounded $[-W, +W]$
Empirical top-1% var. share	0.010	$\mathbf{0.395}$	0.010
Concentration regime	Hoeffding	Bernstein only	Hoeffding
Per-shot classical record	$O(\lambda T)$ recovery labels	full quantum-jump trajectory	none
Classical post-processing per shot	$O(\lambda T)$ multiplications (sign accumulation)	full trajectory record + martingale product	one global rescaling
Wall-clock overhead vs unmitigated	runtime × sample count $e^{4\lambda T}$	runtime × sample count $e^{4\lambda T}$ + per-shot trajectory I/O	runtime × sample count $e^{4\lambda T}$
Single-qubit-control sites	every noisy system qubit	every noisy system qubit	ancilla register only
Mid-circuit measurement	not required	required (or designed reservoir)	not required
Sampling-overhead, global obs.	$e^{4a_S T}$	$e^{4a_S T}$	$e^{4a_S T}$
Sampling-overhead, weight-w local obs.	$e^{4\lambda_1 \lvert C(A_S,T)\rvert T}$ *(via lightcone-restricted recovery)*	$e^{4\lambda_1 \lvert C(A_S,T)\rvert T}$ *(via lightcone-restricted unravelling)*	$\boldsymbol{e^{4w\lambda_1 T}}$ *(multi-flag, Sec. III)*
Coherent-error bias	implicit $O(\delta^2)$ on system	implicit $O(\delta^2)$ on system	$O(\delta^2)$ on ancilla, $\sim 11\times$ prefactor reduction with distance-2 detection
Protection register	system register	system register	ancilla register

[1] J. Koch, T. M. Yu, J. Gambetta, A. A. Houck, D. I. Schuster, J. Majer, A. Blais, M. H. Devoret, S. M. Girvin, and R. J. Schoelkopf, Charge-insensitive qubit design derived from the cooper pair box, Phys. Rev. A **76**, 042319 (2007).

[2] M. Reagor, W. Pfaff, C. Axline, R. W. Heeres, N. Ofek, K. Sliwa, E. Holland, C. Wang, J. Blumoff, K. Chou, M. J. Hatridge, L. Frunzio, M. H. Devoret, L. Jiang, and R. J. Schoelkopf, Quantum memory with millisecond coherence in circuit qed, Phys. Rev. B **94**, 014506 (2016).

[3] A. Kubica, A. Haim, Y. Vaknin, H. Levine, F. Brandão, and A. Retzker, Erasure qubits: Overcoming the T_1 limit in superconducting circuits, Phys. Rev. X **13**, 041022 (2023).

[4] K. Sahay, J. Jin, J. Claes, J. D. Thompson, and S. Puri, High-threshold codes for neutral-atom qubits with biased erasure errors, Phys. Rev. X **13**, 041013 (2023).

[5] D. Bacon, I. L. Chuang, and A. W. Harrow, Efficient quantum circuits for schur and clebsch-gordan transforms, Phys. Rev. Lett. **97**, 170502 (2006).

[6] J. Sun, X. Yuan, T. Tsunoda, V. Vedral, S. C. Benjamin, and S. Endo, Mitigating realistic noise in practical noisy intermediate-scale quantum devices, Phys. Rev. Appl. **15**, 034026 (2021).

[7] H. Levine, A. Haim, J. S. C. Hung, N. Alidoust, M. Kalaee, L. DeLorenzo, E. A. Wollack, P. Arrangoiz-Arriola, A. Khalajhedayati, R. Sanil, H. Moradinejad, Y. Vaknin, A. Kubica, D. Hover, S. Aghaeimeibodi, J. A. Alcid, C. Baek, J. Barnett, K. Bawdekar, P. Bienias, H. A. Carson, C. Chen, L. Chen, H. Chinkezian, E. M. Chisholm, A. Clifford, R. Cosmic, N. Crisosto, A. M. Dalzell, E. Davis, J. M. D'Ewart, S. Diez, N. D'Souza, P. T. Dumitrescu, E. Elkhouly, M. T. Fang, Y. Fang, S. Flammia, M. J. Fling, G. Garcia, M. K. Gharzai, A. V. Gorshkov, M. J. Gray, S. Grimberg, A. L. Grimsmo, C. T. Hann, Y. He, S. Heidel, S. Howell, M. Hunt, J. Iverson, I. Jarrige, L. Jiang, W. M. Jones, R. Karabalin, P. J. Karalekas, A. J. Keller, D. Lasi, M. Lee, V. Ly, G. MacCabe, N. Mahuli, G. Marcaud, M. H. Matheny, S. McArdle, G. McCabe, G. Merton, C. Miles, A. Milsted, A. Mishra, L. Moncelsi, M. Naghiloo, K. Noh, E. Oblepias, G. Ortuno, J. C. Owens, J. Pagdilao, A. Panduro, J.-P. Paquette, R. N. Patel, G. Peairs, D. J. Perello, E. C. Peterson, S. Ponte, H. Putterman, G. Refael, P. Reinhold, R. Resnick, O. A. Reyna, R. Rodriguez, J. Rose, A. H. Rubin, M. Runyan, C. A. Ryan, A. Sahmoud, T. Scaffidi, B. Shah, S. Siavoshi, P. Sivarajah, T. Skogland, C.-J. Su, L. J. Swenson, J. Sylvia, S. M. Teo, A. Tomada, G. Torlai, M. Wistrom, K. Zhang, I. Zuk, A. A. Clerk, F. G. S. L. Brandão, A. Retzker, and O. Painter, Demonstrating a long-coherence dual-rail erasure qubit using tunable transmons, Phys. Rev. X **14**, 011051 (2024).

[8] B. I. C. Donvil, R. Lechler, J. Ankerhold, and P. Muratore-Ginanneschi, Quantum trajectory approach to error mitigation, Phys. Rev. A **111**, 042619 (2025).
