# Supplementary material for: Continuous-time noise mitigation in analogue quantum simulation
Source: Nat Commun. 2026 Oct 12;17:11557. doi: 10.1038/s41467-026-78310-5 (PMC13748635; doi:10.1038/s41467-026-78310-5)
Supplement: Supplementary file 2 [file 41467_2026_78310_MOESM2_ESM.pdf]

nature portfolio

Peer Review File

Continuous-time noise mitigation in analogue quantum simulation

Corresponding Author: Professor Myungshik Kim

This file contains all reviewer reports in order by version, followed by all author rebuttals in order by version.

A version of this paper was originally rejected for publication by Nature Communications, however that decision was reconsidered after appeal by the authors.

Version 1:

Reviewer comments:

Reviewer #1

(Remarks to the Author)
This manuscript introduces a method for quantum error mitigation in continuous time. The topic is very timely. The development of quantum processors has followed a path in recent years where the number of qubits is not necessarily the main bottleneck, but rather their quality or fidelity is. That means that approaches where auxiliary qubits are used to mitigate errors are feasible in many experimental platforms. While naturally this is how quantum error correction works, such techniques typically demand very large overheads as well operating in discrete time, often introducing errors of its own. The proposal by the authors uses auxiliary qubits to correct errors in continuous time, and is thus tailored for analog quantum simulation.

Concretely, the method requires introducing an auxiliary system (which could be as simple as a single qubit) and engineering the dissipation of the new, extended system. Upon evolution, the noiseless expectation value of some operator A can be related to the expectation value of A \otimes X in the extended system.

I think the method is clever and the fact that it is general and leads to exact noise cancellation makes it an noteworthy result. The main question mark is how feasible it would be to implement this in practice. In my view there are two main complications: engineering the dissipative dynamics, and getting around the sampling overhead. For the first aspect, in the most general case, engineering the dissipator of Eq. (3) could be quite challenging, if possible at all. For qubit systems, luckily, the problem of Lindbladian engineering is relatively studied. As the authors state, the method's complexity is greatly reduced if the dissipation is such that \sum L^dagger L \propto I, which would happen for e.g. unital maps. This leads to a situation where the dissipation can be engineered by implementing a noisy Hamiltonian, as described in the section "Physical implementation". This scenario is relevant thus making their proposal appealing to a broad enough audience in the field.

The issue of the sampling overhead is addressed by the authors, however I have some concerns about this. The main one is about scaling with N, as I detail below.

The authors have done a good job in addressing other 'sources' of concern, namely how the protocol performs in presence of noise in the ancilla (beyond the engineered dissipation) and in the event of non perfect knowledge of the noise channels. I've found their treatment of these topics satisfactory, although I have a few comments and questions which will be detailed below. I've found it to be refreshing that the authors test their method not only in the problem of obtaining expectation values of macroscopic observables (which tend to be inherently robust or relatively predictable in presence of dissipation) but also more complicated quantities like the Loschmidt echo and the spectral response.

In summary, the paper is well-written, most ideas are presented in a clear way, and the analysis is compelling, with the caveat of the scaling with N. The results are noteworthy and important, and have the potential to impact future experiments. The weakness I've found is that the paper lacks a more detailed analysis and comparison of the cost or complexity of this method as compared to other QEM approaches. For me to recommend this paper for publication, I would ask the authors to address my main comments below.

Main comments

1. Does a scale with N? From the example of the Heisenberg model, this seems to be the case. If so, the authors should address this point. Does this mean that the sampling overhead scales exponentially with N?

2. In the discussion regarding sampling overhead, it is stated that sampling overhead scaling exponentially with noise rate and time is “expected from all QEM protocols”. I think the authors should expand on this point - what is the intuition behind it? what are other QEM protocols which share this feature, and what are the references to previous studies?

3. Related to this point, the proposed protocol assumes knowledge of $D[\rho]$, the dissipator. I think the paper would be strengthened by emphasizing this issue earlier on (before the robustness analysis). How does this requirement relate to the the wider QEM method zoo (where such knowledge is also required, to more or less extent), and ideally also connecting it to noise learning methods from which this approach could benefit.

4. Is the calculation of ‘a’ potentially a bottleneck of the model? In the example described this is done explicitly for N = 4 spins, but the case is relatively trivial and seems to extend ok to other N (although it might lead to an unwanted scaling of a with N).

Minor comments

The authors seem to focus on quantum simulation as an application, but it seems their results could be applicable also to adiabatic quantum computing. Is this correct or do you envision specific hurdles in this?

In page 2, at the beginning of the ‘Noise mitigation protocol’ section it says that the Hamiltonian can be time-dependent, but in the previous paragraph the contrary seems to be stated. Which one is correct?

Reviewer #2

(Remarks to the Author)
Referee report

Continuous-time noise mitigation in analogue quantum simulation

This paper discuss the QEM protocol for analog simulator. The proposed method is as follows. They propose to prepare the ancilla plus state and design the Lindblad operator in the composite system of ancilla+system such that the $\mathrm{Tr}[A\otimes\sigma_x \, \rho_{com}]$ (ρ_{com} is the density matrix in the composite system) uniformly shrinks compared with the ideal result with the $e^{(2a t)}$(Eq. (7)) with a being a constant determined by the Lindbladian. Then, by rescaling the result by $e^{(2a t)}$, I think the idea is interesting.

Nevertheless, I think the novelty of this work does not reach the novelty and practicality expected in nature communications. First, the novelty should lie in the their protocol is truly the first continuous QEM protocol without discretization. However, this is based on the author’s misunderstanding of the previous work. To my knowledge, Refs. [30] and [31] do not assume discretization of the process. For example, Ref [30] first discretize the continuous process to use the idea of the original PEC first, but they clearly show that the limit of $\delta t \to 0$ can be taken by using the stochastic approach which determines the QEM jump operation stochastically (See Sec IV and V in Ref. [30]). Although it is true that this protocol requires interleaving single-qubit pulse operations and some commutation error with the original Hamiltonian may occur when switching off the original Hamiltonian is not possible, there is no error due to discretization. It is not ideal to base the novelty claim on an inaccurate characterization of the contributions of prior works. At least, the authors should clarify what is truly continuous error mitigation from the mathematical level. I guess the introduction and framework section need to be changed significantly to have the clear comparison with the prior works. The authors need to thoroughly compare their method with the prior methods numerically, by correctly understanding simulating the prior methods. The same authors seem to have a similar discussion in their prior work. https://journals.aps.org/pra/abstract/10.1103/PhysRevA.109.012431

In addition, while I think the proposed method seems novel, I doubt the practicality of their method. This method needs to design the complicated Lindblad operator, such as $\sigma_z^A\otimes\sigma_z^B$ for mitigating dephasing errors in the system. However, to design such Lindblad operators, we need another ancilla system and require three-body interaction. This is in principle possible, but the expected error to realize Lindblad operators is significant. The effect of crosstalk to the system is not negligible. The aim of error mitigation is to mitigate errors with easy operations, so this seems a bit contradiction to me. At least, the authors need to numerically simulate the realistic noise model and compare their result with methods in Refs. [30, 31] with respect to the sampling overhead and the feasibility and runtime of QEM routine.

Overall, I think the proposal is interesting, and I like the idea. I suppose their method may be more useful for algorithm design than error mitigation. I suppose the authors have a misunderstanding about the prior works, and doubt the practicality. I recommend the authors transfer the paper to more specialized journal after addressing my comments.

Reviewer #3

(Remarks to the Author)
This work proposes a continuous-time error mitigation protocol for analogue quantum simulators based on engineered dissipative coupling to ancillas. By embedding the system into a larger Hilbert space and carefully constructing joint Lindblad operators, the authors show that the system's noise can be cancelled in the off-diagonal blocks of the joint density matrix. This central idea is simple and elegant. As such, the ideal noiseless evolution may be then recovered via measurement and classical post-processing.

I like this idea which avoids discretization and physically implementing a "negative dissipator"like stochastic error mitigation. The illustrative examples demonstrate feasibility under simplified conditions. Though, I have a few concerns on the assumptions, schemes and the claimed advantages as outlined in the following.

About the advantages and distinction to PEC and analogue simulation.

At the level of the Lindbladian description, the scheme is deterministic and does not rely on quasi-probability sampling, which is the main conceptual innovation of this work. However, my concern is that the claim of being "fully analogue" is less clear in physical implementation. The mitigation requires engineered system–ancilla couplings that effectively realise tailored dissipators (see further comments below). Although the authors provide examples of how such dissipators could be implemented, it is not clear whether this can be regarded as fully analogue.

In continuation to this point, I am concerned that the practical implementation may blur the "deterministic" character of the proposal. Realising the required mitigation Lindbladian is hard, let alone it generally involves multi-qubit interactions between the system and ancillary degrees of freedom. In many experimental platforms, such engineered interactions are nontrivial and may themselves introduce additional noise channels or control errors.

More importantly, in the numerical examples, the mitigation dynamics is implemented via stochastic Hamiltonian evolution. It reintroduces stochasticity at the implementation level – which is not preferred in any sense. Thus, the physical realisation may both weaken the advantage over PEC and blur the distinction between a purely analogue scheme and one that relies on engineered control resources.

About the implementation and assumptions.

Another main concern is that the protocol requires accurate calibration not only of the noise rates but also of the noise operators themselves (i.e. the jump operators L_k). The mitigation Lindbladian is explicitly constructed from these operators, so any structural mischaracterisation of the noise will directly affect the cancellation mechanism. While this requirement is not unique to the present proposal — quasi-probabilistic error mitigation methods also rely on detailed noise characterisation — it remains a strong assumption in the analogue setting, where precise identification of the full Lindblad structure may be challenging.

In continuation, in comparison with quasi-probabilistic approaches, the system is coupled to an additional ancilla qubit, and the analysis assumes that ancilla errors can be modelled as a single-qubit Lindblad dissipator acting only on the ancilla, with rate Γ. At first glance, this appears to be a reasonable and standard modelling choice. However, upon closer inspection, this assumption may not always hold in practice.

In particular, ancilla errors may be induced by the engineered system–ancilla coupling itself and therefore need not be strictly local or independent of the system. Moreover, the analysis implicitly assumes:

1. No significant leakage out of the ancilla computational subspace;
2. Noise rates that are stationary or at least slowly varying so that they can be reliably estimated.

Indeed, this assumption on full Markovianity may not hold. For example, in solid-state computing, with partial swap with the reservoir, it can easily induce non-Markovianity. Moreover, this may not hold with additional stochasticity or coupling, see some recent discussions like emergence of non-Markovianity induced by either Pauli twirling, or reservoir entanglement.

Within this simplified model, the paper shows that ancilla noise contributes only an additional exponential decay term of the form -vΓW_01, which can be compensated through rescaling. Though I suspected, this conclusion may rely on the dissipative Lindblad structure.

A more problematic issue is the coherent (Hamiltonian) errors on the ancilla. A systematic ancilla detuning or unwanted rotation introduces additional Hamiltonian terms H_anc. Such coherent errors do not merely rescale the damping factor; instead, they can rotate the encoded off-diagonal block that carries the ideal state information. This may invalidate the simple recovery formula.

Overall this work is well written and I find the central idea conceptually stimulating. Despite the concerns outlined above, the work represents a meaningful contribution to the theory of error mitigation in analogue quantum simulation. The continuous-time perspective and the dissipative encoding mechanism are novel and likely to stimulate further discussion. I would like to

reconsider its publication if the authors address my concerns raised above. Alternatively, the authors may consider npj Quantum Information, which appears to be a suitable venue for this work.

Version 2:

Reviewer comments:

Reviewer #1

(Remarks to the Author)
I think the authors have addressed all the points in my previous reports. The main issue reported was the scaling of the sample complexity with system size. The authors have extended their results to show that while a naive application of the method could lead to exponential sampling overhead, one can utilize extra ancillas to alleviate this, and also that simpler types of simulation outputs tend to also alleviate the complexity substantially. This is a major improvement in my opinion.

My other comments were related to clarifying the relation between their proposal and previous work, in terms of the sampling complexity, but also in terms on how much knowledge of the noise channel is required. This has also been properly addressed in the new version.

Naturally, no method is perfect, and some limitations remain (e.g. engineering complicated Lindbladians). But I feel that the novelty of the method outweighs its limitations, particularly after the revised versions. I thus recommend publication in Nat Comms.

Reviewer #2

(Remarks to the Author)
Referee report

Continuous-time noise mitigation in analogue quantum simulation

I thank the authors for significantly modifying the manuscript. However, my overall judgment does not change; the hardware implementation requires an additional ancilla, which still may introduce additional errors. While I appreciate the additional simulation results, the system size is restricted to a small system with 4 spins, so it does not guarantee the feasibility of this scheme for larger systems.

Having said that, because this scheme is a novel class of quantum error mitigation, I recommend the publication of this paper in more specialized journals such as NPJ Quantum Information.

Reviewer #3

(Remarks to the Author)
The revised manuscript and response provide a much more thorough treatment of the theoretical and practical issues in their QEM method. I am pleased to see that the authors have carefully addressed nearly all my concerns.

I appreciate many of the new ideas and results presented. For example, I find the extension to non-Markovian noise particularly interesting. The authors have also added new results concerning the dependence of the factor “a” in the sampling overhead.

I have only one minor comment regarding the assumptions, which are the newly added discussion on the “Stochastic-Hamiltonian implementation.”

The authors focused on comparison between Trajectory-conditioned stochasticity (PEC approaches) and “Ensemble-averaged stochasticity (Our approach)”. The author said that “In other words, our scheme does not require any information about the stochastic trajectories, but only the final expectation value of the joint observable is required to reconstruct the ideal observables. ” and “Post-processing depends on per-shot measurement record”
However, I respectfully disagree on both points. I think PEC also does not require any information about the stochastic trajectories. I therefore do not see the difference in that perspective.

I would recommend the authors remove the arguments about the differences, unless there is other evidence or difference.

I would recommend its publication in Nature Communications.

Open Access This Peer Review File is licensed under a Creative Commons Attribution 4.0 International License, which permits use, sharing, adaptation, distribution and reproduction in any medium or format, as long as you give appropriate credit to the original author(s) and the source, provide a link to the Creative Commons license, and indicate if changes were made.
In cases where reviewers are anonymous, credit should be given to 'Anonymous Referee' and the source.
The images or other third party material in this Peer Review File are included in the article's Creative Commons license, unless indicated otherwise in a credit line to the material. If material is not included in the article's Creative Commons license and your intended use is not permitted by statutory regulation or exceeds the permitted use, you will need to obtain permission directly from the copyright holder.
To view a copy of this license, visit https://creativecommons.org/licenses/by/4.0/

List of changes

The following changes and revisions are noted in blue on the main text.

1. We have reframed the main novelty of the work as a fully autonomous error-mitigation protocol that preserves the continuous-time nature of analogue simulation. We removed the previous "discretisation" framing of Refs. [30, 31] and now compare the protocols in terms of their operational requirements: interleaved recovery pulses in Ref. [30], continuous jump-record monitoring in Ref. [31], and autonomous engineered dissipation in the present work.

2. We have expanded the sampling-overhead discussion. The revised manuscript relates the e^{4at} overhead to variance amplification in post-processing-based QEM, to the quasiprobability normalization overhead in PEC, and to worst-case sampling lower bounds for broad classes of QEM protocols. We also added a discussion of how a is evaluated for local, Pauli, and more general dissipators.

3. We have added a new subsection, "Noise model requirements," before the robustness analysis. This subsection states explicitly that the protocol requires a learned or physically motivated representation of the dominant jump operators and rates, relates this assumption to PEC and other model-assisted QEM methods, and discusses compatible noise-learning techniques.

4. We have expanded the "Physical implementation" section. The revised text clarifies the hardware requirements for engineering $L_k \otimes \sigma_z^{(a)}$, explains why the required mitigation strength is set by the native noise scale rather than by a parametrically larger corrective rate, and discusses relevant ingredients from engineered dissipation and erasure-conversion architectures.

5. We have strengthened the robustness analysis. Correlated system–ancilla noise is now explicitly described as a possible crosstalk effect and moved from the Supplementary Information (SI) to the "Methods" section. We also discuss rate errors, ancilla dephasing, coherent miscalibration, and the role of ancilla error-detection encodings.

6. We have added new numerical evidence for realistic implementation settings. In particular, the revised manuscript includes a four-qubit transverse-field Ising simulation with simultaneous engineered-rate error, ancilla dephasing, system–ancilla crosstalk, and coherent miscalibration, and SI contains a structural and Monte-Carlo comparison with Refs. [30, 31].

7. We have expanded the "Discussion" section to include the algorithm design and quantum computing perspectives on our approach, as suggested by the reviewers. The revised text explains how the off-diagonal block encoding of the ideal dynamics can be viewed as a continuous-time analogue of fault-tolerant gate synthesis and notes applications to adiabatic algorithms in the presence of moderate noise.

8. We have added a new author, Dr. Michael Hanks, for his critical contribution to the revision.

Reviewer 1

General Remarks:
This manuscript introduces a method for quantum error mitigation in continuous time. The topic is very timely. The development of quantum processors has followed a path in recent years where the number of qubits is not necessarily the main bottleneck, but rather their quality or fidelity is. That means that approaches where auxiliary qubits are used to mitigate errors are feasible in many experimental platforms. While naturally this is how quantum error correction works, such techniques typically demand very large overheads as well operating in discrete time, often introducing errors of its own. The proposal by the authors uses auxiliary qubits to correct errors in continuous time, and is thus tailored for analog quantum simulation.

Concretely, the method requires introducing an auxiliary system (which could be as simple as a single qubit) and engineering the dissipation of the new, extended system. Upon evolution, the noiseless expectation value of some operator A can be related to the expectation value of $A \otimes X$ in the extended system.

I think the method is clever and the fact that it is general and leads to exact noise cancellation makes it an noteworthy result. The main question mark is how feasible it would be to implement this in practice. In my view there are two main complications: engineering the dissipative dynamics, and getting around the sampling overhead. For the first aspect, in the most general case, engineering the dissipator of Eq. (3) could be quite challenging, if possible at all. For qubit systems, luckily, the problem of Lindbladian engineering is relatively studied. As the authors state, the method's complexity is greatly reduced if the dissipation is such that $\sum L^\dagger L \propto I$, which would happen for e.g. unital maps. This leads to a situation where the dissipation can be engineered by implementing a noisy Hamiltonian, as described in the section "Physical implementation". This scenario is relevant thus making their proposal appealing to a broad enough audience in the field.

The issue of the sampling overhead is addressed by the authors, however I have some concerns about this. The main one is about scaling with N, as I detail below.

The authors have done a good job in addressing other 'sources' of concern, namely how the protocol performs in presence of noise in the ancilla (beyond the engineered dissipation) and in the event of non perfect knowledge of the noise channels. I've found their treatment of these topics satisfactory, although I have a few comments and questions which will be detailed below. I've found it to be refreshing that the authors test their method not only in the problem of obtaining expectation values of macroscopic observables (which tend to be inherently robust or relatively predictable in presence of dissipation) but also more complicated quantities like the Loschmidt echo and the spectral response.

Our response: We thank Reviewer 1 for their careful consideration and suggestions. We are pleased to hear that the reviewer evaluated our method as "clever" and our results as "noteworthy." We address the reviewer's points in detail below.

Major Comment 1:
Does a scale with N? From the example of the Heisenberg model, this seems to be the case. If so, the authors should address this point. Does this mean that the sampling overhead scales exponentially with N?

Our response: Strictly speaking, this depends on the noise model and the target observable. For the examples considered in our paper, a *naive* application of the protocol incurs a to increase with N, in which case overhead scales exponentially in the system size. However, the reviewer's comment inspired us to develop a more efficient protocol using a multi-flag ancilla register, which significantly reduces the sample complexity from exponential scaling to a weight-resolved overhead corresponding to the target observable. Remarkably, for local linear observables, such as magnetization, two-point correlators, and energy density, which are commonly measured in analogue quantum simulations, this yields *sampling overhead independent of N, dramatically improving the practicality of our protocol for larger system sizes* (see also Fig. 1).

We first provide a constructive proof of additivity for IID local dissipators, then explain why this worst-case scaling is *not* the relevant one for typical analogue-simulation observables.

(a) Constructive proof. We note that $L^\dagger L$ must be positive semi-definite. Suppose we have Lindblad operators L_{S,j_S}, where the indices in the set S denote all subsystems on which the dissipator acts non-trivially and the index j_S labels the distinct Lindblad dissipators for each distinct set S. For any two operators $L_{S,j}$, $L_{S',k}$ for which $S \cap S' = \emptyset$, we have the equality

$$\lambda_{\max}\left(L_{S,j}^\dagger L_{S,j} + L_{S',k}^\dagger L_{S',k}\right) = \lambda_{\max}\left(L_{S,j}^\dagger L_{S,j}\right) + \lambda_{\max}\left(L_{S',k}^\dagger L_{S',k}\right). \quad (1)$$

For dissipators applied uniformly on a per-qubit basis, this gives $a_N = N a_1$.

(b) Observable-specific overhead. This worst-case scaling is, however, not the relevant one for typical analogue-simulation observables. The noisy Lindbladian decomposes into Schur-irrep blocks Π_μ on which $\sum_k L_k^\dagger L_k$ has eigenvalue λ_μ, and the recovered variance of an observable supported on block μ scales as $e^{4\lambda_\mu t}$, not as $e^{4a_N t}$. As an intuitive example, a noninteracting Hamiltonian with a macroscopic linear observable, e.g., total magnetization $\sum_{i=1}^N \sigma_z^{(i)}$, admits a *system-size-independent* sampling overhead of $e^{4a_1 t}$ by using independent ancilla qubits per noise channel. This is a manifestation of the universal weight-resolved bound of [Mele et al., Nat. Phys. 22:751 (2026); Quek et al., Nat. Phys. 20:1648 (2024)], saturated here by an explicit protocol. For a general observable A under a locally interacting Hamiltonian, the overhead scales as $e^{4a_1|C(A,t)|t}$, where $|C(A,t)|$ is the size of the Heisenberg light cone defining the effective region of the dynamics associated with A, which is determined by the Lieb–Robinson bound.

(c) Block-resolved heralding. The weight-resolved bound is realised by an explicit construction: a multi-flag ancilla register with one ancilla per noise irrep (r ancillas total, $r = O(N)$ for IID local noise) replaces the single-qubit heralding flag of the bare protocol. The engineered coupling $L_k \otimes \sigma_z^{(\mu_k)}$ on the ancilla matched to the firing irrep μ_k produces a master-equation projector-collapse on the joint state at the per-block rate λ_μ, and the recovered observable's overhead drops from $e^{4a_S T}$ to $e^{4\lambda_{\mu_A} T}$, which is independent of N for local observables. The construction is verified numerically, and the predicted gain at $\gamma T = 0.3$ is shown in the following Fig. 1.

Crossover-N caveat for the multi-flag construction. The multi-flag construction requires $O(N^2)$ ancilla–system couplings, each carrying a residual crosstalk of relative strength σ_X (typically 10^{-2}–10^{-3} in current cavity QED). Crosstalk-induced bias in the recovered observable

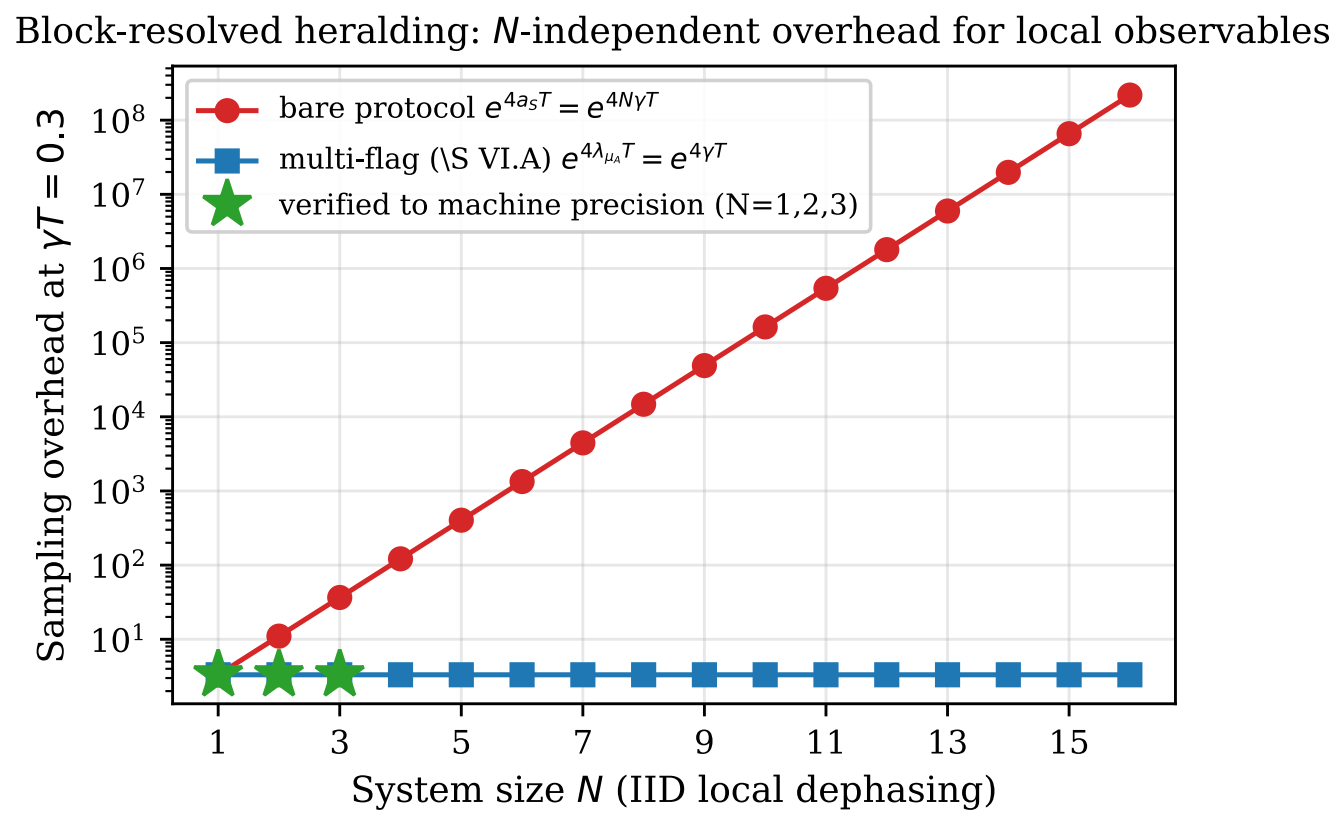

Figure 1: Sampling-overhead reduction of the multi-flag construction for IID local dephasing on N-qubit chains, recovered observable X_1 ($w = 1$). The bare-protocol overhead $e^{4N\gamma T}$ grows exponentially in N; the multi-flag overhead $e^{4\gamma T}$ is flat. Star markers are direct numerical verifications at machine precision.

scales as $\sim N^2\sigma_X^2\gamma T$; the multi-flag scheme is preferred over the bare scheme so long as this bias remains below the bare-scheme variance reduction $e^{-4(N-1)\gamma T}$, giving a hardware-set crossover $N^\star \sim \frac{1}{2\gamma T}\ln(1/\sigma_X^2\gamma T) - O(\ln N)$. For $\sigma_X = 10^{-2}$ and $\gamma T = 0.3$ this is $N^\star = O(10)$; for $\sigma_X = 10^{-3}$, $N^\star = O(20)$. The multi-flag construction provides an exponentially-improving local-observable advantage *up to a hardware-set crossover*, with a clear path to higher $N^\star$ via reduction of crosstalk.

To address these points, we have revised the manuscript as follows:

1. We have added the following discussion in the "Sampling overhead" section:
   "*For an N-qubit system subject to local noise on each qubit, a naive application of the proposed mitigation protocol incurs a sampling overhead of e^{4Na_1t}, where a_1 is the rescaling factor for the single-qubit case. Nevertheless, this overhead can be significantly reduced for a local observable supported on a block with small Pauli weight by introducing multi-flag ancillary qubits, in which case the weight-resolved scaling of the sampling overhead applies. One can further decompose the noisy Lindbladian into Schur-irreducible blocks, and projecting onto these blocks yields an even lower sample complexity (see Methods and SI for details).*"

2. In Methods, we have added discussions on the improved bound for observable-specific sampling overhead along with its numerical simulation of Fig. 1 (Fig. 5 of the revised manuscript).

3. In the revised SI, we have provided a more detailed derivation of the weight-resolved bound using irreducible representation and explicitly constructed "Block-resolved heralding via a multi-flag ancilla register" that realises the improved sampling overhead.

Major Comment 2:
In the discussion regarding sampling overhead, it is stated that sampling overhead scaling exponentially with noise rate and time is "expected from all QEM protocols". I think the authors should expand on this point – what is the intuition behind it? what are other QEM protocols which share this feature, and what are the references to previous studies?

Our response: We thank the reviewer for this helpful comment. After reading through the reviewer's comment, we realised that the original phrase "expected from all QEM protocols" was too broad.

Nevertheless, the intuition behind this exponential scaling remains valid because post-processing-based QEM suppresses noise-induced bias by statistically inverting, reweighting, or extrapolating the noisy data. This bias reduction generally comes at the cost of amplified estimator variance. In our protocol, the mitigated estimator is multiplied by the normalization factor e^{2at}, and therefore, assuming comparable per-shot variances, the sampling overhead scales as e^{4at}. This is directly analogous to probabilistic error cancellation, where the sampling overhead is given by the square of the quasiprobability normalization factor. Since this factor multiplies over noisy circuit locations, PEC can incur a sampling cost exponential in the accumulated noise strength [Cai et al., Rev. Mod. Phys. 95:045005 (2023); Temme-Bravyi-Gambetta, Phys. Rev. Lett. 119:180509 (2017)]. More generally, sampling lower bounds show that broad classes of QEM protocols require exponential resources in worst-case settings [Takagi–Endo–Minagawa–Gu, npj QI 8:114 (2022); Takagi–Tajima–Gu, Phys. Rev. Lett. 131:210602 (2023); Quek et al., Nat. Phys. 20:1648 (2024)]

> In response to the reviewer's comment, we have replaced the original sentence with the following more qualified statement as "*This is consistent with the general QEM tradeoff that suppressing bias by post-processing typically amplifies estimator variance, where exponential sampling costs with respect to the evolution time t are known explicitly for probabilistic error cancellation and are also implied, in worst-case settings, by general sampling lower bounds for broad classes of QEM protocols*" with relevant references.

Major Comment 3:
Related to this point, the proposed protocol assumes knowledge of $D[\rho]$, the dissipator. I think the paper would be strengthened by emphasizing this issue earlier on (before the robustness analysis). How does this requirement relate to the wider QEM method zoo (where such knowledge is also required, to more or less extent), and ideally also connecting it to noise learning methods from which this approach could benefit.

Our response: As the reviewer correctly pointed out, our protocol requires the knowledge of the dissipator, more precisely, jump operators L_k to be mitigated. Nevertheless, the need for noise information is not unique to our protocol: probabilistic error cancellation requires a characterization of the noisy operation and a representation of its inverse, while Pauli-Lindblad versions of PEC explicitly learn sparse Lindblad generators and rates [van den Berg et al., Nat. Phys. 19:1116 (2023)]. Zero-noise extrapolation is less model-specific, but still relies on an experimentally controllable noise-scaling procedure and an extrapolation ansatz.

We also emphasize that the required information for the noise-model characterization is compatible with existing noise-learning methods. General Lindblad operators can be characterized via Lindblad or master-equation reconstruction protocols demonstrated in NMR [Boulant et al., Phys. Rev. A 67:042322 (2003)], trapped ions [E. Ben Av et al., Phys. Rev. A 101:062305 (2020)], and superconducting processors [Samach et al., Phys. Rev. Appl. 18:064056 (2022)]. For Pauli or Pauli-twirled noise, randomized compiling [Wallman-Emerson, Phys. Rev. A 94:052325 (2016)], cycle benchmarking [Erhard et al., Nat. Commun. 10:5347 (2019)], and sparse Pauli-Lindblad learning [van den Berg et al., Nat. Phys. 19:1116 (2023); Quantum 8:1556 (2024)] provide scalable routes to the relevant Pauli generators and rates. Recent compressed-sensing [Dobrynin et al., Quantum Sci. Tech. 10:045041 (2025)] and shadow-based [Birke et al., arXiv:2602.14694] Lindblad tomography methods further reduce the characterization cost under locality or sparsity assumptions.

We have added the subsection "Noise model requirements" to the revised manuscript to discuss the required knowledge of the dissipator in the broader QEM landscape and to describe feasible approaches for characterizing it, as follows:

"*The protocol requires a learned or physically motivated representation of the dominant jump operators L_k. This requirement is analogous to the noise-characterisation assumptions in other QEM methods. For example, PEC requires a characterised noise map in order to construct an inverse quasiprobability representation, and recent large-scale PEC implementations learn sparse Pauli-Lindblad noise models for this purpose. This can be done by master-equation reconstruction protocols, which have been demonstrated experimentally in NMR, trapped-ion, and superconducting-qubit platforms. When the relevant dissipator is sparse, local, or Pauli-twirled, the required information can be obtained by established characterisation methods such as randomized compiling, cycle benchmarking, and sparse Pauli-Lindblad learning. This can be further combined with recently proposed compressed-sensing or shadow-based Lindblad tomography.*"

We have also relocated the subsection "Protocol robustness against imperfect noise characterization" to appear before "Protocol robustness against ancilla noise" for better logical flow.

Major Comment 4:
Is the calculation of 'a' potentially a bottleneck of the model? In the example described this is done explicitly for $N = 4$ spins, but the case is relatively trivial and seems to extend ok to other N (although it might lead to an unwanted scaling of a with N).

Our response: The ease of calculation depends on the noise model. As mentioned in our response to **Major Comment 1** above, for dissipators acting locally on distinct subsystems, the calculation is almost trivial, and a is the sum of the various a_ℓ's on the ℓ-th local subsystems, i.e., $a = \sum_\ell a_\ell$ can be easily evaluated. Especially for Pauli dissipators such that $L_k^\dagger L_k = \gamma_k \mathbb{I}$ with rate γ_k, we have even simpler formula, $a = \sum_k \gamma_k$. For non-local dissipators, finding the largest eigenvalue of the component dissipators is equivalent to a ground-state calculation on $\sum_k L_k^\dagger L_k$, aided by symmetry assumptions where they apply. Therefore, for the most physically relevant cases, where the dissipators are local or well-structured, the calculation of a does not induce computational challenge.

We also note that, as discussed in **Major Comment 1**, the linear scaling of a with respect to N does not necessarily reflect the exponential sampling overhead for specific target observables, such as single-site magnetisations, two-point correlators, and energy density, when adopting a multi-flag ancilla register.

> We have added additional discussion on evaluating the value of a in the revised "Noise mitigation protocol" session as follows:
>
> "*For dissipators acting locally on distinct subsystems, a can be directly evaluated as the sum of the various a_ℓ's on the ℓ-th local subsystems, $a = \sum_\ell a_\ell$. Especially for dissipators satisfying $L_k^\dagger L_k = \gamma_k \mathbb{I}$ (e.g., Pauli operators) with rate γ_k, we have an even simpler formula, $a = \sum_k \gamma_k$. For non-local dissipators, finding the largest eigenvalue of the component dissipators is equivalent to a ground-state calculation on $\sum_k L_k^\dagger L_k$, aided by symmetry assumptions where they apply. For the most physically relevant cases, the dissipators are local or well-structured, so that the calculation of a does not induce computational challenge.*"

Minor Comment 1:
The authors seem to focus on quantum simulation as an application, but it seems their results could be applicable also to adiabatic quantum computing. Is this correct or do you envision specific hurdles in this?

Our response: We thank the reviewer for the insightful comment. As the reviewer correctly pointed out, the protocol applies to adiabatic quantum computation without any modification. Nevertheless, claims of computational advantage should be treated with greater care, as the sample complexity typically scales exponentially with time, as we discussed in **Major Comment 2**.

> The following sentence has been added to the Discussion section stating the protocol's applicability to adiabatic quantum computing as: "*Our protocol could be directly applied to improve adiabatic quantum computing in the presence of moderate noise, and, ...*".

Minor Comment 2:
In page 2, at the beginning of the 'Noise mitigation protocol' section it says that the Hamiltonian can be time-dependent, but in the previous paragraph the contrary seems to be stated. Which one is correct?

Our response: The mitigation Lindbladian is independent of $H(t)$, so time-dependent system Hamiltonians are accommodated without modification. The analysis in "Framwork," can be generalised by replacing $e^{-iHt/\hbar}$ with the corresponding time-ordered exponential $\mathcal{T}\left[e^{-(i/\hbar)\int_0^t H(\tau)d\tau}\right]$; the off-diagonal-block argument is unchanged.

> We have removed the contradictory wording from the "Framework" section and added a footnote when mentioning the time-dependent generalisation in the "Noise mitigation" section to make this explicit as "*As the mitigation Lindbladian is independent of $H(t)$, our protocol can be generalised by replacing $e^{-iHt/\hbar}$ with the corresponding time-ordered exponential $\mathcal{T}\left[e^{-(i/\hbar)\int_0^t H(\tau)d\tau}\right]$ for a time-dependent system Hamiltonian $H(t)$ without modification.*"

Concluding Remarks:
In summary, the paper is well-written, most ideas are presented in a clear way, and the analysis is compelling, with the caveat of the scaling with N. The results are noteworthy and important, and have the potential to impact future experiments. The weakness I've found is that the paper lacks a more detailed analysis and comparison of the cost or complexity of this method as compared to other QEM approaches.

Our response: We appreciate the reviewer's positive evaluation of our work. We believe that the revised manuscript sufficiently addresses the reviewer's concerns so that it is now suitable for publication in *Nature Communications.*

Reviewer 2

General Remarks:
This paper discuss the QEM protocol for analog simulator. The proposed method is as follows. They propose to prepare the ancilla plus state and design the Lindblad operator in the composite system of ancilla+system such that the $\mathrm{Tr}\left[[A \otimes \sigma]_x \, \rho_{com}\right]$ (ρ_{com} is the density matrix in the composite system) uniformly shrinks compared with the ideal result with the $e^{(2at)}$ (Eq. (7)) with a being a constant determined by the Lindbladian. Then, by rescaling the result by $e^{(2at)}$, I think the idea is interesting.

Our response: We thank the reviewer for their close reading of our manuscript, and for prompting us to better highlight the significance of our work, the connection to prior continuous-time QEM proposals, and to compare numerically against those proposals in non-trivial settings. We address their points in detail below.

Major Comment 1: Mischaracterisation of prior continuous-time QEM (Refs [30, 31])
Nevertheless, I think the novelty of this work does not reach the novelty and practicality expected in nature communications. First, the novelty should lie in the their protocol is truly the first continuous QEM protocol without discretization. However, this is based on the author's misunderstanding of the previous work. To my knowledge, Refs. [30] and [31] do not assume discretization of the process. For example, Ref [30] first discretize the continuous process to use the idea of the original PEC first, but they clearly show that the limit of $\delta t \to 0$ can be taken by using the stochastic approach which determines the QEM jump operation stochastically (See Sec IV and V in Ref. [30]). Although it is true that this protocol requires interleaving single-qubit pulse operations and some commutation error with the original Hamiltonian may occur when switching off the original Hamiltonian is not possible, there is no error due to discretization. It is not ideal to base the novelty claim on an inaccurate characterization of the contributions of prior works. At least, the authors should clarify what is truly continuous error mitigation from the mathematical level. I guess the introduction and framework section need to be changed significantly to have the clear comparison with the prior works. The authors need to thoroughly compare their method with the prior methods numerically, by correctly understanding simulating the prior methods. The same authors seem to have a similar discussion in their prior work. `https://journals.aps.org/pra/abstract/10.1103/PhysRevA.109.012431`

Our response: The reviewer is correct that the previous statement – contrasting our protocol with Refs [30, 31] in terms of "discretisation" – was imprecise. Both references take the $\delta t \to 0$ limit at the master-equation level, as the reviewer correctly pointed out. In particular, Ref. [30] explicitly notes that its stochastic recovery operations "can be pre-engineered into the original evolution via a noisy time evolution of a modified Hamiltonian," so that there is no error due to discretization. Therefore, we have removed the "discretisation" framing from the introduction and framework sections.

In the revised manuscript, we have reframed the novelty of our approach as a *fully autonomous error-mitigation protocol* that does not require interleaved pulse operations, as in Ref. [30], or continuous monitoring of measurement records, as in Ref. [31]. This feature makes our protocol more

naturally suited to analogue simulation frameworks, where the preferred experimental procedure is to perform measurements only at the final time, without any intermediate control operations or measurements during the evolution. In the following, we highlight the key points of comparison between our approach and those of Refs. [30, 31]:

Comparison with Ref. [30]: We provide a more detailed comparison between our work and Ref. [30] in terms of the strength of the mitigation operation. More precisely, in Ref. [30], recovery is implemented by a finite-time operation

$$\mathcal{B}_j = e^{-iH_{\mathrm{kick}}\tau_{\mathrm{kick}}},$$

which is applied at stochastic jump times drawn from a Poisson process. To obtain the ideal continuum limit, a fixed recovery rotation must be implemented in an instantaneous-pulse limit, $\tau_{\mathrm{kick}} \to 0$. This requires $\|H_{\mathrm{kick}}\| \to \infty$. With a bounded physical drive, $\|H_{\mathrm{drive}}\| \le H_{\mathrm{max}}$, a rotation by angle θ_j instead requires a finite duration

$$\tau_{\mathrm{kick}} \gtrsim \theta_j / H_{\mathrm{max}}.$$

During this finite pulse window, the background simulator Hamiltonian continues to act and produces a residual error. Ref. [30] explicitly identifies a per-pulse residual of order $\mathcal{O}(\delta t^3)$ and requires the kick intensity to be sufficiently large compared with the simulator Hamiltonian. For a Poisson record with mean number of pulses $N_{\mathrm{pulse}} = \Gamma T$, these residuals accumulate, so keeping the total bias below a fixed target requires increasing drive amplitude or bandwidth as the sharp-kick limit is approached.

Thus, the "sufficiently strong kick" condition in Ref. [30] is the finite-bandwidth counterpart of our statement that the unbiased impulsive protocol requires unbounded drive strength in the instantaneous-kick limit. One may instead run such a protocol at finite pulse duration, as in practical dynamical-decoupling implementations, but then a residual bias remains due to background evolution during the pulses, and this bias is reduced only by increasing the available drive strength.

Our proposal avoids this particular pulse-bandwidth tradeoff because it does not rely on instantaneous recovery kicks. The mitigation is generated by a bounded-norm, time-independent engineered Lindbladian, so the continuum limit is not obtained by sending a pulse duration to zero or a drive amplitude to infinity. We also note that the required mitigation strength is set by the physical noise channel being mitigated: for a jump operator L_k, the corresponding mitigation jump $L_{\mathrm{mit}} = L_k \otimes \sigma_z$ has the same operator-norm scaling, $\|L_{\mathrm{mit}}\| = \|L_k \otimes \sigma_z\| = \|L_k\|$. Thus, the engineered dissipation is not required to be parametrically stronger than the native noise rate (see also our response to **Major Comment 2**).

Finally, we have added a clarification regarding the relation to the pre-engineered-Hamiltonian variant discussed in Ref. [30]. That construction addresses the impulsive protocol in a different implementation setting, while still retaining recovery operations associated with pre-sampled jump times and the corresponding signed-record bookkeeping. Our proposal is complementary: it aims to replace the trajectory-dependent recovery step by a time-independent engineered dissipative coupling, thereby realizing the mitigation dynamics in an autonomous continuous-time form.

Comparison with Ref. [31]: We have clarified the distinction between the influence-martingale construction of Ref. [31] and the autonomous implementation proposed in our work. In Ref. [31],

the mitigation factor

$$\mu(t) = \prod_k \frac{r_{j_k}}{\Gamma_{j_k}} \exp\left[-\int_0^t (\Gamma' - \Gamma)\, dt'\right]$$

is a trajectory-dependent classical weight. Evaluating it requires knowing which quantum jumps occurred and when they occurred, and the measured click record must then be classically post-processed on a shot-by-shot basis. Thus, the click record is an essential input of the protocol, not merely a bookkeeping device that can generally be absorbed into autonomous quantum dynamics. The ancilla-based construction cited in Ref. [31] provides a way to simulate the required jump trajectories, but the ancilla still has to be monitored and the resulting record is still used to construct the classical influence weight.

By contrast, our scheme attempts to engineer a special signed-erasure structure on an enlarged system–ancilla Hilbert space, instead of implementing a generic influence martingale. The recovery jump $L_k \otimes \sigma_z^{(a)}$ maps each relevant system jump to a parity flip of the ancilla. As a result, the generic trajectory-dependent weight collapses to a deterministic magnitude, e^{2aT}. This leads to the advantage of our construction that the required sign information is stored coherently in an ancilla parity and extracted at the end, instead of being reconstructed from a continuously monitored jump record.

More technical analysis on sample-complexity scaling, heavy-tail behaviours of Ref. [31] in Monte-Carlo unravelling, and robustness against coherent-error have been also discussed in the revised SI (see also our response to **Major Comment 2**).

We thank the reviewer again for pointing out the mischaracterisation of the previous manuscript and for their insightful comments, which have enabled us to reconsider and clarify the novelty of our work.

In response to the reviewer's comment,

- We have rewritten the introduction and framework sections to remove the "discretisation" framing.
- We have reframed the novelty of our approach as a fully autonomous time-continuous error-mitigation protocol by revising the sentence in the Introduction as follows:
"*In this work, we introduce a fully <u>autonomous</u> time-continuous error cancellation protocol designed specifically for analogue quantum simulators. Our method is native to the analogue setting, as it preserves the system's continuous-time evolution <u>without any interleaving pulse operations or intermediate measurements</u> by operating entirely within the framework of Markovian open system dynamics, described by the Lindblad master equation..*"
- In Methods, we have added a subsection to more precisely compare our approach with Refs. [30, 31], while providing a more detailed analysis in SI.

Major Comment 2: Practicality of multi-body engineered Lindbladians under noise. In addition, while I think the proposed method seems novel, I doubt the practicality of their method. This method needs to design the complicated Lindblad operator, such as $[\sigma_A^z \otimes \sigma_B^z]$ for mitigating dephasing errors in the system. However, to design such Lindblad operators, we need another ancilla system and require three-body interaction. This is in principle possible, but the expected error to realize Lindblad operators is significant. The effect of crosstalk to the system is not negligible. The aim of error mitigation is to mitigate errors with easy operations, so this seems a bit contradiction to me. At least, the authors need to numerically simulate the realistic noise model and compare their result with methods in Refs. [30, 31] with respect to the sampling overhead and the feasibility and runtime of QEM routine.

Our response: As the reviewer pointed out, multi-body engineered Lindbladians, e.g., $\sigma_A^z \otimes \sigma_B^z$, could be a major technical challenge for realizing the proposed protocol. Nevertheless, the required hardware ingredient is the dispersive ancilla–system coupling already deployed for cat-code stabilization in current cavity-QED experiments [Lescanne et al., Nat. Phys. 16:509 (2020); Touzard et al., PRX 8:021005 (2018)], whose crosstalk is 10^{-2}–10^{-3} smaller than the engineered Lindbladian rate. Since this can be viewed as a continuous-time signed erasure channel, our protocol belongs to a family whose hardware viability has been independently established by erasure-converted superconducting and neutral-atom qubits [Kubica et al. 2023; Teoh et al. 2023; Levine et al. 2024; Sahay et al. 2023].

More importantly, we highlight that our protocol *requires only a weak coupling dissipation strength*, which scales with the noise rate $\|L_{\text{mit}}\| = \|L_k \otimes \sigma_z\| \sim \|L_k\|$. This is significantly smaller compared to other dissipation engineering approaches for suppressing noise, e.g., Autonomous quantum error correction, where the engineered dissipation strength should be significantly stronger than the noise rate. We have now highlighted this point in the revised Introduction and Physical Implementation sections.

We also remind the reviewer that correlated system-ancilla noise was already discussed in the previous submission in SI (Sec. II), but only mentioned briefly in the main manuscript without relating it to the crosstalk effect. In the revised manuscript, we have now explicitly explained the origin of the correlated system-ancilla noise as a crosstalk effect and moved the section in SI to Methods, with a more detailed discussion.

Finally, following the reviewer's suggestion, we have now provided more rigorous numerical simulation results by incorporating all these noise effects to reflect more realistic experimental scenarios (see also our response to **Reviewer 3**'s **Major Comments 5 & 6** regarding non-Markovianity effects and coherent ancilla errors). In particular, we now demonstrate how the bias of the target observable can be broken down into distinct noise effects (see Fig. 2). This, along with the Monte-Carlo unravelling of all three schemes, leads to a structural feasibility comparison between our protocol and the approaches in Refs. [30, 31], which have been added to the revised SI. Here, we summarize the key properties in the following table with several important remarks:

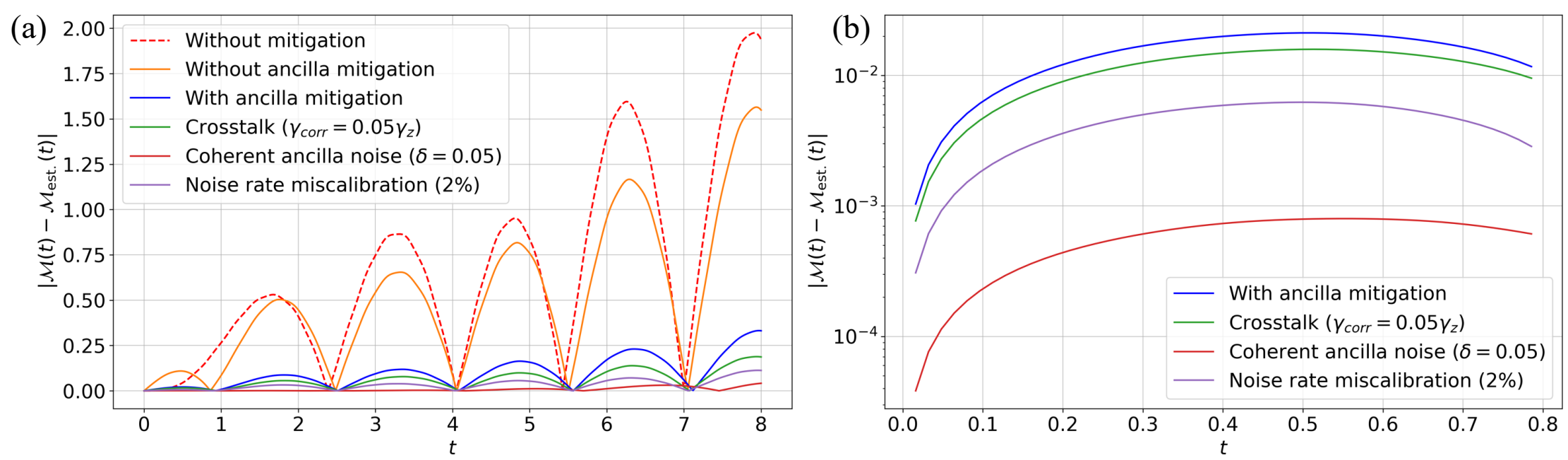

Figure 2: Realistic noise effects and bias breakdown for the 2×2 two-dimensional anisotropic Heisenberg model simulation studied in the main text.

	**Sun et al. [30]**	**Donvil–Muratore-Ginanneschi [31]**	**This work**
Generator class	quasi-Lindblad (signed) on system	quasi-Lindblad (martingale) on system	**CPTP on joint S+A**
Estimator tail class	sub-Gaussian, bounded	heavy-tailed (Bernstein-only)	sub-Gaussian, bounded
Concentration regime	Hoeffding	Bernstein only	Hoeffding
Per-shot classical record	$O(\lambda T)$ recovery labels	full quantum-jump trajectory	none
Classical post-processing per shot	$O(\lambda T)$ multiplications (sign accumulation)	full trajectory record + martingale product	one global rescaling
Wall-clock overhead vs unmitigated	runtime $\times$ sample count $e^{4\lambda T}$	runtime $\times$ sample count $e^{4\lambda T}$ + per-shot trajectory I/O	runtime $\times$ sample count $e^{4\lambda T}$
Single-qubit-control sites	every noisy system qubit	every noisy system qubit	ancilla register only
Mid-circuit measurement	not required	required (or designed reservoir)	not required
Sampling-overhead, global obs.	$e^{4a_S T}$	$e^{4a_S T}$	$e^{4a_S T}$
Sampling-overhead, weight-w local obs.	$e^{4\lambda_1 \lvert C(A_S,T)\rvert T}$ *(via lightcone-restricted recovery)*	$e^{4\lambda_1 \lvert C(A_S,T)\rvert T}$ *(via lightcone-restricted unravelling)*	$\boldsymbol{e^{4w\lambda_1 T}}$ *(multi-flag, Sec. III in SI)*
Coherent-error bias	implicit $O(\delta^2)$ on system	implicit $O(\delta^2)$ on system	$O(\delta^2)$ on ancilla, $\sim 11\times$ prefactor reduction with distance-2 detection
Protection register	system register	system register	ancilla register

- Operational simplification and local-observable advantage: Unlike Refs. [30,31], the proposed protocol localizes individual control to the ancilla rather than the system qubits. For local observables, the multi-flag ancilla construction reduces the sampling exponent from a lightcone-dependent rate to the per-block rate $w\lambda_1$, giving a polynomial-in-T advantage over lightcone-restricted Ref. [30].
- Numerical validation and statistical comparison: Simulations show near-exact recovery for correlated two-body noise and site-local dephasing using a single shared ancilla. For global observables, the proposed scheme and Ref. [30] have equivalent bounded-shot statistics, while Ref. [31] exhibits heavy-tailed estimator weights and substantially worse concentration.
- Realistic-noise robustness and hardware caveat: A 4-qubit realistic-noise simulation shows a significant bias reduction even with stacked rate errors, ancilla dephasing, crosstalk, and coherent miscalibration. The main remaining challenge is scaling many simultaneous system–ancilla couplings while maintaining low crosstalk, with cold-atom and Rydberg platforms identified as promising hosts.

In response to the reviewer's comments, we have revised the manuscript as follows:

- "Physical Implementation" section has been expanded to provide a more detailed and experimentally relevant consideration of the proposed protocol. We have provided a more detailed discussion on the main physical ingredients of dissipation engineering and their feasibility as

  "*While this requires multi-qubit interactions between system and ancillary states, main ingredients for the proposed dissipation engineering have recently been realized in trapped-ion, quantum dot, and cavity QED systems. Alternatively, by interpreting our ancilla-assisted recovery dynamics as a continuous-time signed erasure channel (see SI for details), our protocol belongs to a family whose hardware viability has been independently established by erasure-converted superconducting and neutral-atom qubits.*"

  and also highlighted the requirement of only a weak dissipation strength as

  "*We also highlight that only a weakly coupled dissipation strength is required, which scales with the noise rate* $\|L_{\text{mit}}\| = \|L_k \otimes \sigma_z\| \sim \|L_k\|$. *This is significantly smaller compared to other dissipation engineering approaches for suppressing noise, e.g., in Refs. [57,58], where the engineered dissipation strength should be significantly stronger than the noise rate.*"
- We add Sec. VI in SI to provide a more rigorous numerical simulation, including the breakdown of the bias induced by different sources of noise.

Concluding Remarks:
Overall, I think the proposal is interesting, and I like the idea. I suppose their method may be more useful for algorithm design than error mitigation. I suppose the authors have a misunderstanding about the prior works, and doubt the practicality. I recommend the authors transfer the paper to more specialized journal after addressing my comments.

Our response: We are grateful to the reviewer for their careful and constructive feedback, which has substantially helped us improve the manuscript. However, we respectfully disagree that the work is better suited to a specialised venue. The protocol applies to any analogue quantum simulator described by a Lindbladian noise model and is independent of the specific system Hamiltonian. Thanks to the reviewer's comment, we have now clarified the novelty of our results compared to the advantages over prior continuous-time protocols. We have also provided more rigorous numerical evidence of the feasibility of the protocol by identifying essential elements and noise levels that can be achievable by near-term devices. We believe that this combination of generality and broad applicability in analogue quantum simulation, now emphasized in the revised manuscript, aligns well with the cross-disciplinary criteria of the journal.

We also appreciate the reviewer's comment on the usage of our approach for algorithm design. This is a perceptive observation, as the proposed protocol's structure of engineered Lindbladian on system + ancilla with the ideal dynamics encoded in the off-diagonal block of the joint state, could be utilized as a continuous-time analogue of fault-tolerant gate synthesis [Bravyi–Kitaev, Phys. Rev. A 71:022316 (2005); Litinski, Quantum 3:128 (2019)].

- We have added the following sentence in the Discussions: "*Our protocol could be … its ancilla-assisted noise-protection formalism could be utilized for a continuous-time analogue of fault-tolerant gate synthesis.*" along with relevant references.

Reviewer 3

General Remarks:
This work proposes a continuous-time error mitigation protocol for analogue quantum simulators based on engineered dissipative coupling to ancillas. By embedding the system into a larger Hilbert space and carefully constructing joint Lindblad operators, the authors show that the system's noise can be cancelled in the off-diagonal blocks of the joint density matrix. This central idea is simple and elegant. As such, the ideal noiseless evolution may be then recovered via measurement and classical post-processing.

I like this idea which avoids discretization and physically implementing a "negative dissipator" like stochastic error mitigation. The illustrative examples demonstrate feasibility under simplified conditions. Though, I have a few concerns on the assumptions, schemes and the claimed advantages as outlined in the following.

Our response: We thank Reviewer 3 for their careful reading of our manuscript and for prompting us to better highlight the significance of our work. The reviewer's concern about coherent ancilla errors in particular led us to a stronger result than the reviewer anticipated, and to a constructive scheme for amplifying it. We address their points in detail below.

Major Comment 1: Advantages and distinction to PEC and analogue simulation.
At the level of the Lindbladian description, the scheme is deterministic and does not rely on quasi-probability sampling, which is the main conceptual innovation of this work. However, my concern is that the claim of being "fully analogue" is less clear in physical implementation. The mitigation requires engineered system–ancilla couplings that effectively realise tailored dissipators (see further comments below). Although the authors provide examples of how such dissipators could be implemented, it is not clear whether this can be regarded as fully analogue.

Our response: The reviewer correctly identifies that the protocol is *deterministic* and does not rely on quasiprobability sampling. We also agree with the reviewer that the phrase "fully analogue" could be ambiguous when discussing the physical implementation of the protocol.

To clarify the advantage of our approach and its distinction from PEC, we have replaced the phrase "fully analogue" with "fully preserves the continuous-time nature of the dynamics." This is based on the fact that our protocol does not require impulsive recovery operations, which in principle require infinitesimal pulse durations, nor does it require continuous monitoring of quantum jumps, which induces discontinuous state updates. These points are now emphasized throughout the manuscript to more clearly describe the novelty of our results (see also our response to **Reviewer 2**'s **Major Comment 1**).

Following the reviewer's comment,

- We have revised the abstract by replacing the phrase "fully analogue" with the following statement: *Our protocol fully preserves the continuous-time nature by avoiding any active control or measurement of the system during the dynamics, while achieving exact noise cancellation.*"
- Throughout the manuscript, we have highlighted the main feature of our protocol: it does not require interrupting the evolution with either strong pulse operations or measurements.

Major Comment 2: Multi-qubit Lindbladians introduce additional noise channels. In continuation to this point, I am concerned that the practical implementation may blur the "deterministic" character of the proposal. Realising the required mitigation Lindbladian is hard, let alone it generally involves multi-qubit interactions between the system and ancillary degrees of freedom. In many experimental platforms, such engineered interactions are nontrivial and may themselves introduce additional noise channels or control errors.

Our response: We agree with the reviewer that realizing the mitigation Lindbladian is a nontrivial hardware requirement. We have revised the manuscript to make this assumption more explicit in the "Physical Implementation" section and to clarify how additional noise channels and control errors should be treated.

We clarify what we mean by the "deterministic" character of the proposal. The protocol is deterministic in the sense that, once the engineered dissipator is implemented, the mitigation is generated by a fixed continuous Lindbladian evolution and does not require stochastic quasiprobability sampling, measurement-conditioned feedback, or postselection on individual trajectories.

We also note that the required system–ancilla coupling is closely related to dispersive and conditional couplings already used in cat-code stabilisation [Lescanne et al., Nat. Phys. 16:509 (2020)] and in Schrödinger-cat cavity QED [Vlastakis et al., Science 342:607 (2013)]. These experiments do not implement our full protocol, but they demonstrate that conditional system–ancilla couplings and engineered Lindbladians of the relevant type are experimentally available ingredients. Related erasure-based architectures in superconducting and neutral-atom systems also show that hardware can be designed so that dominant noise channels are converted into structured and detectable forms [Kubica et al. PRX 13:041022 (2023); Teoh et al. PNAS 120:e2221736120 (2023); Levine et al. PRX 14:011051 (2024); Sahay et al. PRX 13:041013 (2023)].

In addition to discussing possible routes for realization, we highlight that our mitigation Lindbladian is not required to dominate the native noise by a large separation of rates. Rather, it has the same scaling, $\|L_{\text{mit}}\| = \|L_k \otimes \sigma_z\| \sim \|L_k\|$ (see also our response to **Reviewer 2**'s **Major Comment 2**). We further note that the protocol's robustness to system–ancilla noise was already discussed in SI of the previous submission, but was only mentioned briefly in the main manuscript and was not explicitly related to crosstalk effects. In the revised manuscript, we have now explained the origin of correlated system–ancilla noise as a possible crosstalk effect and have moved the corresponding discussion from SI to Methods, where it is treated in more detail with additional numerical simulation results. The discussion and analysis of other possible noise sources are addressed in our responses to the reviewer's **Major Comments 5 & 6**.

We believe that these revisions clarify the feasibility and practical requirements of the proposed scheme.

To address the reviewer's comment, we have made the following revision:

- "Physical Implantation" section has been expanded to provide a more detailed and experimentally relevant consideration of the proposed protocol.
- More detailed analysis on the correlated errors has been added in "Methods."
- We have added additional sections (V & VI) in SI to provide complete details of the noise analysis supported by extended numerical simulation.

Major Comment 3: Stochastic-Hamiltonian implementation
More importantly, in the numerical examples, the mitigation dynamics is implemented via stochastic Hamiltonian evolution. It reintroduces stochasticity at the implementation level – which is not preferred in any sense. Thus, the physical realisation may both weaken the advantage over PEC and blur the distinction between a purely analogue scheme and one that relies on engineered control resources.

Our response: We thank the reviewer for the insightful comment. While stochasticity reintroduced for the physical implementation of the mitigation dynamics could weaken our work's motivation to avoid quasi-probabilistic sampling of the PEC protocol, we still have a clear distinction between the stochasticity introduced in our scheme and the PEC approaches. This can be summarized as follows:

- *Trajectory-conditioned stochasticity* (PEC approaches, e.g., Refs [30, 31]): Post-processing depends on per-shot measurement records, and reconstruction errors arise from finite-statistics estimates of Pauli channel decomposition coefficients.
- *Ensemble-averaged stochasticity* (Our approach): Single-shot Hamiltonians are random, but no per-shot record is needed; the average is exactly the deterministic Lindbladian, and reconstruction errors arise only from finite-statistics estimates of the recovered observable $\langle A \otimes \sigma_x \rangle$.

In other words, our scheme does not require any information about the stochastic trajectories, but only the final expectation value of the joint observable is required to reconstruct the ideal observables. The conceptual difference between the two approaches and our protocol's advantage over the other were provided in our response to **Reviewer 2**'s **Major Comment 1**.

We also highlight that the stochastic-Hamiltonian implementation is not the only possible route for realizing the engineered Lindbladian proposed in this work. A more standard way (for a general Lindbladian, even without considering implicit stochasticity) to do this is to introduce an additional dissipative reservoir state. While this requires a more complicated system-ancilla-reservoir coupling, such an interaction has been proposed and realized in certain physical platforms, as we discussed in response to the reviewer's **Major Comment 2**.

To highlight the distinction between the stochastic-Hamiltonian implementation and PEC approaches, we have revised the "Physical Implementation" section by adding the following sentence:

"*We clarify that the stochasticity introduced to implement the mitigation Lindbladian is ensemble-averaged and therefore does not require a per-shot record of individual trajectories; in particular, no information about $\eta_k(t)$ for each trajectory is required. This is clearly distinct from the trajectory-conditioned stochasticity considered in PEC approaches, where the weight assigned to each stochastic trajectory is essential for reconstructing the mitigated dynamics.*"

Major Comment 4: About the implementation and assumptions.
Another main concern is that the protocol requires accurate calibration not only of the noise rates but also of the noise operators themselves (i.e. the jump operators L_k). The mitigation Lindbladian is explicitly constructed from these operators, so any structural mischaracterisation of the noise will directly affect the cancellation mechanism. While this requirement is not unique to the present proposal – quasi-probabilistic error mitigation methods also rely on detailed noise characterisation – it remains a strong assumption in the analogue setting, where precise identification of the full Lindblad structure may be challenging.

Our response: The reviewer is correct that the protocol requires precise identification of the dominant jump operators L_k that describe the dissipation. The protocol should therefore be understood as a noise-model-assisted QEM method, similar in spirit to other model-based mitigation approaches. For example, as the reviewer correctly pointed out, probabilistic error cancellation also requires a characterised noise map in order to construct an inverse quasiprobability representation, and recent scalable PEC implementations learn sparse Pauli-Lindblad generators and rates for this purpose [van den Berg et al., Nat. Phys. 19:1116 (2023)].

We agree with the reviewer that this is a strong assumption in analogue settings, where the full Lindblad structure may be difficult to identify. For the purposes of error mitigation, however, it suffices to identify the dominant dissipative channels. In this regard, our protocol makes the analogous assumption that the dominant dissipative channels L_k are either known from the physical noise mechanism or can be learned experimentally. To further address the reviewer's concern, we have added a discussion explaining how this assumption can be supported by existing noise-learning techniques that can be implemented across various physical platforms, along with relevant references (see also our response to **Reviewer 1**'s **Major Comment 3**).

We have added the subsection "Noise model requirements" to the revised manuscript to discuss the required knowledge of the dissipator in the broader QEM landscape and to describe feasible approaches for characterizing it, as follows:

"*The protocol requires a learned or physically motivated representation of the dominant jump operators L_k. This requirement is analogous to the noise-characterisation assumptions in other QEM methods. For example, PEC requires a characterised noise map in order to construct an inverse quasiprobability representation, and recent large-scale PEC implementations learn sparse Pauli-Lindblad noise models for this purpose. This can be done by master-equation reconstruction protocols, which have been demonstrated experimentally in NMR, trapped-ion, and superconducting-qubit platforms. When the relevant dissipator is sparse, local, or Pauli-twirled, the required information can be obtained by established characterisation methods such as randomized compiling, cycle benchmarking, and sparse Pauli-Lindblad learning. This can be further combined with recently proposed compressed-sensing or shadow-based Lindblad tomography.*"

We have also relocated the subsection "Protocol robustness against imperfect noise characterization" to appear before "Protocol robustness against ancilla noise" for better logical flow.

Major Comment 5: Ancilla noise and non-Markovianity.
In continuation, in comparison with quasi-probabilistic approaches, the system is coupled to an additional ancilla qubit, and the analysis assumes that ancilla errors can be modelled as a single-qubit Lindblad dissipator acting only on the ancilla, with rate Γ. At first glance, this appears to be a reasonable and standard modelling choice. However, upon closer inspection, this assumption may not always hold in practice.

In particular, ancilla errors may be induced by the engineered system–ancilla coupling itself and therefore need not be strictly local or independent of the system. Moreover, the analysis implicitly assumes:

1. No significant leakage out of the ancilla computational subspace;
2. Noise rates that are stationary or at least slowly varying so that they can be reliably estimated.

Indeed, this assumption on full Markovianity may not hold. For example, in solid-state computing, with partial swap with the reservoir, it can easily induce non-Markovianity. Moreover, this may not hold with additional stochasticity or coupling, see some recent discussions like emergence of non-Markovianity induced by either Pauli twirling, or reservoir entanglement.

Within this simplified model, the paper shows that ancilla noise contributes only an additional exponential decay term of the form $-\nu\Gamma W_{01}$, which can be compensated through rescaling. Though I suspected, this conclusion may rely on the dissipative Lindblad structure.

Our response: As the reviewer correctly pointed out, the exact cancellation derived in the main text relies on a time-local Markovian Lindblad description of the engineered system–ancilla dynamics. However, in more realistic situations, non-Markovianity naturally arises from system–reservoir correlations [Yosifov et al., arXiv:2507.21907; Strachan-Purkayastha-Clark, Phys. Rev.

Lett. 134:220403 (2025); Agustí et al., Quantum 10:2066 (2026)], or from additional tailoring of the noise, for example, through Pauli twirling [Kattemölle-Gulácsi-Burkard, arXiv:2602.08464]. We have therefore revised the manuscript to discuss when the system goes beyond this Markovianity assumption in the following three different cases: time-dependent Markovian rates, weak bath-memory corrections, and genuinely non-Markovian dynamics.

First, if the dynamics remains time-local and Lindbladian but the rates drift slowly in time, the derivation is unchanged after replacing at by the integrated rate

$$\bar{a}(t) = \int_0^t a(s)\, ds.$$

In this case, the rescaling factor can be calibrated or updated from the measured time-dependent rates. Thus, stationarity of the rates is not essential; what is essential is that the dynamics remains well described by a time-local Lindblad generator over the mitigation window.

Second, genuine bath memory should be regarded as a model-mismatch effect. If the bath has a finite correlation time τ_B, the jump process is no longer exactly a memoryless Poisson process, and the cancellation is no longer exact at the level of a Lindblad semigroup. When the bath memory is short on the scale of the engineered dissipation, $a\tau_B \ll 1$, the resulting bias is perturbative, with corrections controlled by the small parameter $a\tau_B$ and accumulated over the evolution time. In contrast, when $a\tau_B \gtrsim 1$, for example in a strong partial-swap regime with the reservoir, the bath memory can interfere with the heralding mechanism and a fully non-Markovian treatment would be required. We now present this in the subsection of Methods as a quantitative applicability criterion rather than as a blanket robustness claim.

Finally, ancilla errors induced by the engineered coupling, leakage out of the ancilla computational subspace, and correlated system–ancilla noise are implementation errors that need not be captured by an independent single-qubit ancilla Lindblad term. While the protocols' robustness against correlated system–ancilla noise was already presented in the previous SI, we have now moved the discussion to Methods and explicitly relate it to crosstalk (see also our response to **Reviewer 2**'s **Major Comment 2**). We also explain in the revised SI that encoded ancilla variants can suppress specific leakage or false-parity-flip errors; these are optional robustness extensions and are not part of the bare no-postselection protocol.

In response to the reviewer's comment,

- We have added the "Non-Markovianity effects" subsection to Methods to describe possible sources of non-Markovianity and the regime in which our protocol remains valid.
- We have now explicitly discussed correlated system–ancilla noise as a possible crosstalk effect in the subsection of Methods.
- In the revised SI (Sec. V), we have added a discussion on handling leakage out of the ancilla subspace by combining our protocol with an encoded ancilla register based on error-correcting or error-detecting codes.

Major Comment 6: Coherent ancilla errors.
A more problematic issue is the coherent (Hamiltonian) errors on the ancilla. A systematic ancilla detuning or unwanted rotation introduces additional Hamiltonian terms H_{anc}. Such coherent errors do not merely rescale the damping factor; instead, they can rotate the encoded off-diagonal block that carries the ideal state information. This may invalidate the simple recovery formula.

Our response: We have analysed this concern carefully and reach a more optimistic conclusion than the reviewer anticipates, with two key results:

(a) The bare-ancilla protocol is naturally first-order robust. Because the ancilla is initialised in a +1 eigenstate of the recovered observable σ_x, first-order corrections in any single-axis ancilla detuning vanish by symmetry. The leading bias is *quadratic* in the detuning rate, $O(\delta^2)$, not linear. More precisely, this situation can be generally modelled by the misalignment between the ancilla state and the final measurement axis σ_x in the Heisenberg picture, as

$$\tilde{\sigma}(\delta) = R^\dagger(\delta)\sigma_x R(\delta) = (\cos\delta)\sigma_x + (\sin\delta)\sigma_{\hat{n}}, \tag{2}$$

where $R^\dagger(\delta)$ is the rotation unitary of the ancilla state with the misalignment angle δ, and $\hat{n}$ is a vector orthogonal to the x-axis, i.e., $\mathrm{Tr}[\sigma_x\sigma_{\hat{n}}] = 0$. In this case, the misaligned observable at the final measurement becomes $A \otimes \tilde{\sigma}(\delta)$ instead of $A \otimes \sigma_x$. We show that the expectation value of $A \otimes \tilde{\sigma}(\delta)$ of the system ancilla joint state after the mitigation dynamics becomes

$$\mathrm{Tr}[(A \otimes \tilde{\sigma}(\delta))W(t)] = (\cos\delta)e^{-2at}\langle A(t)\rangle_{\text{ideal}}, \tag{3}$$

where we only have the additional factor $\cos\delta$ compared to the coherent-error-free case. For $\delta \ll 1$, we have $\cos\delta \approx 1-\delta^2/2$, so that the leading order of correction becomes $\mathcal{O}(\delta^2)$ with fully suppressed first-order contribution, even without any further actions.

This phenomenon can be more generally understood based on the symmetry between the generator of the ancilla state and the final measurement. The key feature is that as the stabiliser of the ancilla's initial state ($|+\rangle$) and the final measurement are both given as $\sigma_x^{(a)} = g$, any coherent perturbation of the stabiliser $\tilde{\sigma}(\delta) = e^{i\delta Q} g e^{-i\delta Q} = g + i\delta[Q, g] + \mathcal{O}(\delta^2)$ with a Hermitian operator Q, the linear term is fully suppressed as $\mathrm{Tr}(g[Q, g]) = 0$ for any Q. This provides the first-order robustness of the coherent ancilla miscalibration given by any rotation. To refer back to a comparison made by Reviewer 2, both Ref [30]'s signed-recovery PEC and our bare-ancilla protocol fall in this class.

(b) Distance-2 ancilla error detection further reduces the bias prefactor. A parity-checked encoding of the ancilla into a stabiliser code that detects weight-1 ancilla errors (e.g. $|0_L\rangle = |00\rangle$, $|1_L\rangle = |11\rangle$ with stabiliser Z_1Z_2, or the [[4,2,2]] code) preserves the δ^2 scaling but reduces its coefficient by an additional $\sim 11\times$ constant prefactor at *constant simulator cost.* We have verified numerically that the bias continues to scale as δ^2 rather than δ^4 under both codes in the revised SI. The reason that stabiliser-distance arguments do *not* lift to δ^4 in this continuous-time setting is structural: continuously-on coherent ancilla detuning perturbs the heralding mechanism itself throughout the evolution, producing an $O(\delta^2)$ bias that error *detection* cannot remove without disrupting the herald. Reaching δ^4 would require either error-correcting (rather than error-detecting) ancilla codes, or discrete-time herald refresh – both of which trade off against the protocol's continuous-time character; we leave these as open directions.

We have also verified that the post-selection associated with the $d = 2$ check does not reduce the per-shot variance: the $d = 2$ estimator's per-accepted variance is essentially identical

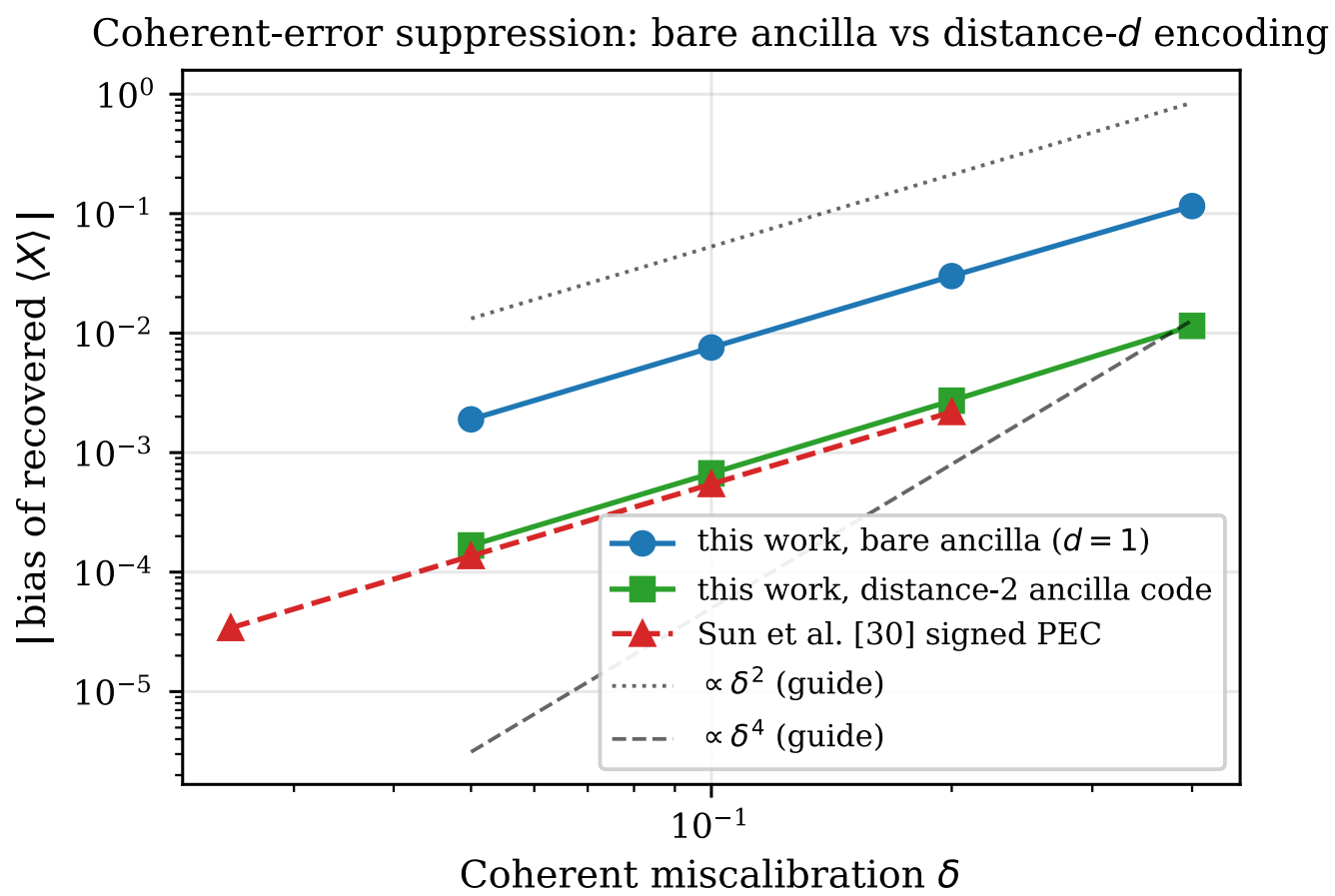

Figure 3: Coherent-error suppression: bias of the recovered observable vs. miscalibration angle δ, at $\gamma T = 0.3$. The bare-ancilla protocol scales as δ^2; the distance-2 error-detecting code (parity-checked ZZ; equivalent to [[4,2,2]] for this purpose) scales as δ^2 with a $\sim 11\times$ smaller prefactor; Sun et al. [30]'s signed-recovery PEC also scales as δ^2 at this benchmark. The structural advantage of our scheme is that the ancilla-side error detection is at *zero simulator cost*.

to the bare estimator's variance at small δ, with the only effective-sample-cost penalty being the $1/p_{\text{accept}} = 1 + O(\delta^2)$ acceptance-rate factor. The $d = 2$ gain is a bias-prefactor reduction at neutral variance cost, not a variance-axis exponent reduction. The variance-axis exponent reduction *is* achievable, separately, by the multi-flag block-resolved heralding, but the mechanism is orthogonal: it resolves the noise commutant rather than encoding the ancilla. Using a coherent ancilla detuning generated by σ_y on the ancilla (rotating the recovered σ_x into the dissipator-visible σ_z basis – the worst-case axis), we obtain at $\gamma t = 0.6$ as shown in Fig. 3.

Connection to erasure-converted qubits. Within the erasure-channel framework, a coherent ancilla error plays the role of an *unflagged residual error* on the heralding register – a well-studied failure mode in erasure-converted superconducting and neutral-atom qubits, whose suppression via stabiliser-encoded ancillas is established in [Kubica et al., PRX 13:041022 (2023); Sahay et al., PRX 13:041013 (2023); Levine et al., PRX 14:011051 (2024)]. Our distance-2 error-detecting ancilla extension is the continuous-time analogue of these constructions, and the $\sim 11\times$ prefactor reduction is the corresponding biased-erasure threshold improvement at $d = 2$.

- We have added a subsection to Methods describing the vanishing of the first-order contribution from coherent ancilla errors, which leads to δ^2 scaling. We also discuss the possibility of further suppressing coherent errors by combining the protocol with error-correction/error-detection codes.

- In the revised SI, we have added a section entitled "Coherent Ancilla Miscalibration and Encoded-Ancilla Checks," which provides a more general discussion of the protocol's first-order robustness to coherent ancilla error. In the same section, we have introduced the [[4,2,2]]-code detection schemes and numerically demonstrated the constant-prefactor improvement at zero simulator cost.

Concluding Remarks:
Overall this work is well written and I find the central idea conceptually stimulating. Despite the concerns outlined above, the work represents a meaningful contribution to the theory of error mitigation in analogue quantum simulation. The continuous-time perspective and the dissipative encoding mechanism are novel and likely to stimulate further discussion. I would like to reconsider its publication if the authors address my concerns raised above. Alternatively, the authors may consider npj Quantum Information, which appears to be a suitable venue for this work.

Our response: We thank the reviewer for the positive assessment of the manuscript and for recognising the conceptual novelty of the continuous-time perspective and the dissipative encoding mechanism. We are also grateful for the reviewer's willingness to reconsider the manuscript upon revision.

We appreciate the reviewer's suggestion that npj Quantum Information could be a suitable venue. Nevertheless, given the revisions above and the protocol's connections to error mitigation, analogue quantum simulation, continuous-time algorithm design, and ancilla-assisted dissipative control, we respectfully believe that the revised manuscript is well aligned with the scope and standards of the present journal. We thank the reviewer again for the constructive comments, which have significantly improved the clarity, scope, and technical depth of the work.

Authors’ response to referees’ comments.

Following Reviewer 3’s comment, we have removed the arguments about the differences between PEC and our approach using stochastic Hamiltonian implementation.)
